# Regioselective O/C phosphorylation of α-chloroketones: a general method for the synthesis of enol phosphates and β-ketophosphonates *via* Perkow/Arbuzov reaction†

**DOI:** 10.1039/d0ra05140c

**Published:** 2020-08-11

**Authors:** Yuepeng Cao, Zhenhua Gao, Junchen Li, Xiaojing Bi, Ling Yuan, Chengxin Pei, Yongbiao Guo, Enxue Shi

**Affiliations:** State Key Laboratory of NBC Protection for Civilian Beijing 102205 P. R. China van87120@126.com exshi@sina.com

## Abstract

A regioselective O/C phosphorylation of α-chloroketones with trialkyl phosphites was performed for the first time, which employed solvent-free Perkow reaction and NaI-assisted Arbuzov reaction under mild conditions respectively. Versatile enol phosphates were prepared in good to excellent yields as well as β-ketophosphinates.

## Abstract 2

A highly regioselective O/C phosphorylation of α-chloroketones with trialkyl phosphites was developed in the preparation of enol phosphates and β-ketophosphonates.

## Graphical abstract

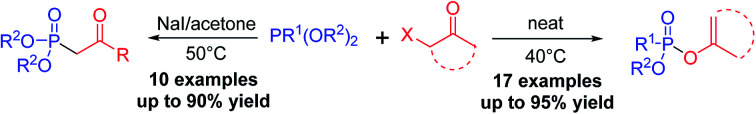

## Introduction

**Scheme 1:**
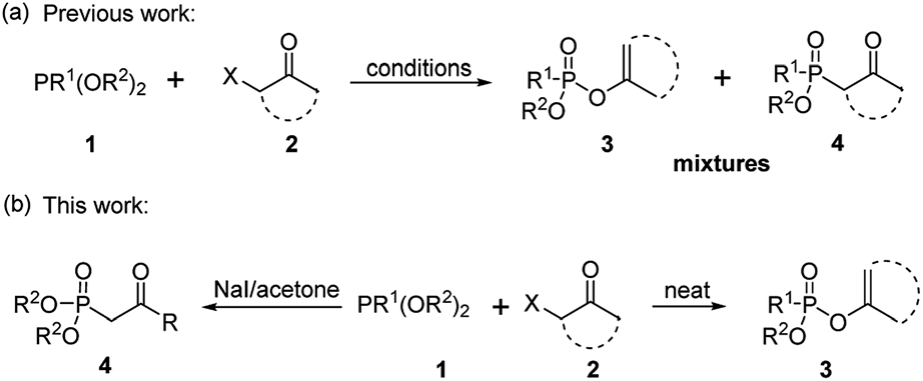
Previous (a) and this work (b) for synthesis of enol phosphates and β-ketophosphonates.

**Fig. 1:**
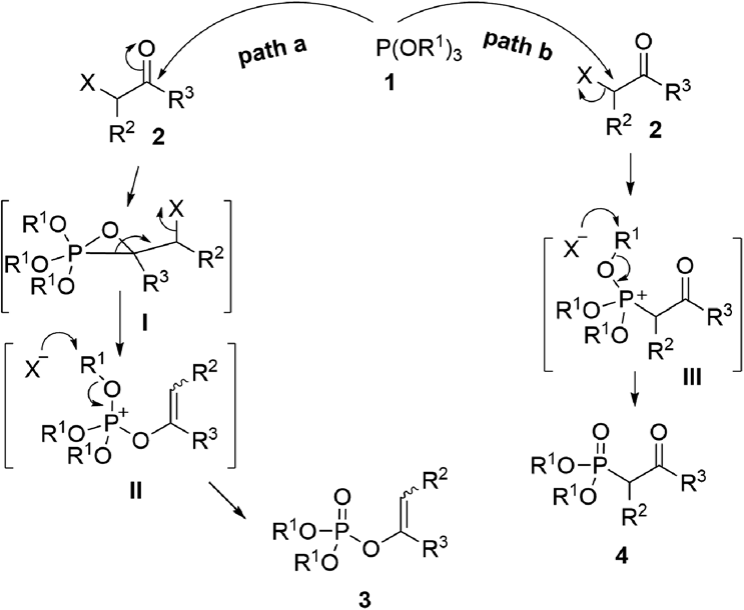
Proposed mechanistic pathway.

As one of the most utilized classic reactions, Perkow reaction^1,2^ offers an efficient way to obtain pharmacologically and biologically important enol phosphates through simple condensation of trialkyl phosphites and α-haloketones.^3–13^ However, the reaction generally gives a mixture of enol phosphates and β-ketophosphonates generating from the competitive O/C phosphorylation (Scheme 1a), which proceeds mainly through the following two possible reaction pathways of α-haloketones (Fig. 1): (i) path a, the nucleophilic P atom of trialkyl phosphite attacks the carbonyl C atom of α-haloketone forming a triatomic heterocyclic I which followed by the ring cleavage transforms into a phosphonium salt II and then by the *O*-demethylation gives the enol phosphates product 3 (Perkow reaction mechanism); (ii) path b, the nucleophilic P atom attacks the halocarbon of α-haloketone to generate a phosphonium salt III, followed by the *O*-demethylation to give the β-ketophosphonate product 4 (Arbuzov reaction mechanism).^14–19^ To the date, highly regioselective O/C phosphorylation of α-haloketones with trialkyl phosphites remains unavailable. Herein, we wish to report a simple but efficient method for regiospecific synthesis of enol phosphates and β-ketophosphonates respectively with good to excellent yields under quite mild conditions only by using the most easily-available and economic α-chloroketones as the starting metarial (Scheme 1b).

## Results and discussion

Examination and optimization of the reaction parameters were explored using trimethyl phosphite 1a and 1-bromopropan-2-one 2a′ as the model substrates. The reaction without solvent used at 40 °C proceeded to give enol phosphate 3a and β-ketophosphonate 4a in 50% yield respectively (Table 1, entry 1). Encouraged by this result, a systematic screening of the reaction conditions was carried out to examine the regioselectivity (Table 1). A solvent screen revealed that lower-polar solvents could indeed facilitate the regioselectivity of Perkow reaction (entries 2–7), whereas higher-polar solvent could favour the occuration of Arbuzov reaction (entries 7–11). And the highest regioselectivity (7 : 1) for Perkow reaction was obtained in *p*-xylene with yields of 45%. Based on the knowledge of the mechanism of Perkow reaction, we assumed that using 1-chloropropan-2-one 2a instead of 2a′ should improve the selectivity. Unfortunately, the reaction became very sluggish (entry 12). But unexpectedly, the solvent-free reaction of 1a and 2b gave the 3a not only in good yield but with excellent selectivity (entry 13). Moreover, when lowering the temperature just from 40 to 30 °C, the regioselectivity could increase to as high as 94 : 1 (entry 14) though the reaction became some lower reactive (50% yield *vs.* 70%). Raising the reaction temperature to 50 °C or 90 °C, which prospectively improved the reaction rate significantly, would obviously decrease the selectivity (entries 15 and 16). Further control experiments revealed that the optimized reaction conditions should be usage of 1.2 equiv. of 1a, providing the target compound in 80% yield with 50 : 1 regioselectivity (entry 17).

**Table 1:** Optimization of the Perkow reaction conditions^a^

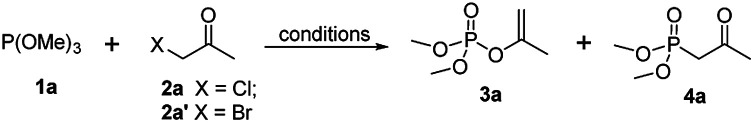						
Entry	X	Temp. (°C)	Solvent	T (h)	Yield (%) of 3a^b^	3a : 4a^b^
1	Br	40	—	3	50	1 : 1
2	Br	40	*p*-Xylene	72	45	7 : 1
3	Br	40	*o*-Xylene	72	45	6.5 : 1
4	Br	40	*m*-Xylene	72	40	4.5 : 1
5	Br	40	PhMe	72	30	4.5 : 1
6	Br	40	PhH	72	15	2.5 : 1
7	Br	40	CH_2_Cl_2_	72	50	3 : 1
8	Br	40	Et_2_O	72	15	2 : 1
9	Br	40	THF	72	20	2 : 3
10	Br	40	1,4-Dioxane	72	30	2 : 3
11	Br	40	Acetone	72	25	2 : 5
12	Cl	40	*p*-Xylene	36	Trace	—
13	Cl	40	—	36	70	50 : 1
14	**Cl**	**30**	**—**	**36**	**50**	**94 : 1**
15	Cl	50	—	24	73	28 : 1
16	Cl	90	—	10	70	12 : 1
17^c^	Cl	40	—	36	80	50 : 1
18^d^	Cl	40	—	36	80	50 : 1

a Reaction conditions: 1a (2.4 mmol), 2a or 2a′ (2 mmol), in neat or 5 mL solvent.

b Yield and regioselectivity of 3a and 4a were determined by ^1^H NMR.

c 1.2 equiv. of 1a used.

d 1.5 equiv. of 1a used.

**Scheme 2:**
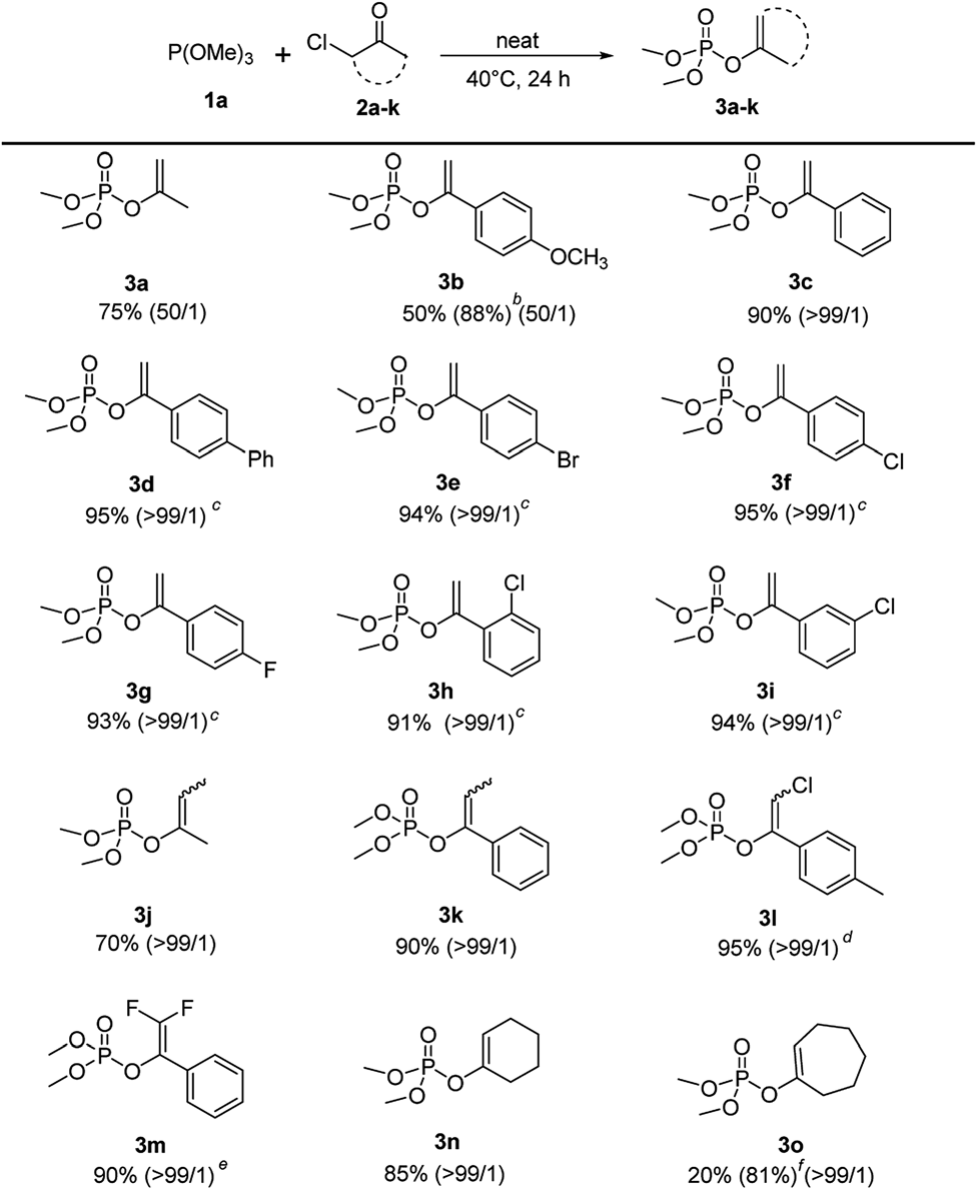
Scope of α-haloketones.^*a a*^Reaction conditions: 1a (2.4 mmol), 2a–o (2 mmol), 40 °C, 24 h. ^*b*^1a (6 mmol). ^*c*^10 h. ^*d*^2 h. ^*e*^1 h. ^*f*^90 °C, 48 h.

Under the optimized conditions, the scope of the regioselective Perkow reaction of trimethyl phosphite 1a and various α-chloroketone (2a–k) was investigated (Scheme 2). Generally, both the steric and electronic substituents on aliphatic and aromatic α-chloroketones seem to have negligible effects on the regioselectivity. Substrates bearing either an electron-donating (OMe) or electron-withdrawing groups (Br, Cl and F) at the 4-position of the phenyl ring were all converted to their corresponding products 3b–3g in excellent yields (88% to 95%) with high regioselectivities (50 : 1 to >99 : 1), albeit the α-chloroketone with electron-donating OMe required a higher ratio of 1a : 2b (3 : 1). However, the electronic effects may have great influence on the reaction rate leading to the reaction times of α-chloroketone with electron-withdrawing group (Ph, Br, Cl and F) significantly reduced to 10 h. But the positions of substituents on phenyl ring showed almost no effect (entry 3h and 3i*vs.*3f). It is worth noting that the electronic substituents on the halocarbon C could significantly determine the reaction efficiency as shown by chloro-substituted (3l) and 2,2-difluoro-substituted (3m) substrates with only 2 h and 1 h reaction time, while the methyl-substituted substrates (3j) and (3k) with 48 h and 24 h reaction time. For cyclical substrates, the corresponding products were obtained in excellent regioselectivities but much different yields (3n) and (3o) under the optimized conditions. Gratifyingly, when the reaction temperature was increased 90 °C, 81% yield without loss of regioselectivity could be achieved after 48 h reaction time (3o).

Encouraged by those results, we next optimized the Arbuzov reaction conditions (Table 2). Although compared with 1-chloropropan-2-one and 1-bromopropan-2-one, 1-iodopropan-2-one exhibited superior regioselectivity (25 : 1) of Arbuzov reaction, the yield of 4a was poor (entry 3 *vs.* entries 1 and 2) due to that 1-iodopropan-2-one would decompose under solvent-free conditions. However, since higher-polar solvent of actone exhibited good regioselectivity of Arbuzov reaction (Table 1, entry 11), we hypothesized that if 1-iodoketone could be generated *in situ* by treatment of 2a with NaI in acetone (Finkelstein reaction^20–23^), which may be beneficial to the conversion favouring Arbuzov reaction. Following experiments verified our assumption that 4a was obtained in 85% yield with 26 : 1 regioselectivity (entry 5). Further screening of reaction temperature revealed that 50 °C was the most satisfactory condition (entry 7, 85%, 35 : 1).

**Table 2:** Optimization of the Arbuzov reaction conditions^a^

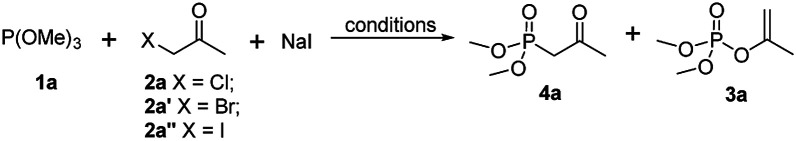							
Entry	X	Temp. (°C)	NaI	Solvent	T (h)	Yield (%) of 4a^b^	4a : 3a^b^
1	Cl	40	—	—	36	<2	1 : 50
2	Br	40	—	—	3	50	1 : 1
3	I	40	—	—	2	25	25 : 1
4	Br	40	—	Acetone	30	63	5 : 2
5	Cl	40	1 eq.	Acetone	4	85	26 : 1
6	Cl	45	1 eq.	Acetone	2	84	28 : 1
7	**Cl**	**50**	**1 eq.**	**Acetone**	**2**	**85**	**35 : 1**
8	Cl	60	1 eq.	Acetone	2	87	24 : 1

a Reaction conditions: 1a (2.4 mmol), 2a, 2a′ or 2a′′ (2 mmol), NaI (0 or 2 mmol) neat or 5 mL acetone, 30–60 °C, 2–30 h.

b Yield and regioselectivity of 4a and 3a were determined by ^1^H NMR.

**Scheme 3:**
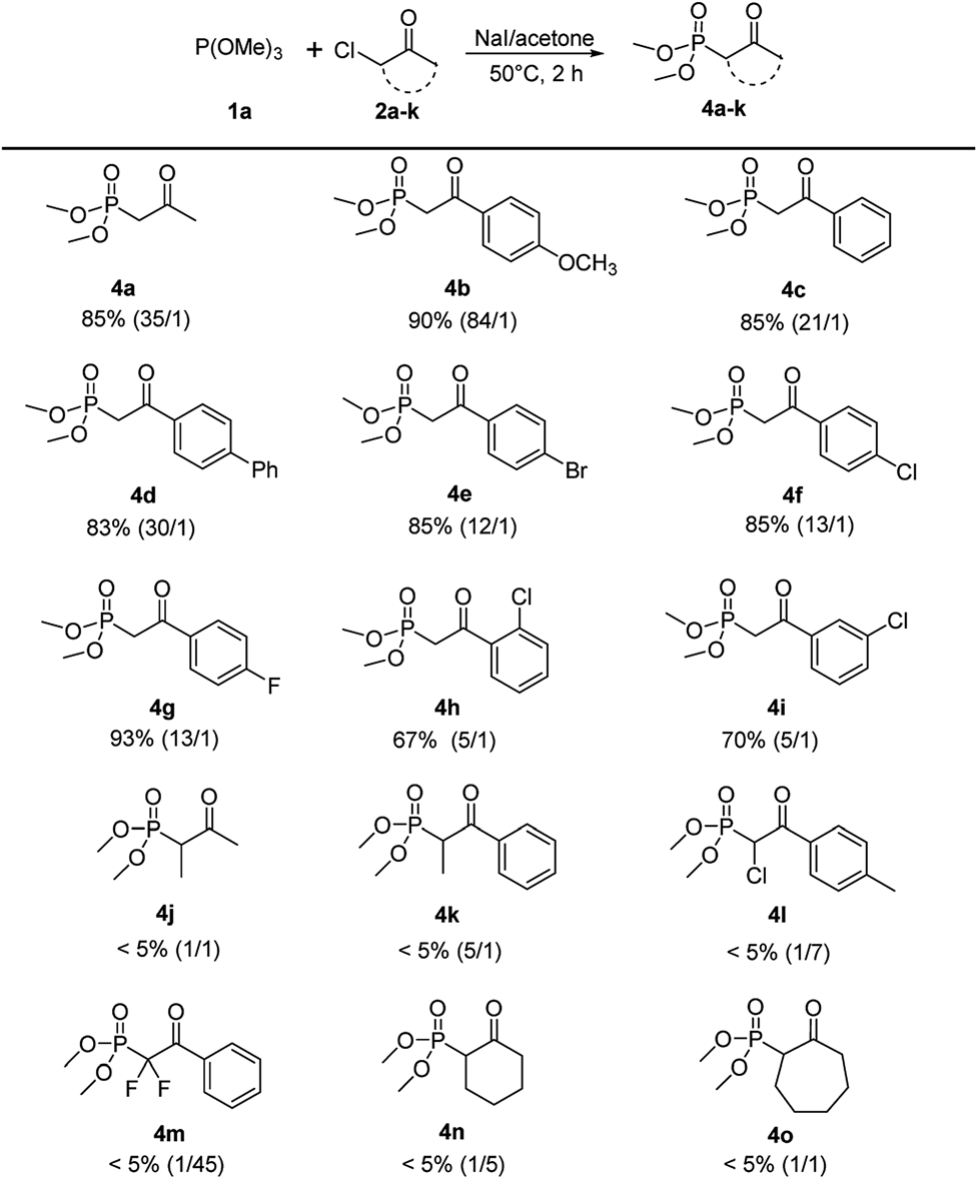
Scope of α-haloketones.^*a a*^Reaction conditions: 1a (2.4 mmol), 2a–k (2 mmol), NaI (2 mmol), 5 mL acetone, 50 °C, 2 h.

With the optimized reaction conditions in hand, the scope of this reaction was explored with trimethyl phosphite 1a and various α-chloroketones (2a–k) to prepare β-ketophosphonates 4a–k (Scheme 3). Substrates bearing either an electron-donating (OMe) or electron-withdrawing groups (Br, Cl and F) at the 4-position on the phenyl ring were converted to their corresponding Arbuzov products 4b–4i in good to excellent yields (67% to 93%) and moderate to excellent regioselectivities (5 : 1 to 84 : 1). Notably, the electronic effects and the positions of substituents on the phenyl ring have some effect on the selectivity. For example, electron-donating and 4-position groups on the phenyl ring were more beneficial (4b*vs.*4e–g and 4f*vs.*4h,i) for Arbuzov reaction. Encouraged by these results, we next tested the generality of the reaction regarding the substituent groups on the halocarbon of α-haloketones. However, the reaction proceeded sluggishly and only trace of the product 4j–4o were detected. It was analyzed that the steric effects of substituents have a significantly effect on the *in situ* iodation reaction.

**Scheme 4:**
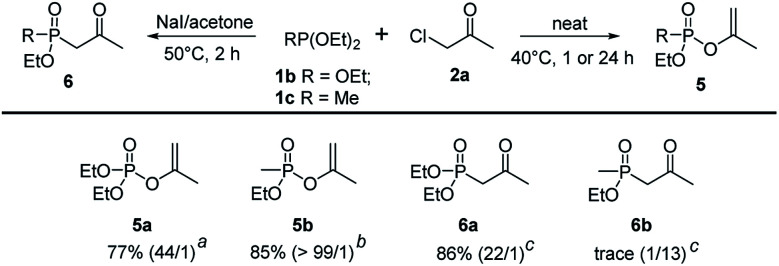
Scope of phosphites.^*a*^ Reaction conditions: ^*a*^1b (2.4 mmol), 2a (2 mmol), 40 °C, 24 h. ^*b*^1b (2.4 mmol), 2a (2 mmol), 40 °C, 1 h. ^*c*^1b (2.4 mmol), 2a (2 mmol), NaI (2 mmol), 5 mL acetone, 50 °C, 2 h.

To further extend the substrate scope, triethyl phosphite 1b and diethyl methylphosphonite 1c were examined in the regioselective Perkow : Arbuzov reaction system. Under the optimized conditions, triethyl phosphite gave its corresponding Perkow product 5a and Arbuzov product 6a in good yields (77% and 86%) and regioselectivities (44 : 1 and 22 : 1) respectively, which was similar to trimethyl phosphite 1a. It was noteworthy that the reaction activity of 1c was much greater than 1b (1 h *vs.* 24 h) giving product 5b in 85% yield with very high regioselectivity (>99 : 1). However, the substrate 1c gave disappointingly trace amount of 6b under the standard reaction conditions of Arbuzov reaction (Scheme 4).

## Conclusions

In summary, regioselective synthesis of enol phosphates and β-ketophosphonates *via* Perkow/Arbuzov reaction of phosphites only with α-haloketones in good to excellent yields has been firstly achieved at our laboratory. The success of methylphosphonite also suggests that phosphinites and amino phosphites, as well as α-halothioketones, may be also applicable in this reaction system in the future study.

## Experimental section

### General information

Reagents and solvents were purchased from common commercial suppliers and were used without further purification. Column chromatography was generally performed on silica gel (200–300 mesh). Melting points were determined with a Büchi B-545 melting-point apparatus. 600 MHz ^1^H NMR and 150 MHz ^13^C NMR spectra were recorded on Varian VMS-600 spectrometers respectively. The chemical shifts are reported in ppm (*δ* scale) relative to internal tetramethylsilane, and coupling constants are reported in hertz (Hz). High-resolution mass spectra (HRMS) were obtained on a Agilent 6502 Q-TOF HPLC and mass spectrometry.

### General procedure for the synthesis of enol phosphates 3

A mixture of 1 (2.4 mmol) and 2 (2 mmol) was stirred at 40 °C under nitrogen atmosphere for 1–48 h and then concentrated under reduced pressure. The resulting orange gum was purified by column chromatography on a silica gel column [eluting with PE : i-PrOH (20 : 1)] to obtain 3 as colorless oil or white solid.

### General procedure for the synthesis of β-ketophosphonates 4

A mixture of 1 (2.4 mmol), 2 (2 mmol) and NaI (2 mmol) in dry actone was stirred at 50 °C under nitrogen atmosphere for 2 h and then concentrated under reduced pressure. The resulting orange gum was purified by column chromatography on a silica gel column [eluting with PE : i-PrOH (10 : 1)] to obtain 4 as colorless oil or white solid.

## Conflicts of interest

There are no conflicts to declare.

## Supplementary Material

RA-010-D0RA05140C-s001
