# Supplementary material for: Regioselective O/C phosphorylation of α-chloroketones: a general method for the synthesis of enol phosphates and β-ketophosphonates via Perkow/Arbuzov reaction
Source: RSC Adv. 2020 Aug 11;10(49):29493–7. doi: 10.1039/d0ra05140c (PMC9055934; doi:10.1039/d0ra05140c)
Supplement: RA-010-D0RA05140C-s001 [file RA-010-D0RA05140C-s001.pdf]

Electronic Supplementary Material (ESI) for RSC Advances.
This journal is © The Royal Society of Chemistry 2020

Regioselective O/C Phosphorylation of α-Chloroketones: A General Method for the Synthesis of Enol Phosphates and β-Ketophosphonates via Perkow/Arbuzov Reaction

Yuepeng Cao, Zhenhua Gao, Junchen Li, Xiaojing Bi, Ling Yuan, Chengxin Pei, Yongbiao Guo*, Enxue Shi*

State Key Laboratory of NBC Protection for Civilian, Beijing 102205, P. R. China
*E-mail: van87120@126.com; exshi@sina.com

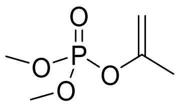

3a

dimethyl prop-1-en-2-yl phosphate (**3a**). Colorless oil, yield 75%; ^{1}H NMR(600 MHz, CDCl$_3$): δ (ppm) 4.72 (s, 1H), 4.48 (s, 1H), 3.78 (d, J = 11.3 Hz, 6H), 1.91 (s, 3H). ^{13}C NMR(151 MHz, CDCl$_3$): δ (ppm) 152.0 (d, J = 8.4 Hz), 98.1 (d, J = 4.9 Hz), 54.5 (d, J = 6.2 Hz) (2), 20.5 (d, J = 5.0 Hz). ^{31}P NMR(243 MHz, CDCl$_3$): δ (ppm) -4.6. HRMS (m/z) calcd for $C_5H_{12}O_4P[M+H]^+$ 167.04732, found 167.04636.

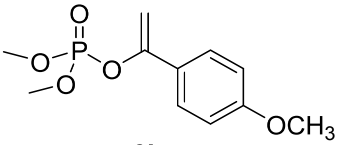

3b

1-(4-methoxyphenyl)vinyl dimethyl phosphate (**3b**). Colorless oil, yield 88%; ^{1}H NMR(600 MHz, CDCl$_3$): δ (ppm) 7.68-7.41 (m, 1H), 6.87 (d, J = 8.9 Hz, 1H), 5.16 (t, J = 2.7 Hz, 1H), 5.12-5.04 (m, 1H). 3.83 (d, J = 11.3 Hz, 6H), 3.80 (s, 3H). ^{13}C NMR(151 MHz, CDCl$_3$): δ (ppm) 160.2, 152.0 (d, J = 7.8 Hz), 126.6, 113.7, 95.6 (d, J = 3.6 Hz), 55.2, 54.8 (d, J = 6.1 Hz) (2). ^{31}P NMR(243 MHz, CDCl$_3$): δ (ppm) -4.0. HRMS (m/z) calcd for C11H16O5P[M+H]+ 259.07354, found 259.07237.

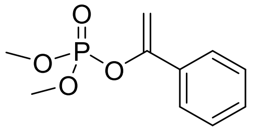

3c

dimethyl (1-phenylvinyl) phosphate (**3c**). Colorless oil, yield 90%; ^{1}H NMR(600 MHz, CDCl$_3$): δ (ppm) 7.64-7.53 (m, 2H), 7.40-7.29 (m, 3H), 5.29 (t, *J* = 2.8 Hz, 1H), 5.21 (dd, J = 2.9, 2.2 Hz, 1H), 3.83 (d, J = 11.3 Hz, 6H). ^{13}C NMR(151 MHz, CDCl$_3$): δ (ppm) 152.1 (d, J = 7.8 Hz), 133.9 (d, J = 6.8 Hz), 129.1, 128.3 (2), 125.0 (2), 97.3 (d, J = 3.5 Hz), 54.78 (d, J = 6.1 Hz) (2). ^{31}P NMR(243 MHz, CDCl$_3$): δ (ppm) -4.0. HRMS (m/z) calcd for $C_{10}H_{14}O_4P[M+H]^+$ 229.06297, found 229.06194.

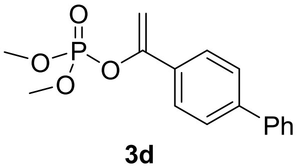

3d

1-([1,1'-biphenyl]-4-yl)vinyl dimethyl phosphate (**3d**). Colorless oil, yield 95%; ^{1}H NMR(600 MHz, CDCl$_3$): δ (ppm) 7.67 (d, J = 8.4 Hz, 2H), 7.64-7.56 (m, 3H), 7.45 (t,

J = 7.7 Hz, 2H), 7.37 (t, J = 7.4 Hz, 1H), 5.36 (t, J = 2.8 Hz, 1H), 5.30-5.21 (m, 1H), 3.88 (d, J = 11.3 Hz, 6H). ^{13}C NMR(151 MHz, $CDCl_3$): δ (ppm) 152.0 (d, J = 7.7 Hz), 142.0, 140.3, 132.9 (d, J = 6.8 Hz), 128.8, 127.7, 127.1 (2), 127.0 (2), 125.6 (2), 97.4 (d, J = 3.6 Hz), 54.9 (d, J = 6.1 Hz) (2). ^{31}P NMR(243 MHz, $CDCl_3$): δ (ppm) -4.0. HRMS (m/z) calcd for $C_{16}H_{18}O_4P[M+H]^+$ 305.09427, found 305.09266.

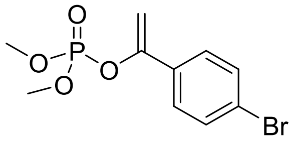

3e

1-(4-bromophenyl)vinyl dimethyl phosphate (**3e**). Colorless oil, yield 94%; ^{1}H NMR(600 MHz, $CDCl_3$): δ (ppm) 7.48 (d, J = 8.5 Hz, 2H), 7.44 (d, J = 8.7 Hz, 2H), 5.29 (t, J = 2.8 Hz, 1H), 5.24-5.21 (m, 1H), 3.84 (d, J = 11.3 Hz, 6H). ^{13}C NMR(151 MHz, $CDCl_3$): δ (ppm) 151.2 (d, J = 7.6 Hz), 132.9 (d, J = 6.8 Hz), 131.5 (2), 126.6 (2), 123.2, 97.9 (d, J = 3.6 Hz), 54.8 (d, J = 6.1 Hz) (2). ^{31}P NMR(243 MHz, $CDCl_3$): δ (ppm) -4.0. HRMS (m/z) calcd for $C_{10}H_{13}BrO_4P[M+H]^+$ 306.97348, found 306.97174.

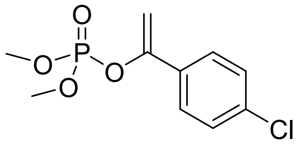

3f

1-(4-chlorophenyl)vinyl dimethyl phosphate (**3f**). Colorless oil, yield 95%; ^{1}H NMR(600 MHz, $CDCl_3$): δ (ppm) 7.52-7.44 (m, 2H), 7.36-7.26 (m, 2H), 5.30-5.14 (m, 2H), 3.81 (d, J = 11.4 Hz, 6H). ^{13}C NMR(151 MHz, $CDCl_3$): δ (ppm) 151.1 (d, J = 7.6 Hz), 135.0, 132.4 (d, J = 6.8 Hz), 128.5 (2), 126.4 (2), 97.8 (d, J = 3.6 Hz), 54.8 (d, J = 6.1 Hz) (2). ^{31}P NMR(243 MHz, $CDCl_3$): δ (ppm) -4.0. HRMS (m/z) calcd for $C_{10}H_{13}ClO_4P[M+H]^+$ 263.02400, found 263.02278.

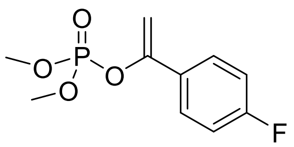

3g

1-(4-fluorophenyl)vinyl dimethyl phosphate (**3g**). Colorless oil, yield 93%; ^{1}H NMR (600 MHz, $CDCl_3$): δ (ppm) 7.64-7.48 (m, 2H), 7.05 (t, J = 8.7 Hz, 2H), 5.24 (t, J = 2.8 Hz, 1H), 5.21-5.19 (m, 1H), 3.86 (d, J = 11.3 Hz, 6H). ^{13}C NMR (151 MHz, $CDCl_3$): δ (ppm) 163.2 (d, J = 2489.2 Hz), 151.31 (d, J = 7.7 Hz), 130.2 (dd, J = 6.8, 3.3 Hz), 127.1 (d, J = 8.4 Hz) (2), 115.3 (J = 21.8 Hz) (2), 97.2 (dd, J = 3.6, 1.5 Hz),

54.9, 54.8. ^{31}P NMR (243 MHz, $CDCl_3$): δ (ppm) -4.1. HRMS (m/z) calcd for $C_{10}H_{13}FO_4P[M+H]^+$ 247.05355, found 247.05234.

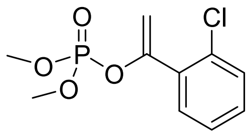

3h

1-(2-chlorophenyl)vinyl dimethyl phosphate (**3h**). Colorless oil, yield 91%; ^{1}H NMR(600 MHz, $CDCl_3$): δ (ppm) 7.48-7.46 (m, 1H), 7.40-7.38 (m, 1H), 7.30-7.23 (m, 2H), 5.51-5.29 (m, 1H), 5.16-4.94 (m, 1H), 3.77 (d, J = 11.3 Hz, 6H). ^{13}C NMR(151 MHz, $CDCl_3$): δ (ppm) 150.3 (d, J = 7.8 Hz), 134.1 (d, J = 6.2 Hz), 132.4, 130.7, 130.2, 130.0, 126.6, 103.28 (d, J = 4.3 Hz). 54.8 (d, J = 6.3 Hz) (2). ^{31}P NMR (243 MHz, $CDCl_3$): δ (ppm) -4.7. HRMS (m/z) calcd for $C_{10}H_{13}ClO_4P[M+H]^+$ 263.02400, found 263.02275.

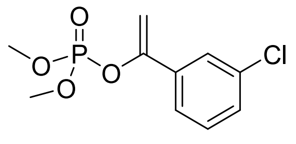

3i

1-(3-chlorophenyl)vinyl dimethyl phosphate (**3i**). Colorless oil, yield 94%; ^{1}H NMR(600 MHz, $CDCl_3$): δ (ppm) 7.54 (t, J = 1.7 Hz, 1H), 7.46 (dt, J = 7.3, 1.6 Hz, 1H), 7.34-7.27 (m, 2H), 5.33-5.29 (m, 1H), 5.26 (dd, J = 3.1, 2.1 Hz, 1H), 3.85 (d, J = 11.3 Hz, 6H). ^{13}C NMR(151 MHz, $CDCl_3$): δ (ppm) 150.9 (d, J = 7.9 Hz), 135.8 (d, J = 7.3 Hz), 134.5, 129.7, 129.1, 125.2, 123.3, 98.5 (d, J = 4.0 Hz), 54.9 (d, J = 6.3 Hz) (2). ^{31}P NMR (243 MHz, $CDCl_3$): δ (ppm) -4.1. HRMS (m/z) calcd for $C_{10}H_{13}ClO_4P[M+H]^+$ 263.02400, found 263.02279.

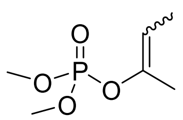

3j

dimethyl pent-2-en-3-yl phosphate (**3j, major**). Colorless oil, yield 70% (**major + minor**); ^{1}H NMR(600 MHz, $CDCl_3$): δ (ppm) 5.27-5.23 (m, 1H), 3.75 (d, J = 11.3 Hz, 6H), 1.86 (s, 3H), 1.57-1.54 (m, 3H). ^{13}C NMR(151 MHz, $CDCl_3$): δ (ppm) 145.1 (d, J = 8.9 Hz), 109.2 (d, J = 5.6 Hz), 54.5 (d, J = 6.3 Hz) (2), 15.6 (d, J = 3.5 Hz), 11.8 (d, J = 0.7 Hz). ^{31}P NMR (243 MHz, $CDCl_3$): δ (ppm) -3.7. HRMS (m/z) calcd for $C_6H_{14}O_4P[M+H]^+$ 181.06297, found 181.06192.

dimethyl pent-2-en-3-yl phosphate (**3j, minor**). Colorless oil, yield 70% (**major +

minor); ^{1}H NMR(600 MHz, $CDCl_3$): δ (ppm) 4.83 (q, J = 7.2 Hz, 1H), 3.78(d, J = 11.3 Hz, 6H), 1.93 (s, 3H), 1.58-1.56 (m, 3H). ^{13}C NMR(151 MHz, $CDCl_3$): δ (ppm) 144.6 (d, J = 8.4 Hz), 109.5 (d, J = 7.9 Hz), 54.5 (d, J = 6.3 Hz) (2), 20.2, 10.5 (d, J = 1.7 Hz). ^{31}P NMR (243 MHz, $CDCl_3$): δ (ppm) -3.9. HRMS (m/z) calcd for $C_6H_{14}O_4P[M+H]^+$ 181.06297, found 181.06192.

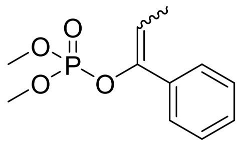

3k

dimethyl (1-phenylprop-1-en-1-yl) phosphate (**3k, major**). Colorless oil, yield 90% (**major + minor**); ^{1}H NMR(600 MHz, $CDCl_3$): δ (ppm) 7.49-7.47 (m, 2H), 7.33-7.30 (m, 2H), 7.27-7.25 (m, 1H), 5.65 (qd, J = 7.2, 3.0 Hz, 1H), 3.71 (d, J = 11.3 Hz, 6H), 1.87 (dd, J = 7.2, 3.0Hz, 3H). ^{13}C NMR(151 MHz, $CDCl_3$): δ (ppm) 146.6 (d, J = 9.1 Hz), 135.4 (d, J = 1.5 Hz),128.4, 128.2 (2), 125.1 (2), 112.2 (d, J = 6.5 Hz), 54.6 (d, J = 6.3 Hz) (2), 11.6 (d, J = 1.8 Hz). ^{31}P NMR (243 MHz, $CDCl_3$): δ (ppm) -3.3. HRMS (m/z) calcd for $C_{11}H_{16}O_4P[M+H]^+$ 243.07862, found 243.07732.

dimethyl (1-phenylprop-1-en-1-yl) phosphate (**3k, minor**). Colorless oil, yield 90% (**major + minor**); ^{1}H NMR(600 MHz, $CDCl_3$): δ (ppm) 7.45-7.43 (m, 2H), 7.37-7.35 (m, 2H), 7.33-7.30 (m, 1H), 5.73 (qd, J = 7.2, 3.0 Hz, 1H), 3.70 (d, J = 11.3 Hz, 6H), 1.74 (dd, J = 7.2, 3.0Hz, 3H). ^{13}C NMR(151 MHz, $CDCl_3$): δ (ppm) 146.2 (d, J = 9.1 Hz), 133.8 (d, J = 1.5 Hz), 128.5, 128.1 (2), 128.0 (2), 125.1, 111.8 (d, J = 6.5 Hz), 54.5 (d, J = 6.3 Hz) (2), 12.8 (d, J = 1.8 Hz). ^{31}P NMR (243 MHz, $CDCl_3$): δ (ppm) -3.4. HRMS (m/z) calcd for $C_{11}H_{16}O_4P[M+H]^+$ 243.07862, found 243.07732.

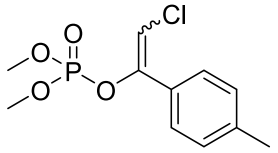

3l

2-chloro-1-(p-tolyl)vinyl dimethyl phosphate (**3l, major**). Colorless oil, yield 95% (**major + minor**); ^{1}H NMR(600 MHz, $CDCl_3$): δ (ppm) 7.38 (d, J = 8.2 Hz, 2H), 7.17 (d, J = 8.0 Hz, 2H), 6.14 (d, J = 2.2 Hz, 1H), 3.79 (d, J = 11.5 Hz, 6H), 2.35 (s, 3H). ^{13}C NMR(151 MHz, $CDCl_3$): δ (ppm) 148.3 (d, J = 8.4 Hz), 139.7, 130.2, 129.3 (2), 125.7 (2), 105.6 (d, J = 8.0 Hz), 55.0 (d, J = 6.0 Hz) (2), 21.3. ^{31}P NMR (243 MHz, $CDCl_3$): δ (ppm) -3.6. HRMS (m/z) calcd for $C_{11}H_{15}Cl\ O_4P[M+H]^+$ 277.03965, found 277.03848.

2-chloro-1-(p-tolyl)vinyl dimethyl phosphate (**3l, minor**). Colorless oil, yield 95%

(**major + minor**); ^{1}H NMR(600 MHz, $CDCl_3$): δ (ppm) 7.60 (d, J = 8.2 Hz, 2H), 7.21 (d, J = 8.0 Hz, 2H), 6.43 (d, J = 2.2 Hz, 1H), 3.75 (d, J = 11.5 Hz, 6H), 2.37 (s, 3H). ^{13}C NMR(151 MHz, $CDCl_3$): δ (ppm) 147.4 (d, J = 8.4 Hz), 139.8, 130.2, 128.8 (2), 128.2 (2), 107.9 (d, J = 8.0 Hz), 54.9 (d, J = 6.0 Hz) (2), 21.4. ^{31}P NMR (243 MHz, $CDCl_3$): δ (ppm) -3.5. HRMS (m/z) calcd for $C_{11}H_{15}Cl\ O_4P[M+H]^+$ 277.03965, found 277.03848.

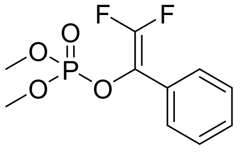

3m

2,2-difluoro-1-phenylvinyl dimethyl phosphate (**3m**). Colorless oil, yield 90%; ^{1}H NMR(600 MHz, $CDCl_3$): δ (ppm) 7.51 (d, J = 8.3 Hz, 2H), 7.41 (t, J = 7.7 Hz, 2H), 7.34 (t, J = 7.4 Hz, 1H), 3.77 (d, J = 11.4 Hz, 6H). ^{13}C NMR(151 MHz, $CDCl_3$): δ (ppm) 154.8 (ddd, J = 291.8, 287.9, 7.6 Hz), 134.2 (d, J = 243.3 Hz), 130.1, 128.9 (2), 128.6 (2), 126.3 (dd, J = 5.6, 3.5 Hz), 55.0 (d, J = 6.0 Hz) (2). ^{31}P NMR(243 MHz, $CDCl_3$): δ (ppm) -1.59 (dd, J = 10.0, 6.9 Hz). HRMS (m/z) calcd for $C_{10}H_{12}O_4F_2P$ $[M+H]^+$ 265.04413, found 265.04277.

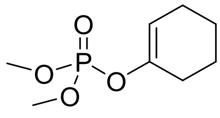

3n

cyclohex-1-en-1-yl dimethyl phosphate (**3n**). Colorless oil, yield 85%; ^{1}H NMR(600 MHz, $CDCl_3$): δ (ppm) 5.49-5.38 (m, 1H), 3.77 (d, J = 11.3 Hz, 6H), 2.24-2.11 (m, 2H), 2.11-1.99 (m, 2H), 1.69 (dtd, J = 9.1, 6.2, 3.1 Hz, 2H), 1.52 (dtd, J = 9.2, 6.1, 3.0 Hz, 2H). ^{13}C NMR(151 MHz, $CDCl_3$): δ (ppm) 147.5 (d, J = 9.0 Hz), 110.7 (d, J = 5.5 Hz), 54.5 (d, J = 6.4 Hz) (2), 27.5 (d, J = 4.2 Hz), 23.5 (d, J = 1.0 Hz), 22.6, 21.4. ^{31}P NMR(243 MHz, $CDCl_3$): δ (ppm) -4.0. HRMS (m/z) calcd for $C_8H_{16}O_5P[M+H]^+$ 207.07862, found 207.07862.

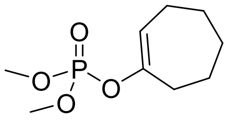

3o

cyclohept-1-en-1-yl dimethyl phosphate (**3o**). Colorless oil, yield 81%; ^{1}H NMR(600 MHz, $CDCl_3$): δ (ppm) 5.60 (td, J = 6.5, 2.5 Hz, 1H), 3.77 (d, J = 11.3 Hz, 6H), 2.45-2.34 (m, 2H), 2.04 (dt, J = 6.2, 5.3 Hz, 2H), 1.67 (dt, J = 11.3, 5.2 Hz, 2H), 1.61 (dt, J

= 8.3, 5.7 Hz, 2H), 1.55 (dt, J = 8.0, 5.9 Hz, 2H). ^{13}C NMR(151 MHz, $CDCl_3$): δ (ppm) 13C NMR (151 MHz, CDCl3) δ 152.0 (d, J = 9.5 Hz), 115.4 (d, J = 5.4 Hz), 54.5 (d, J = 6.3 Hz) (2), 33.2 (d, J = 3.7 Hz), 30.5, 26.8 (d, J = 0.8 Hz), 25.0, 24.7 (d, J = 0.8 Hz). ^{31}P NMR(243 MHz, $CDCl_3$): δ (ppm) -3.8. HRMS (m/z) calcd for $C_9H_{18}O_5P[M+H]^+$ 221.09427, found 221.09304.

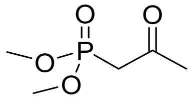

4a

dimethyl (2-oxopropyl)phosphonate (**4a**). Colorless oil, yield 85%; ^1H NMR(600 MHz, $CDCl_3$): δ (ppm) 3.76 (d, J = 11.2 Hz, 6H), 3.08 (d, J = 22.8 Hz, 2H), 2.30 (s, 3H). ^{13}C NMR(151 MHz, $CDCl_3$): δ (ppm) 199.7 (d, J = 6.2 Hz), 53.0 (d, J = 6.5 Hz) (2), 42.1 (d, J = 127.8 Hz), 31.4. ^{31}P NMR(243 MHz, $CDCl_3$): δ (ppm) 22.4. HRMS (m/z) calcd for $C_5H_{12}O_4P[M+H]^+$ 167.04732, found 167.04636.

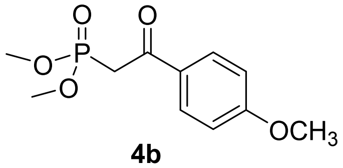

4b

dimethyl (2-(4-methoxyphenyl)-2-oxoethyl)phosphonate (**4b**). Colorless oil, yield 90%; ^1H NMR(600 MHz, $CDCl_3$): δ (ppm) 7.94 (dd, J = 8.8, 1.0 Hz, 2H), 7.00-6.82 (m, 2H), 3.83 (d, J = 1.2 Hz, 3H), 3.73 (d, J = 11.3 Hz, 6H), 3.57 (s, 1H), 3.53 (s, 1H). ^{13}C NMR(151 MHz, $CDCl_3$): δ (ppm) 190.0 (d, J = 6.4 Hz), 164.0, 131.3 (2), 129.3 (d, J = 2.2 Hz), 113.8 (2), 55.4, 53.0 (d, J = 6.5 Hz) (2), 37.1 (d, J = 131.0 Hz). ^{31}P NMR(243 MHz, $CDCl_3$): δ (ppm) 23.3. HRMS (m/z) calcd for $C_{11}H_{16}O_5P[M+H]^+$ 259.07354, found 259.07238.

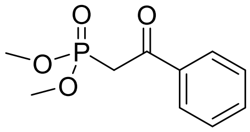

4c

dimethyl (2-oxo-2-phenylethyl)phosphonate (**4c**). Colorless oil, yield 85%; ^1H NMR(600 MHz, $CDCl_3$): δ (ppm) 8.00-7.97 (m, 2H), 7.60-7.56 (m, 1H), 7.49-7.45 (m, 2H), 3.77 (d, J = 11.3 Hz, 6H), 3.65 (s, 1H), 3.61 (s, 1H). ^{13}C NMR(151 MHz, $CDCl_3$): δ (ppm) 191.7 (d, J = 6.6 Hz), 136.3 (d, J = 2.5 Hz), 133.8, 128.9 (2), 128.7 (2), 53.1 (d, J = 6.5 Hz) (2), 37.4 (d, J = 131.5 Hz). ^{31}P NMR(243 MHz, $CDCl_3$): δ (ppm) 22.8. HRMS (m/z) calcd for $C_{10}H_{14}O_4P[M+H]^+$ 229.06297, found 229.06176.

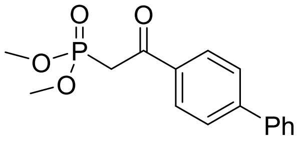

4d

1-([1,1'-biphenyl]-4-yl)vinyl dimethyl phosphate (**4d**). Colorless oil, yield 83%; ^{1}H NMR(600 MHz, $CDCl_3$): δ (ppm) 8.01 (d, J = 8.5 Hz, 2H), 7.64 (d, J = 8.4 Hz, 2H), 7.56 (dd, J = 8.2, 1.1 Hz, 2H), 7.40 (t, J = 7.6 Hz, 2H), 7.34 (d, J = 7.4 Hz, 1H), 3.73 (d, J = 11.3 Hz, 6H), 3.62 (s, 1H), 3.59 (s, 1H). ^{13}C NMR(151 MHz, $CDCl_3$): δ (ppm) 191.2 (d, J = 6.6 Hz), 146.5, 139.6, 135.0, 129.6 (2), 129.0 (2), 128.4, 127.3 (2), 127.2 (2), 53.2 (d, J = 6.5 Hz) (2), 37.5 (d, J = 131.0 Hz). ^{31}P NMR(243 MHz, $CDCl_3$): δ (ppm) 22.9. HRMS (m/z) calcd for $C_{16}H_{18}O_4P[M+H]^+$ 305.09427, found 305.09279.

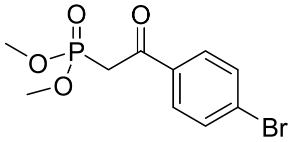

4e

dimethyl (2-(4-bromophenyl)-2-oxoethyl)phosphonate (**4e**). Colorless oil, yield 85%; ^{1}H NMR(600 MHz, $CDCl_3$): δ (ppm) 8.11-7.76 (m, 2H), 7.70-7.53 (m, 2H), 3.74 (d, J = 11.3 Hz, 6H), 3.59 (s, 1H), 3.55 (s, 1H). ^{13}C NMR(151 MHz, $CDCl_3$): δ (ppm) 190.7 (d, J = 6.6 Hz), 134.9 (d, J = 2.2 Hz), 131.9 (2), 130.4 (2), 129.1, 53.1 (d, J = 6.6 Hz) (2), 37.5 (d, J = 130.7 Hz). ^{31}P NMR(243 MHz, $CDCl_3$): δ (ppm) 22.2. HRMS (m/z) calcd for $C_{10}H_{13}BrO_4P[M+H]^+$ 306.97348, found 306.97215.

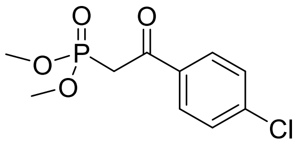

4f

dimethyl (2-(4-chlorophenyl)-2-oxoethyl)phosphonate (**4f**). Colorless oil, yield 85%; ^{1}H NMR(600 MHz, $CDCl_3$): δ (ppm) 8.03-7.74 (m, 2H), 7.64-7.31 (m, 2H), 3.72 (d, J = 11.3 Hz, 6H), 3.56 (s, 1H), 3.52 (s, 1H). ^{13}C NMR(151 MHz, $CDCl_3$): δ (ppm) 190.5 (d, J = 6.6 Hz), 140.4, 134.6 (d, J = 2.2 Hz), 130.4 (2), 129.0 (2), 53.21 (d, J = 6.5 Hz) (2), 37.6 (d, J = 130.8 Hz). ^{31}P NMR(243 MHz, $CDCl_3$): δ (ppm) 22.3. HRMS (m/z) calcd for $C_{10}H_{13}ClO_4P[M+H]^+$ 263.02400, found 263.02286.

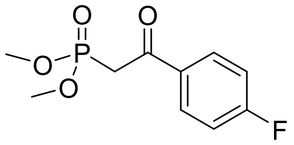

4g

dimethyl (2-(4-fluorophenyl)-2-oxoethyl)phosphonate (**4g**). Colorless oil, yield 93%; ^{1}H NMR (600 MHz, $CDCl_3$): δ (ppm) 8.01 (dd, J = 8.8, 5.3 Hz, 2H), 7.11 (t, J = 8.6 Hz, 2H), 3.74 (d, J = 11.3 Hz, 6H), 3.60 (s, 1H), 3.56 (s, 1H). ^{13}C NMR (151 MHz, $CDCl_3$): δ (ppm) 190.1 (d, J = 6.5 Hz), 166.0 (d, J = 256.3 Hz), 132.7 (t, J = 2.5 Hz), 131.7 (d, J = 9.6 Hz) (2), 115.8 (d, J = 22.0 Hz) (2), 53.1 (d, J = 6.6 Hz) (2), 37.46 (d, J = 130.8 Hz). ^{31}P NMR (243 MHz, $CDCl_3$): δ (ppm) 22.5. HRMS (m/z) calcd for $C_{10}H_{13}FO_4P[M+H]^+$ 247.05355, found 247.05225.

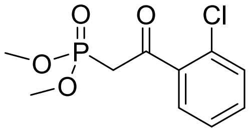

4h

dimethyl (2-(2-chlorophenyl)-2-oxoethyl)phosphonate (**4h**). Colorless oil, yield 67%; ^{1}H NMR(600 MHz, $CDCl_3$): δ (ppm) 7.59 (dd, J = 4.6, 3.7 Hz, 1H), 7.42 (dd, J = 4.9, 1.3 Hz, 2H), 7.38-7.32 (m, 1H), 3.76 (d, J = 11.3 Hz, 6H), 3.74 (s, 1H), 3.70 (s, 1H). ^{13}C NMR(151 MHz, $CDCl_3$): δ (ppm) 194.2 (d, J = 7.0 Hz), 138.4 (d, J = 2.3 Hz), 132.4, 131.1, 130.6, 129.9, 127.0, 53.1 (d, J = 6.5 Hz), 41.1 (d, J = 130.2 Hz). ^{31}P NMR (243 MHz, $CDCl_3$): δ (ppm) 22.0. HRMS (m/z) calcd for $C_{10}H_{13}ClO_4P[M+H]^+$ 263.02400, found 263.02278.

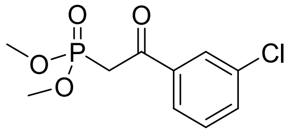

4i

dimethyl (2-(3-chlorophenyl)-2-oxoethyl)phosphonate (**4i**). Colorless oil, yield 70%; ^{1}H NMR (600 MHz, $CDCl_3$): δ (ppm) 7.94 (t, *J* = 1.9 Hz, 1H), 7.88-7.82 (m, 1H), 7.53 (ddd, *J* = 8.0, 2.1, 0.9 Hz, 1H), 7.41 (t, J = 7.9 Hz, 1H), 3.76 (d, J = 11.3 Hz, 6H), 3.61 (s, 1H), 3.57 (s, 1H). ^{13}C NMR (151 MHz, $CDCl_3$): δ (ppm) 13C NMR (151 MHz, CDCl3) δ 190.5 (d, J = 6.7 Hz), 137.7 (d, J = 2.3 Hz), 135.0, 133.6, 130.0, 128.8, 127.1, 53.2 (d, J = 6.6 Hz) (2), 37.53 (d, J = 131.2 Hz). ^{31}P NMR (243 MHz, $CDCl_3$): δ (ppm) 22.1. HRMS (m/z) calcd for $C_{10}H_{13}ClO_4P[M+H]^+$ 263.02400, found 263.02268.

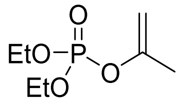

5a

diethyl prop-1-en-2-yl phosphate (**5a**). Colorless oil, yield 77%; ^{1}H NMR (600 MHz, $CDCl_3$): δ (ppm) 4.72 (t, J = 1.8 Hz, 1H), 4.45 (d, J = 0.7 Hz, 1H), 4.21-4.06 (m, 4H),

1.90 (s, 3H), 1.31 (td, J = 7.1, 0.7 Hz, 6H). ^{13}C NMR (151 MHz, $CDCl_3$): δ (ppm) 152.0 (d, J = 8.5 Hz), 97.9 (d, J = 4.9 Hz), 64.1 (d, J = 6.1 Hz) (2), 20.6 (d, J = 5.1 Hz), 15.9 (d, J = 6.8 Hz) (2). ^{31}P NMR (243 MHz, $CDCl_3$): δ (ppm) -6.8. HRMS (m/z) calcd for $C_7H_{16}O_4P[M+H]^+$ 195.07862, found 195.07755.

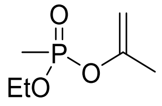

5b

ethyl prop-1-en-2-yl methylphosphonate (**5b**). Colorless oil, yield 85%; ^1H NMR (600 MHz, $CDCl_3$): δ (ppm) 4.68 (t, J = 1.9 Hz, 1H), 4.46-4.41 (m, 1H), 4.20-4.00 (m, 2H), 1.88 (s, 3H), 1.51 (d, J = 17.6 Hz, 3H), 1.29 (t, J = 7.1 Hz, 3H). ^{13}C NMR (151 MHz, $CDCl_3$): δ (ppm) 152.0 (d, J = 9.2 Hz), 98.0 (d, J = 5.0 Hz), 61.91 (d, J = 6.5 Hz), 21.14 (d, J = 3.9 Hz), 16.17 (d, J = 6.3 Hz), 11.1 (d, J = 145.0 Hz), 10.6. ^{31}P NMR (243 MHz, $CDCl_3$): δ (ppm) 26.4. HRMS (m/z) calcd for $C_6H_{14}O_3P[M+H]^+$ 165.06806, found 165.06712.

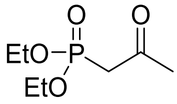

6a

diethyl (2-oxopropyl)phosphonate (**6a**). Colorless oil, yield 86%; ^1H NMR (600 MHz, $CDCl_3$): δ (ppm) 4.31-4.01 (m, 4H), 3.04 (d, J = 22.9 Hz, 2H), 2.27 (s, 3H), 1.29 (t, J = 7.1 Hz, 6H). ^{13}C NMR (151 MHz, $CDCl_3$): δ (ppm) 199.8 (d, J = 6.1 Hz), 62.5 (d, J = 6.5 Hz) (2), 31.3, 16.17 (d, J = 6.3 Hz) (2). ^{31}P NMR (243 MHz, $CDCl_3$): δ (ppm) 19.7. HRMS (m/z) calcd for $C_7H_{16}O_4P[M+H]^+$ 195.07862, found 195.07751.

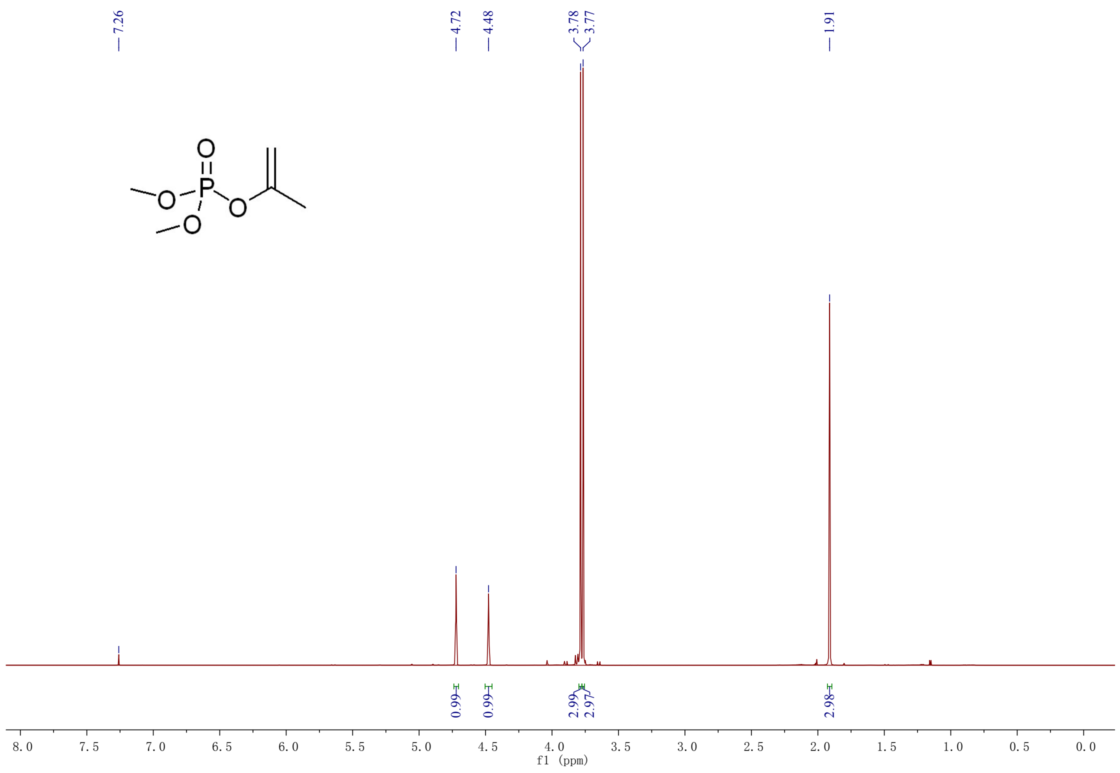

Fig.S 1 ^{1}H NMR of compound 3**a**

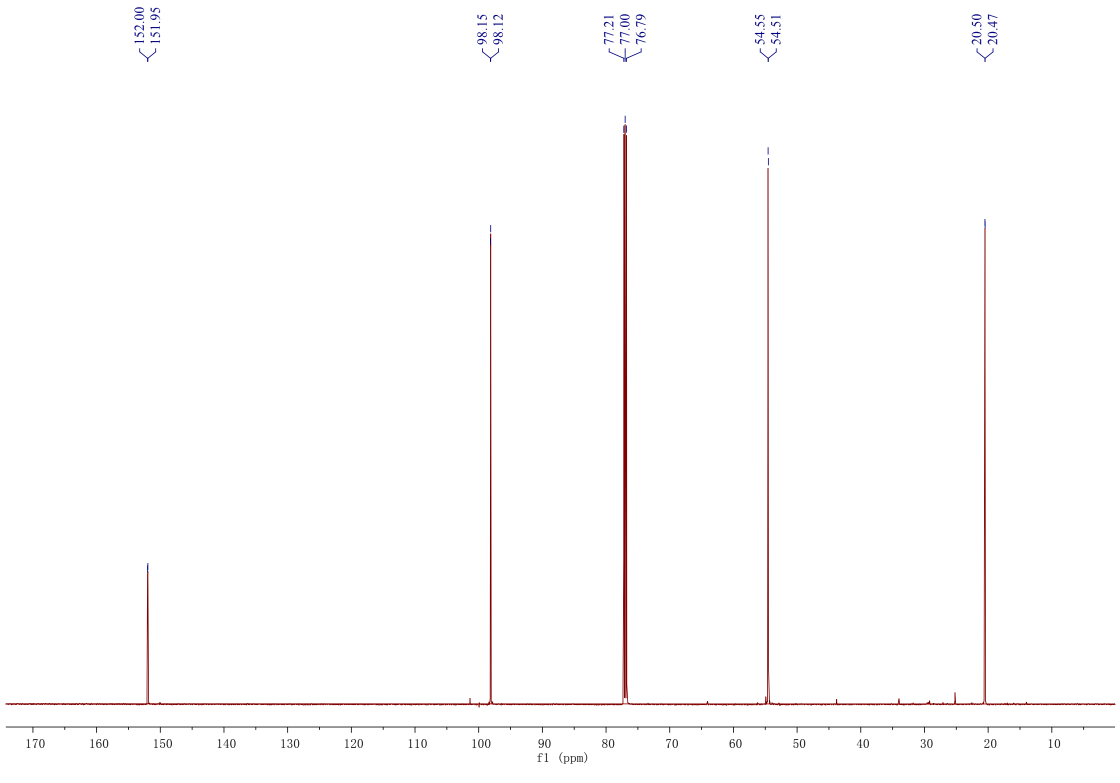

Fig.S 2 ^{13}C NMR of compound **3a**

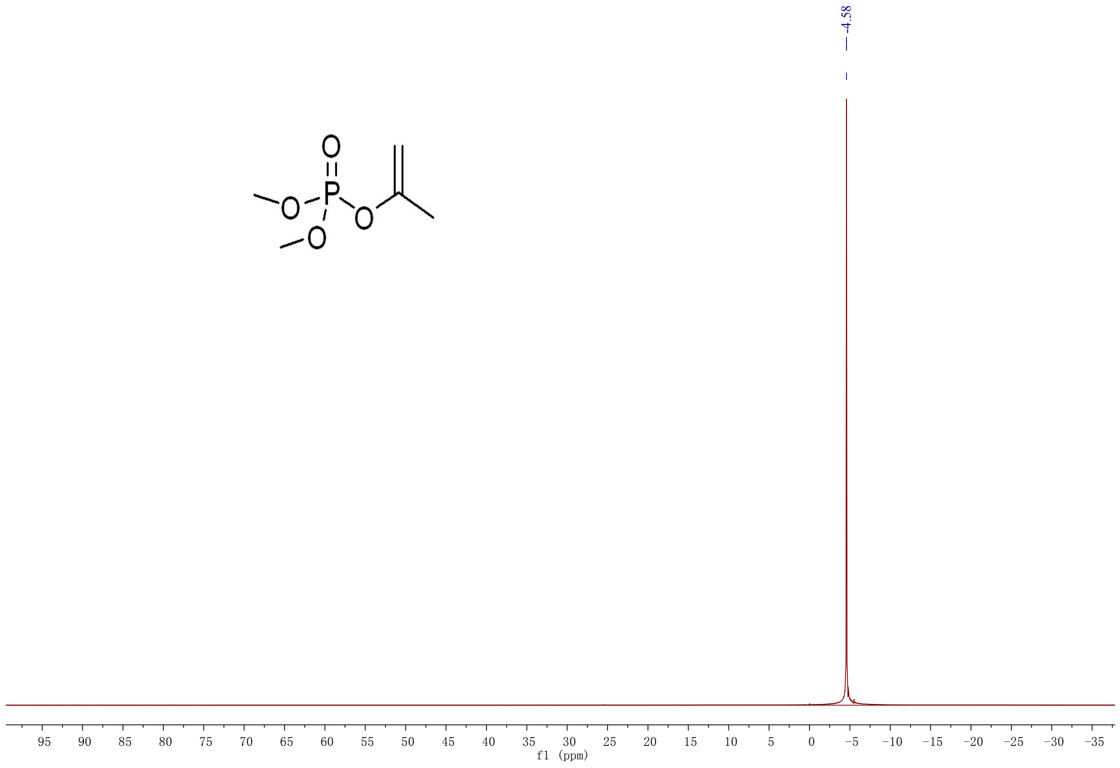

Fig.S 3 ^{31}P NMR of compound **3a**

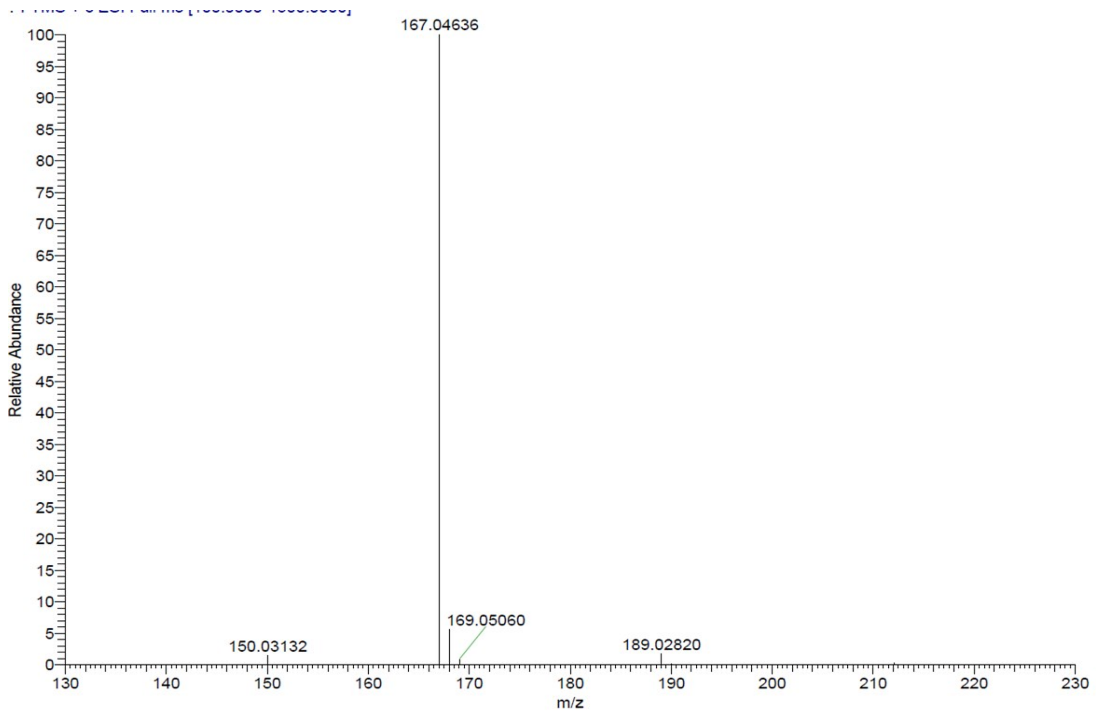

Fig.S 4 HRMS of compound **3a**

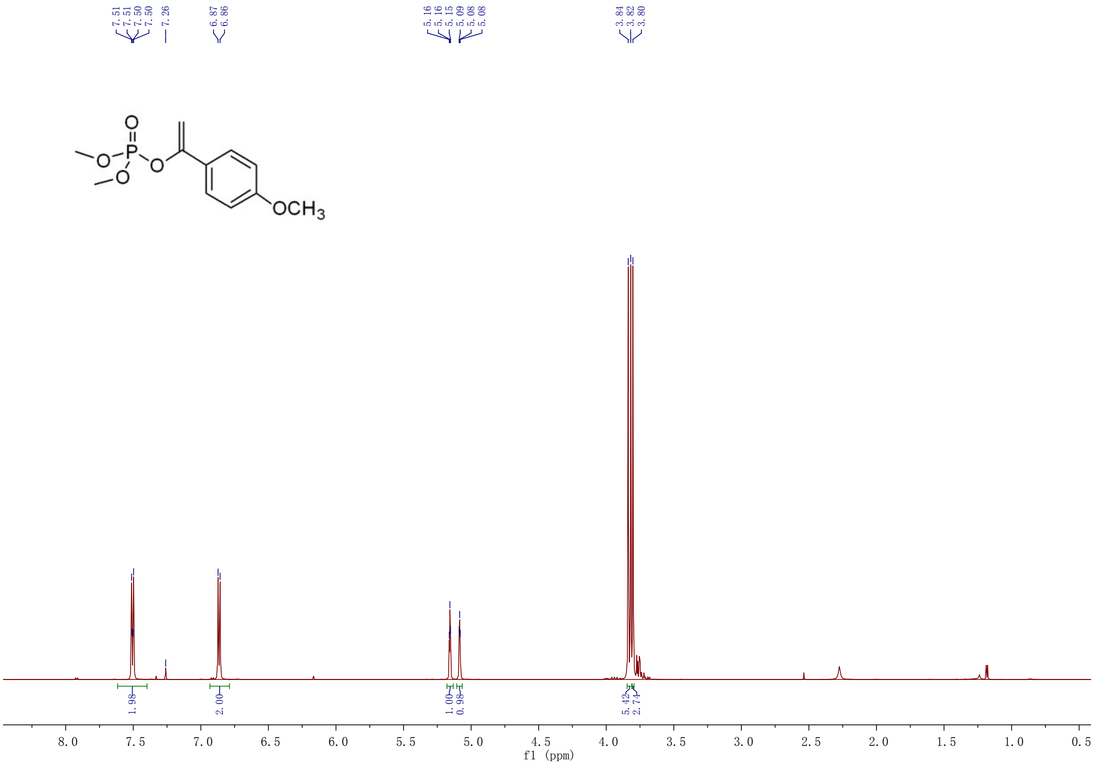

Fig.S 5 ^{1}H NMR of compound **3b**

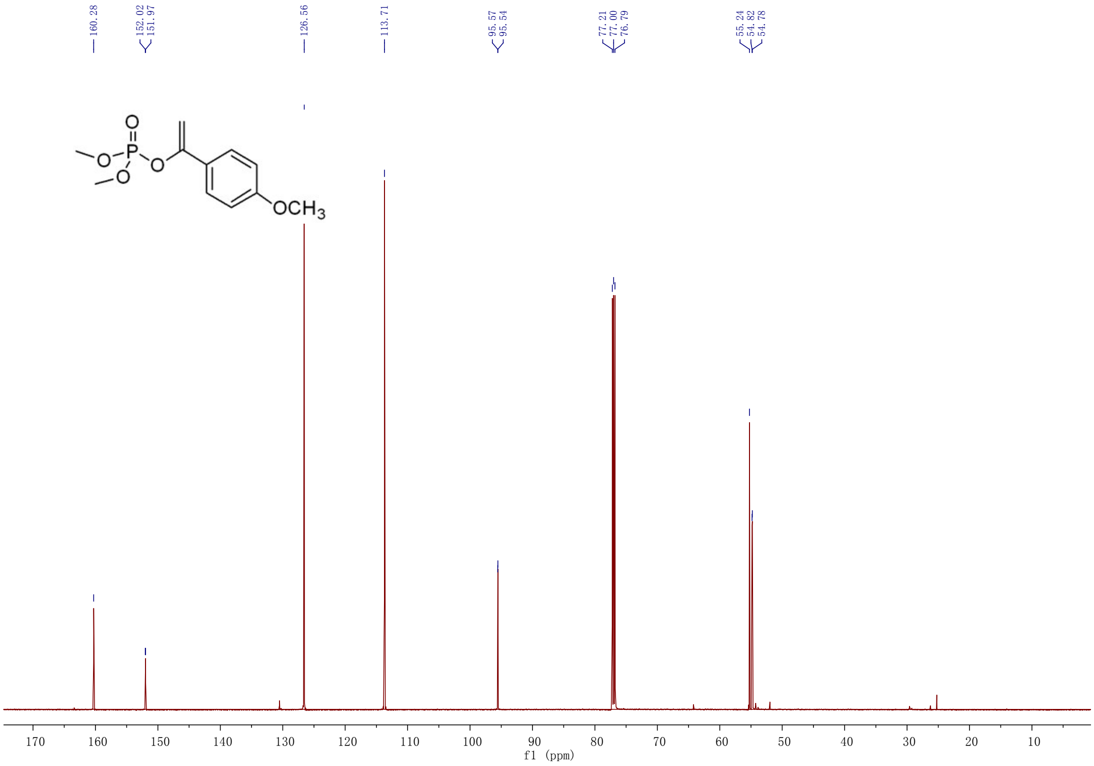

Fig.S 6 ^{13}C NMR of compound **3b**

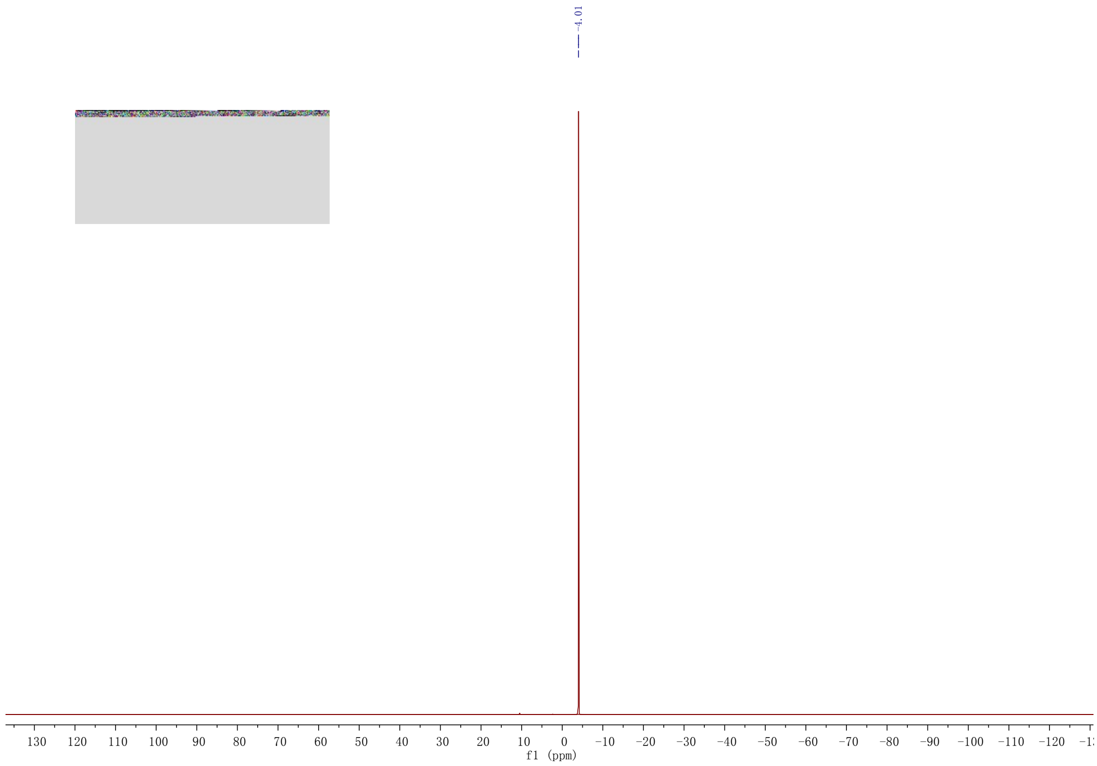

Fig.S 7 ^{31}P NMR of compound **3b**

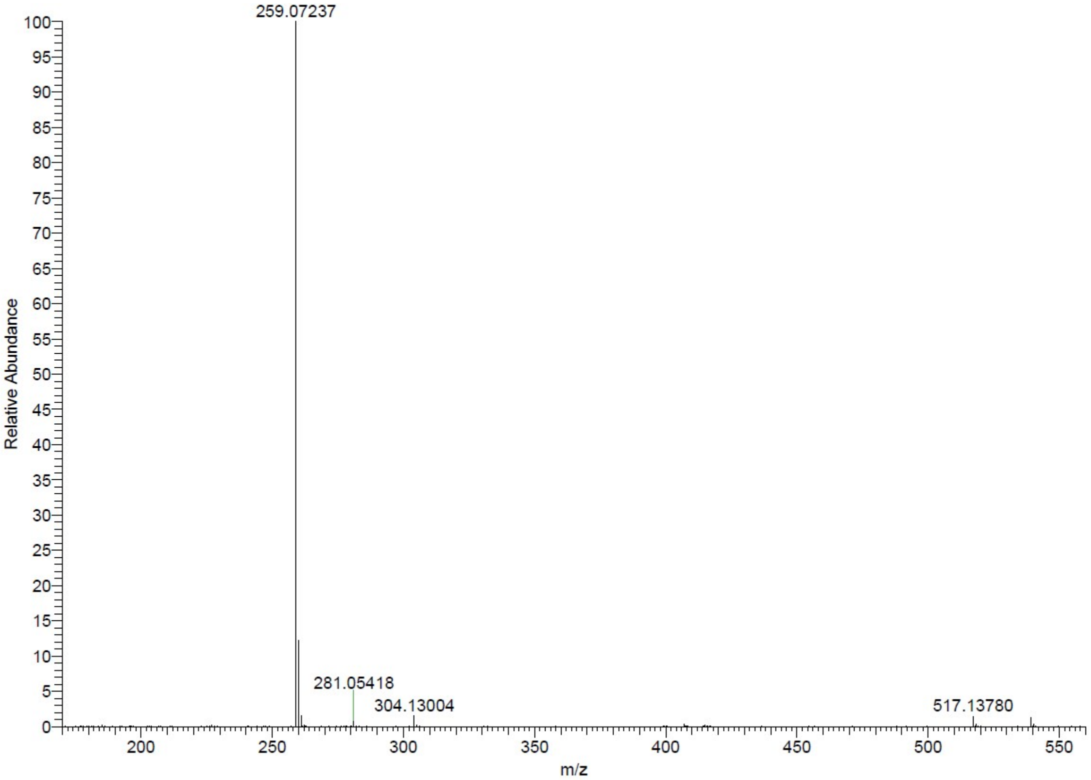

Fig.S 8 HRMS of compound **3b**

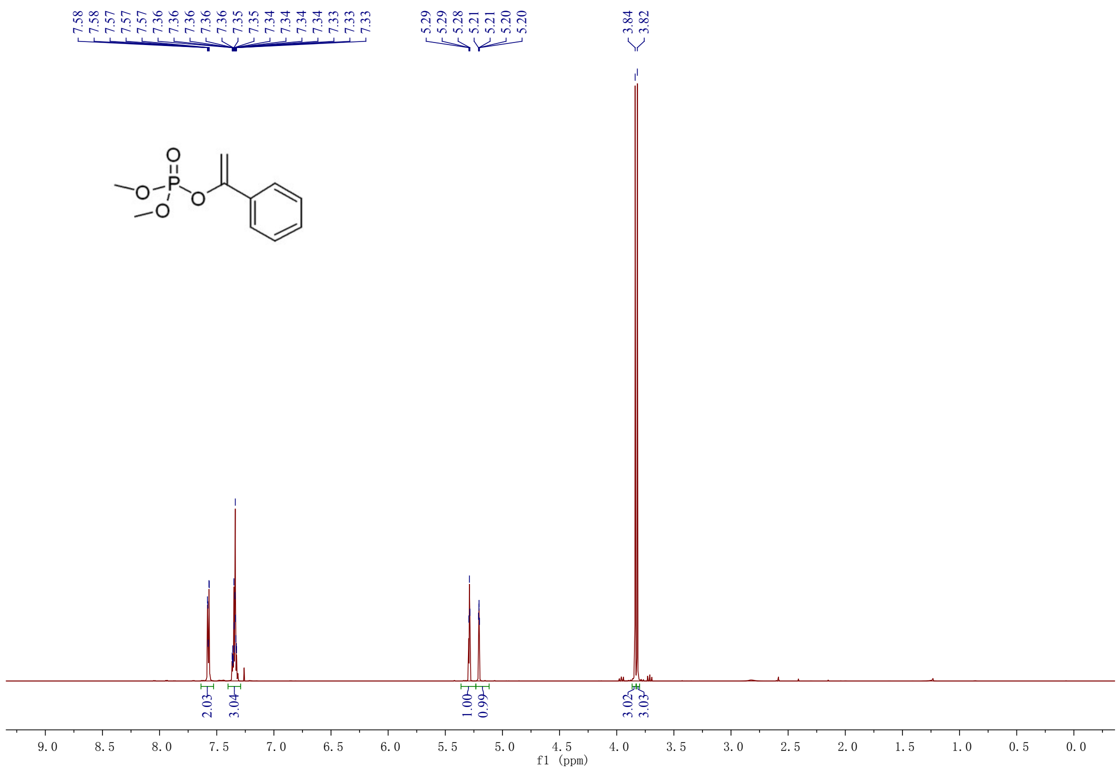

Fig.S 9 ^{1}H NMR of compound 3**c**

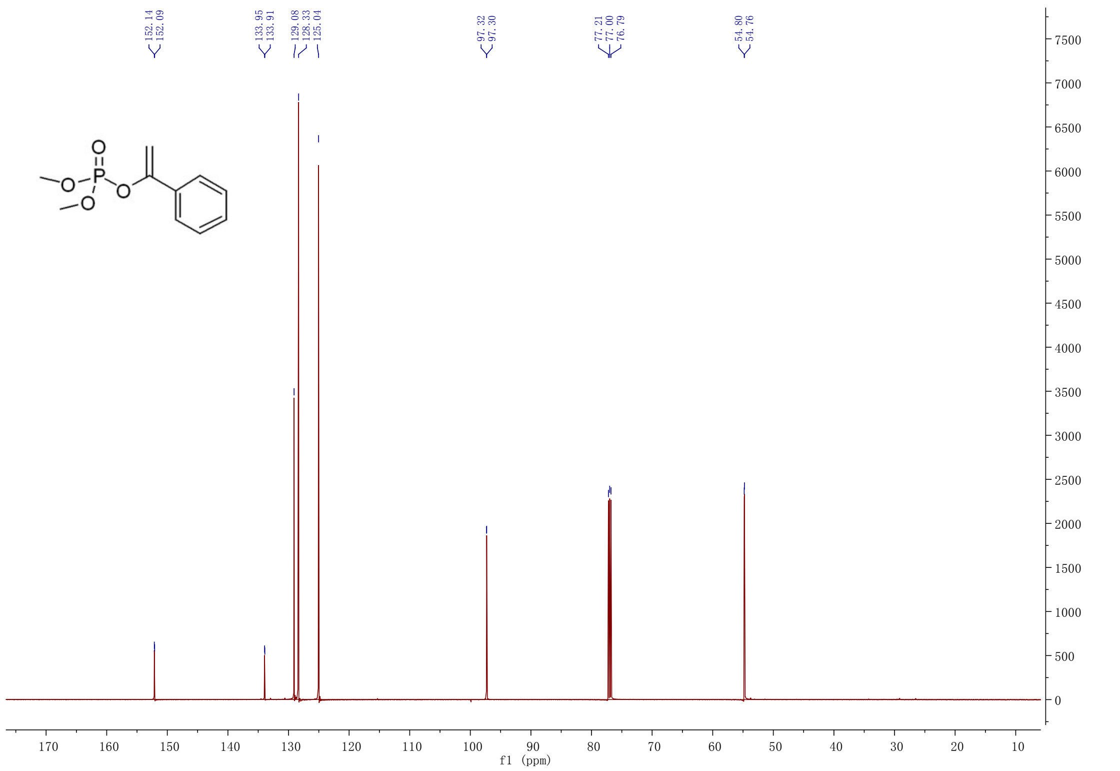

Fig.S 10 ^{13}C NMR of compound **3c**

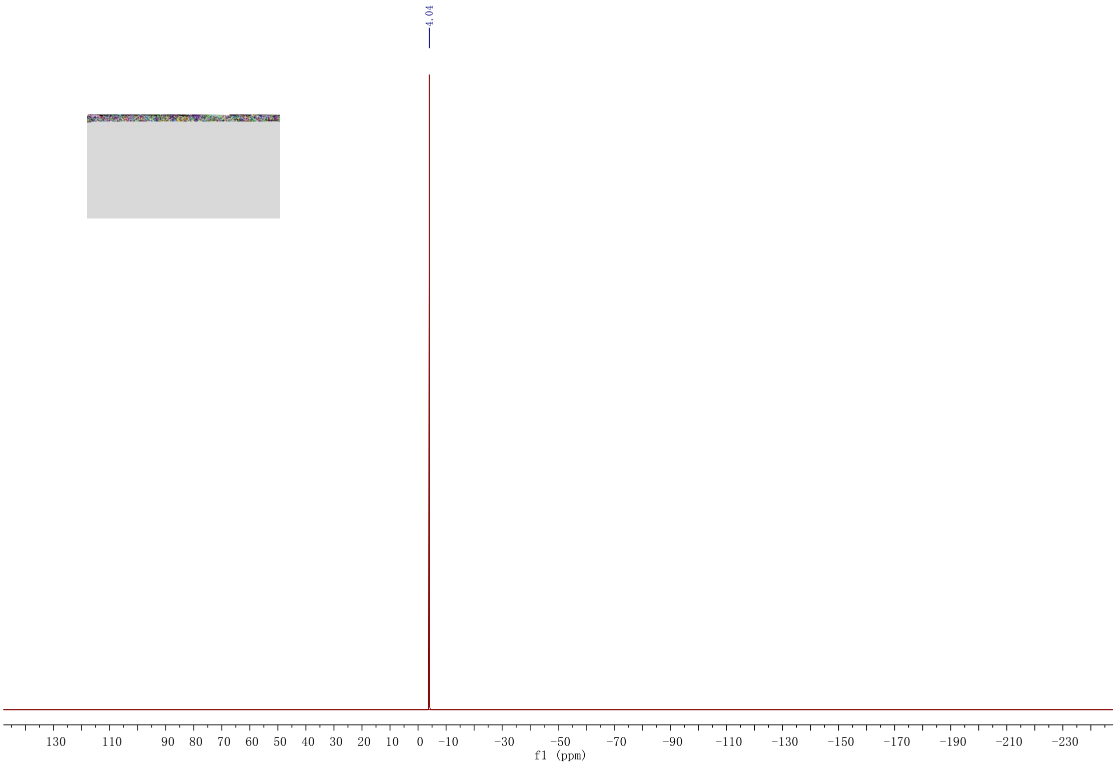

Fig.S 11 ^{31}P NMR of compound **3c**

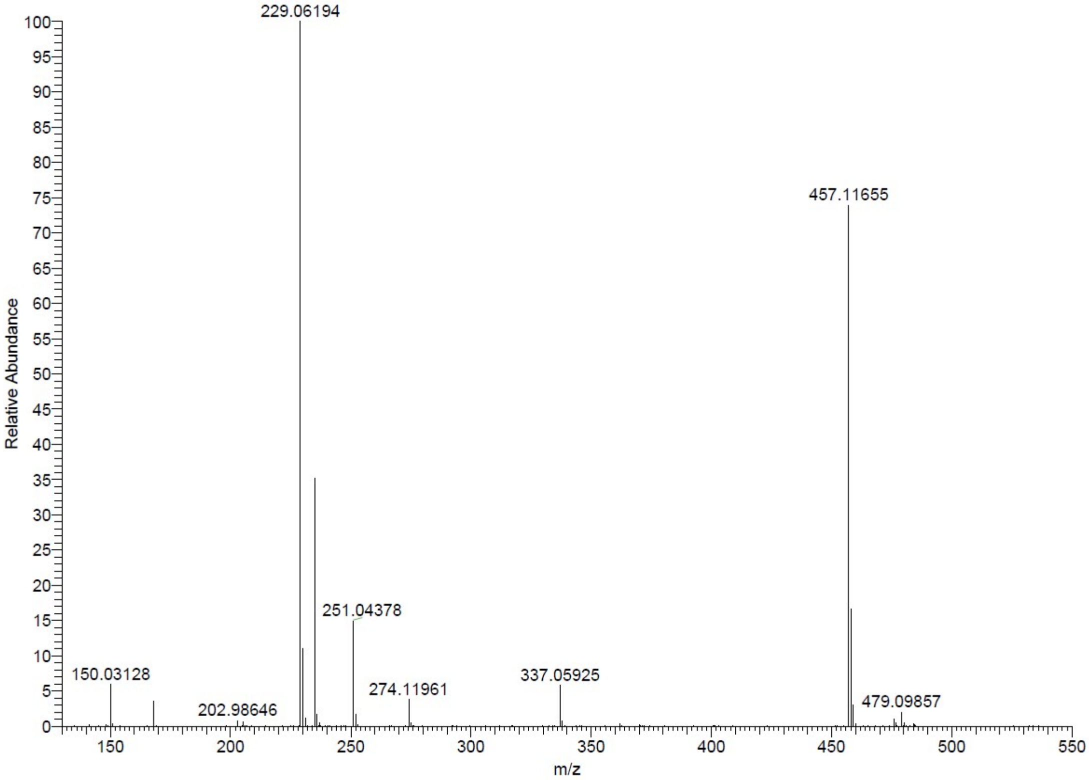

Fig.S 12 HRMS of compound **3c**

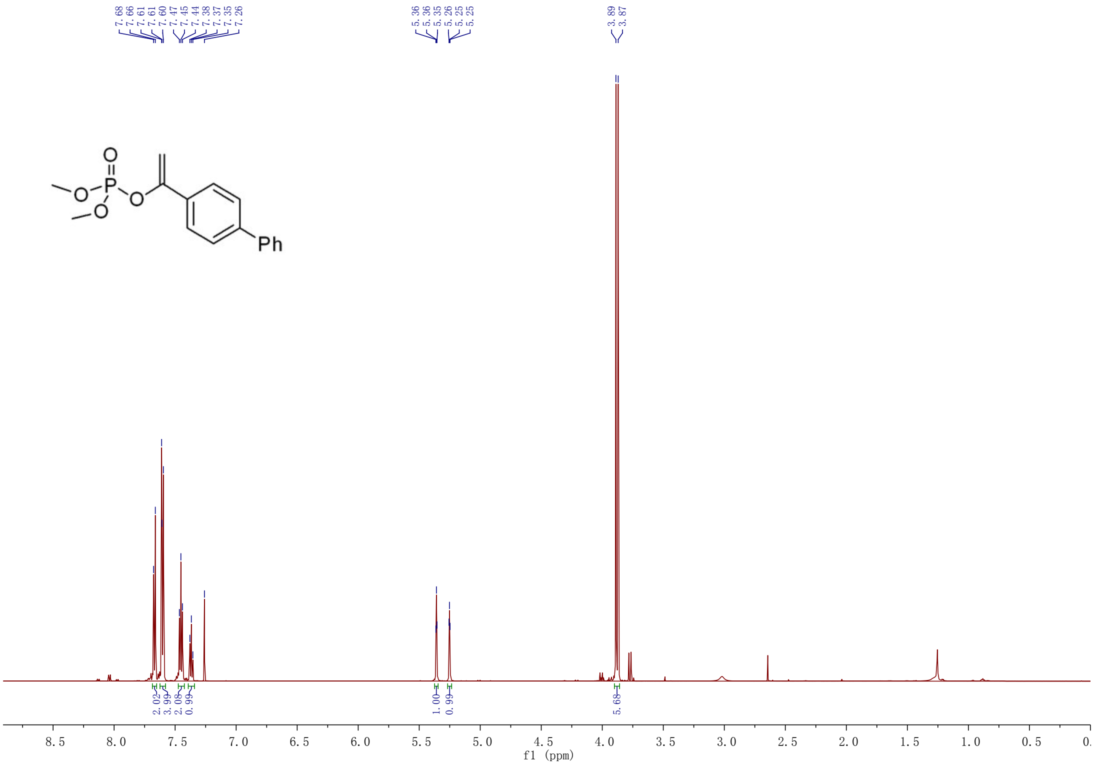

Fig.S 13 ^{1}H NMR of compound **3d**

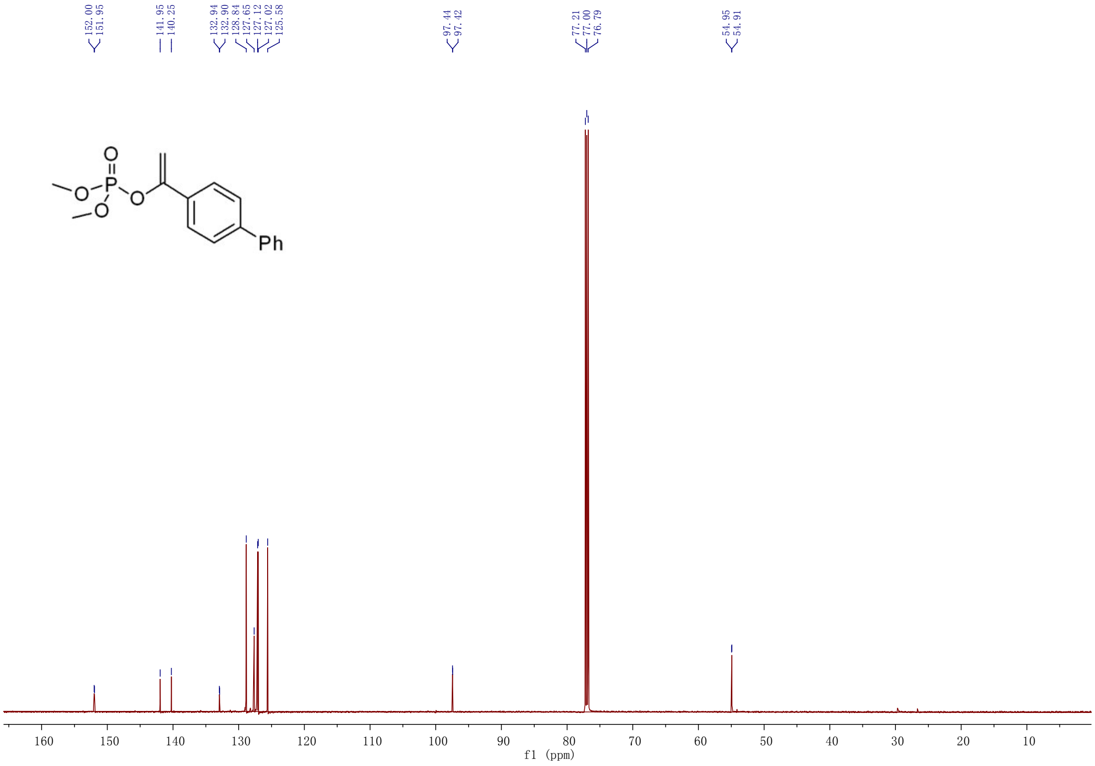

Fig.S 14 ^{13}C NMR of compound **3d**

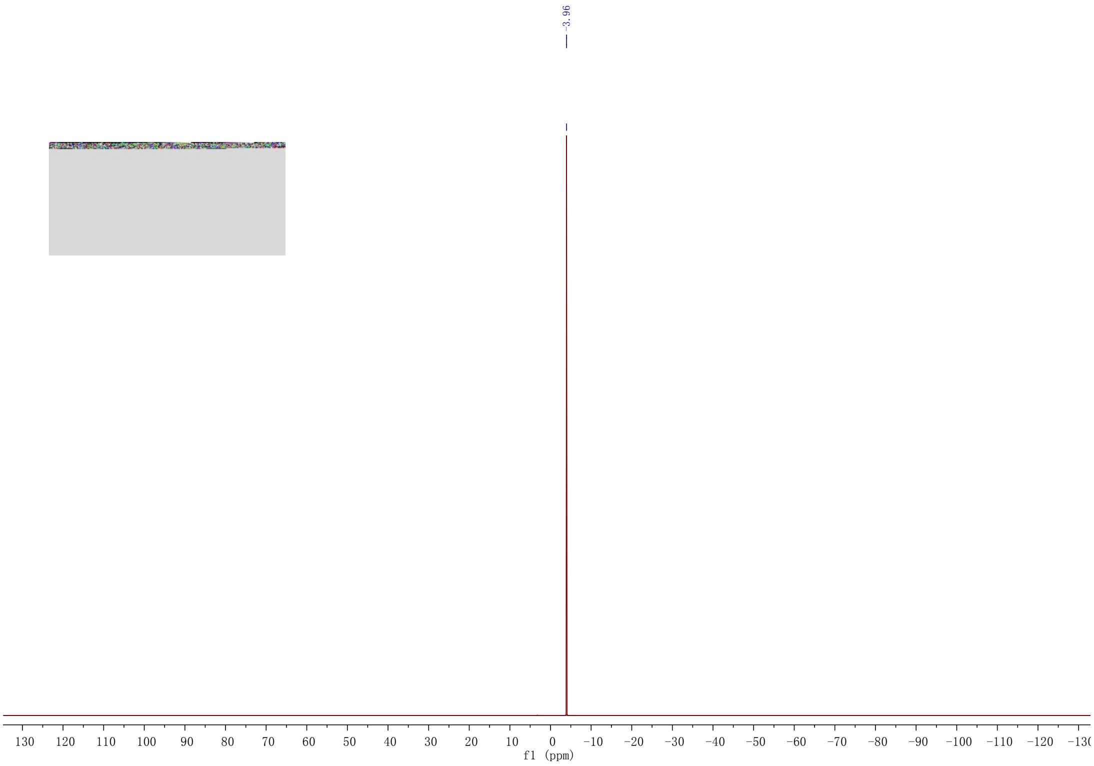

Fig.S 15 ^{31}P NMR of compound **3d**

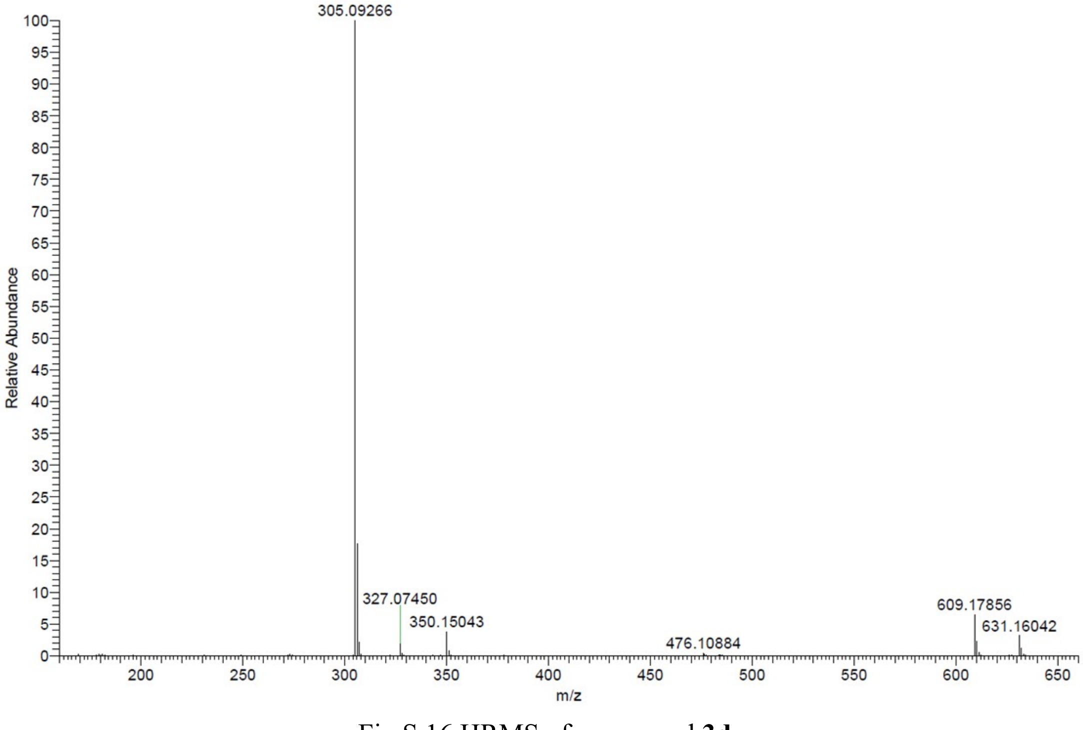

Fig.S 16 HRMS of compound **3d**

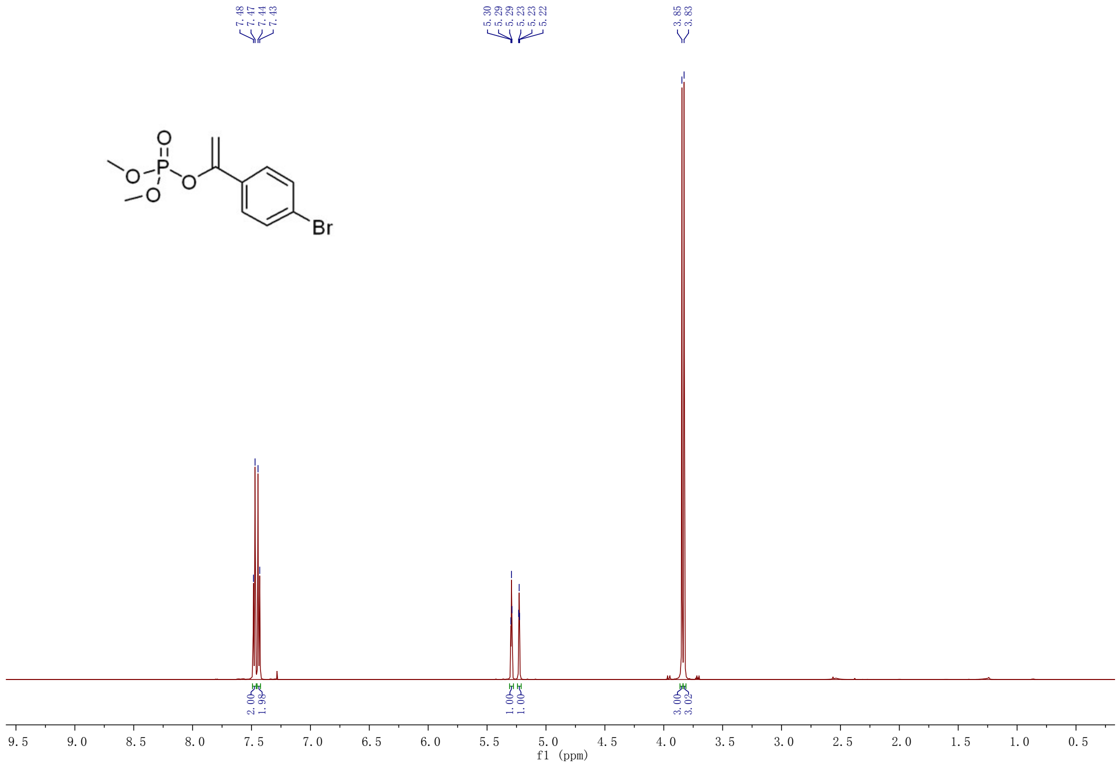

Fig.S 17 ^{1}H NMR of compound 3**e**

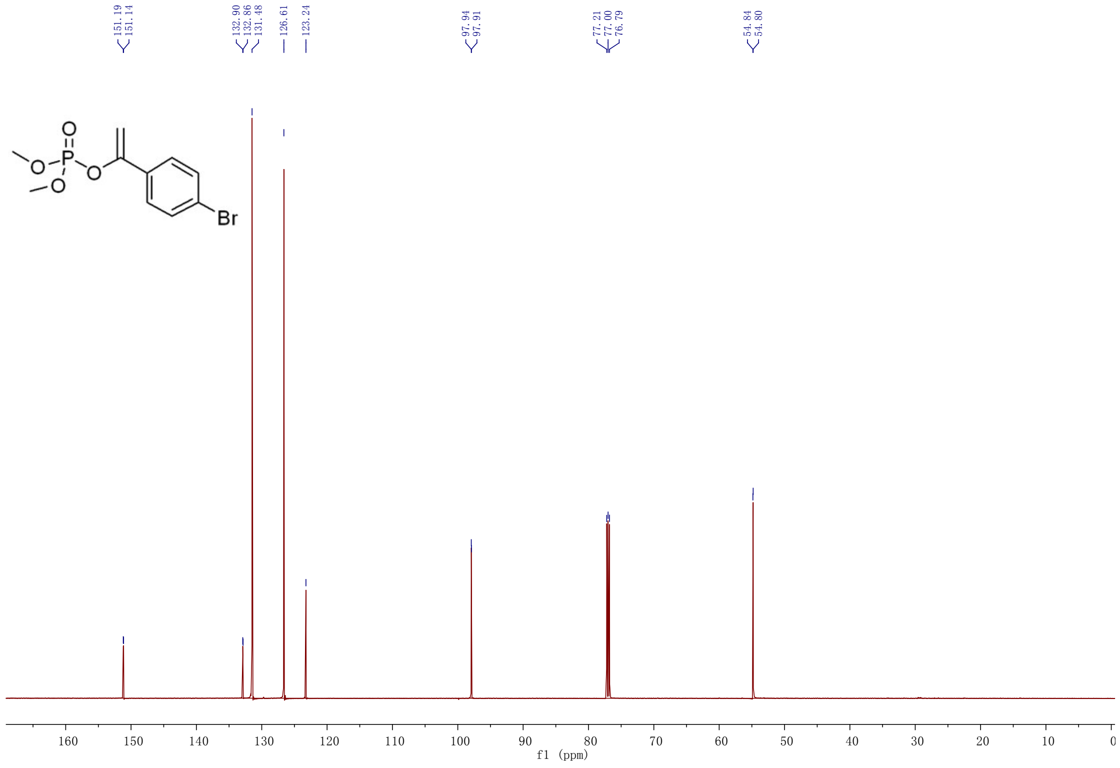

Fig.S 18 ^{13}C NMR of compound **3e**

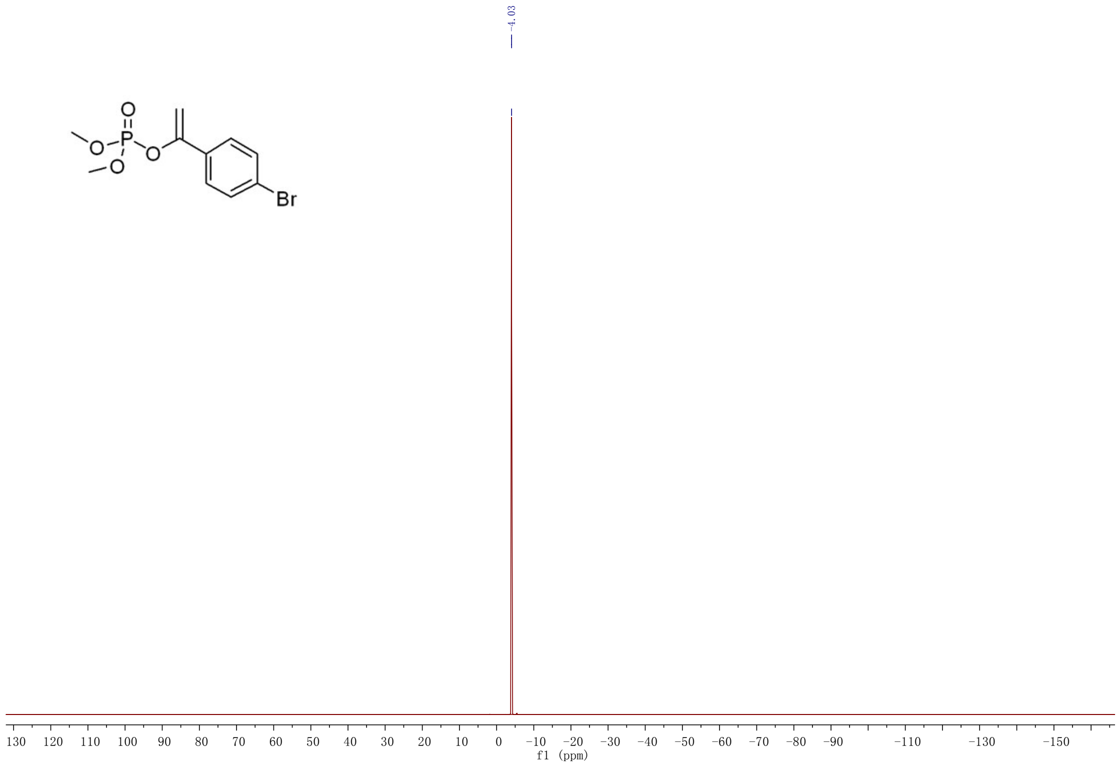

Fig.S 19 ^{31}P NMR of compound **3e**

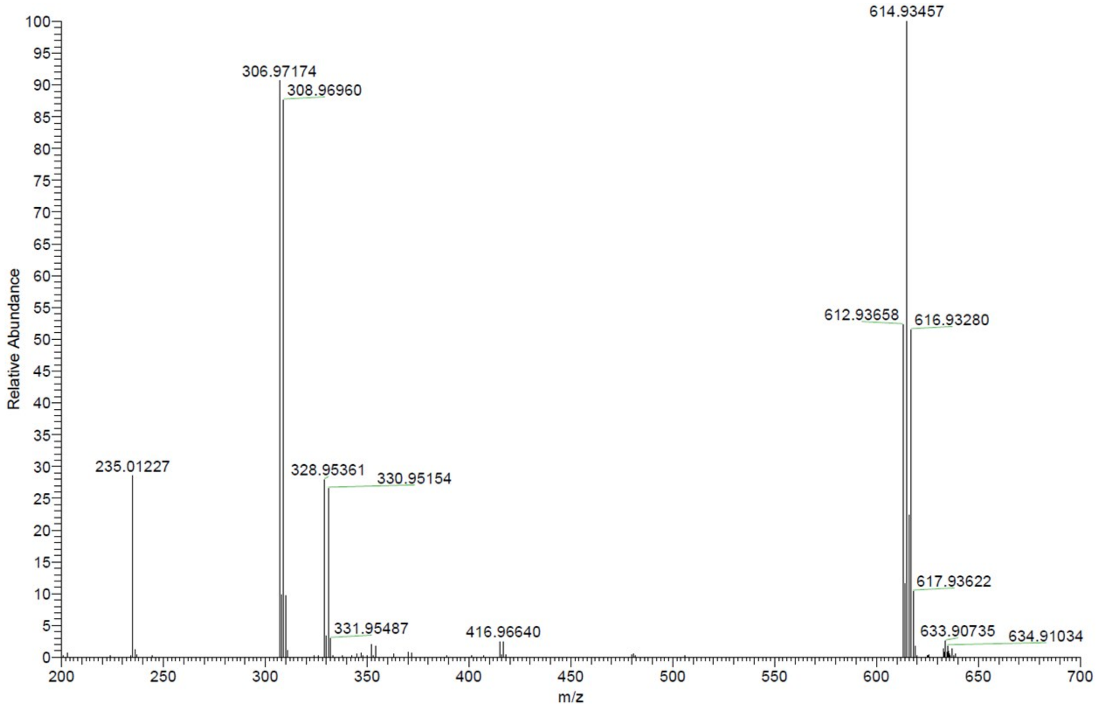

Fig.S 20 HRMS of compound **3e**

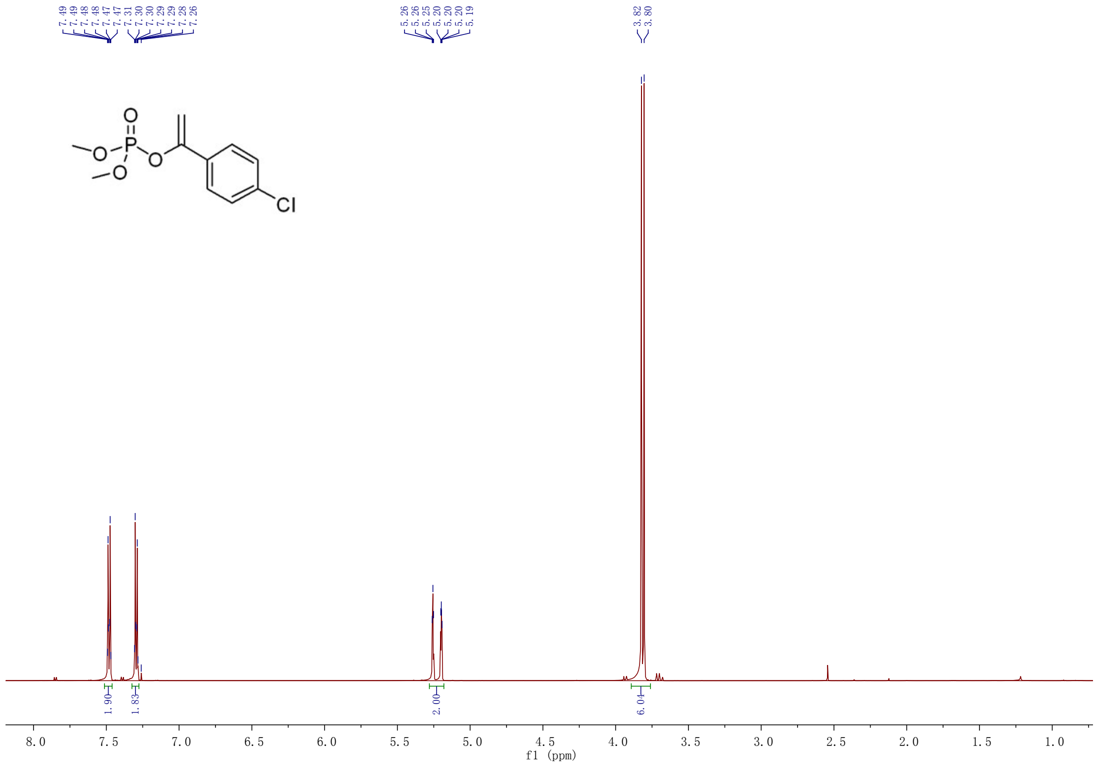

Fig.S 21 ^{1}H NMR of compound **3f**

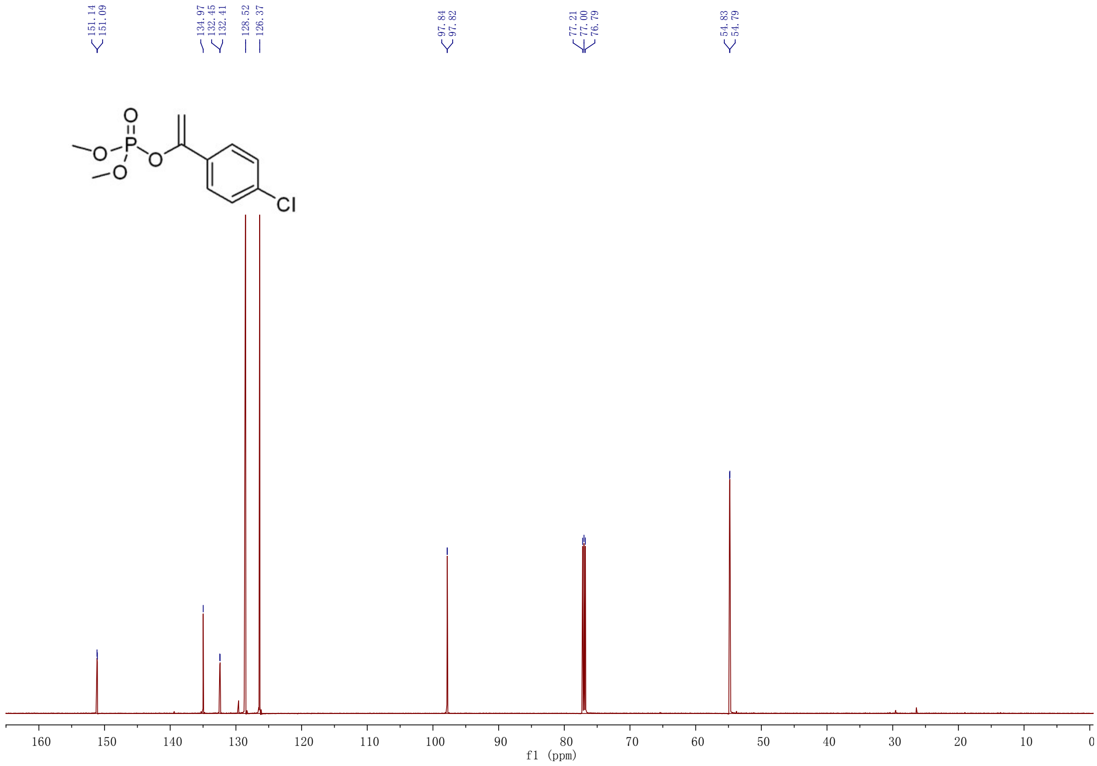

Fig.S 22 ^{13}C NMR of compound **3f**

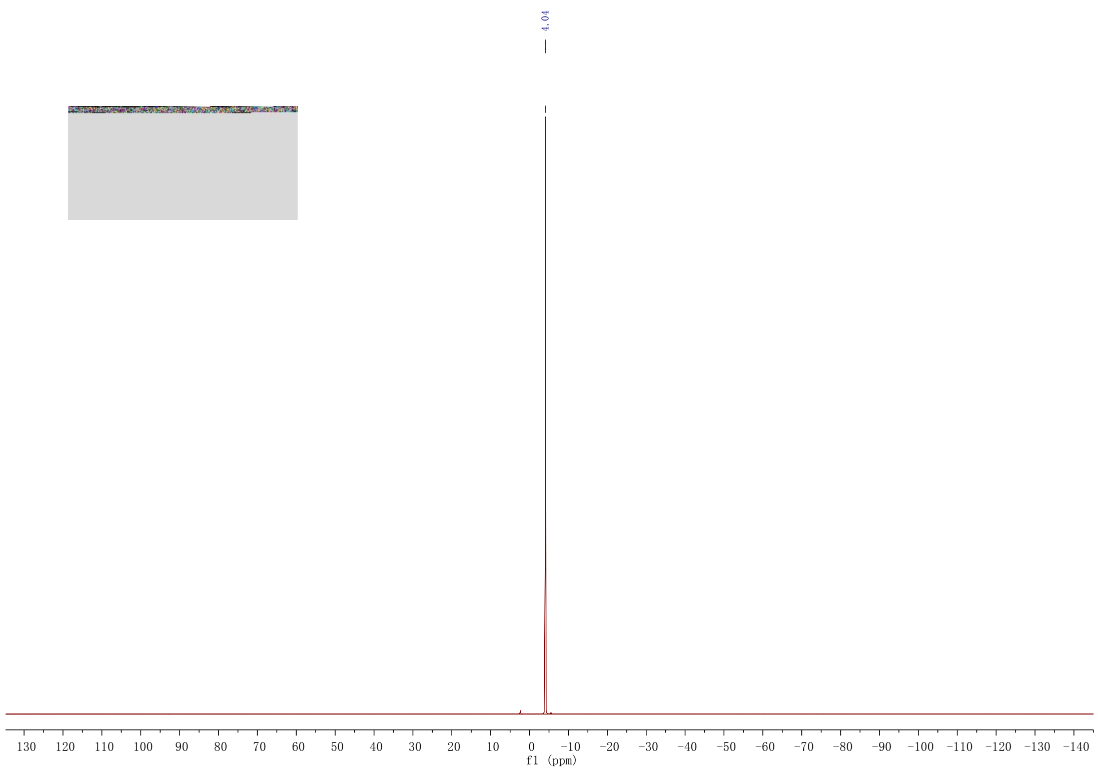

Fig.S 23 ^{31}P NMR of compound **3f**

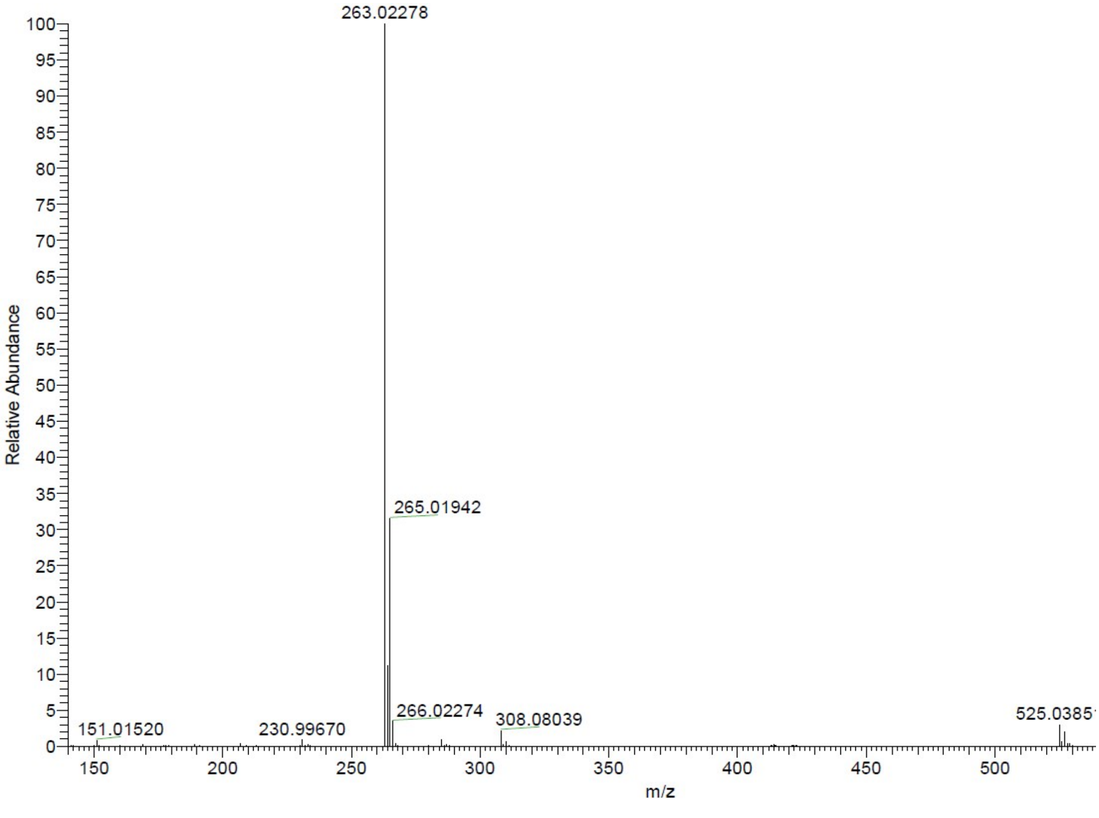

Fig.S 24 HRMS of compound **3f**

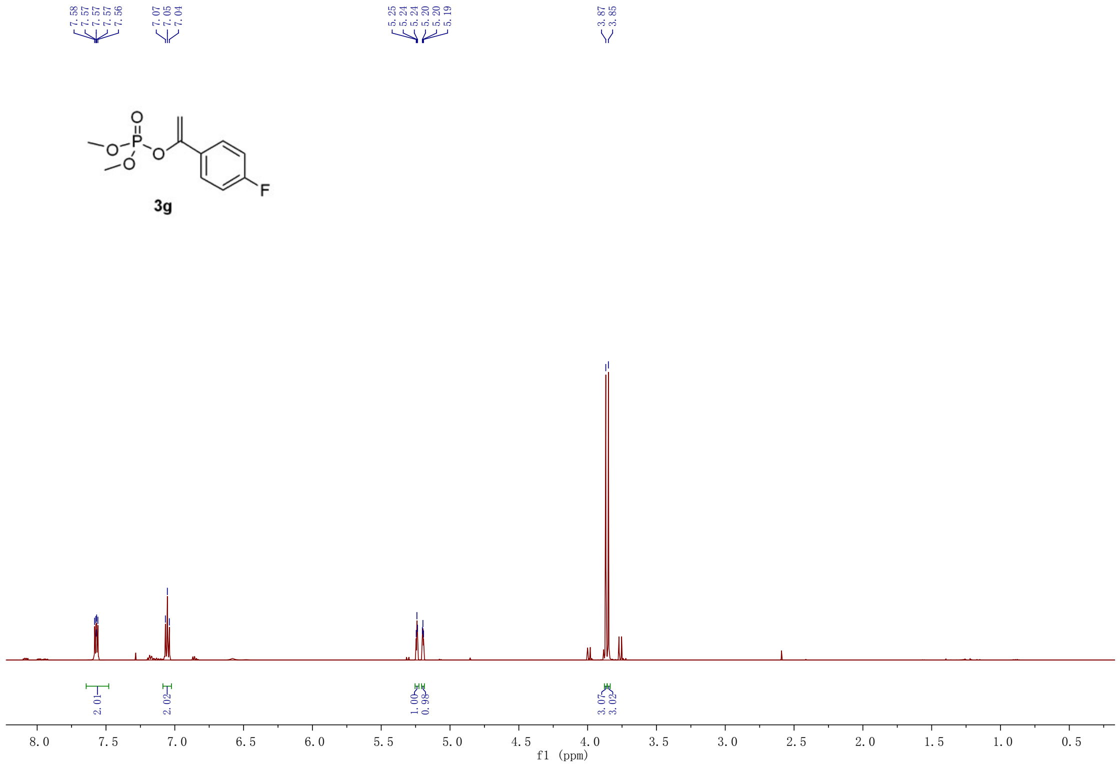

Fig.S 25 ^{1}H NMR of compound **3g**

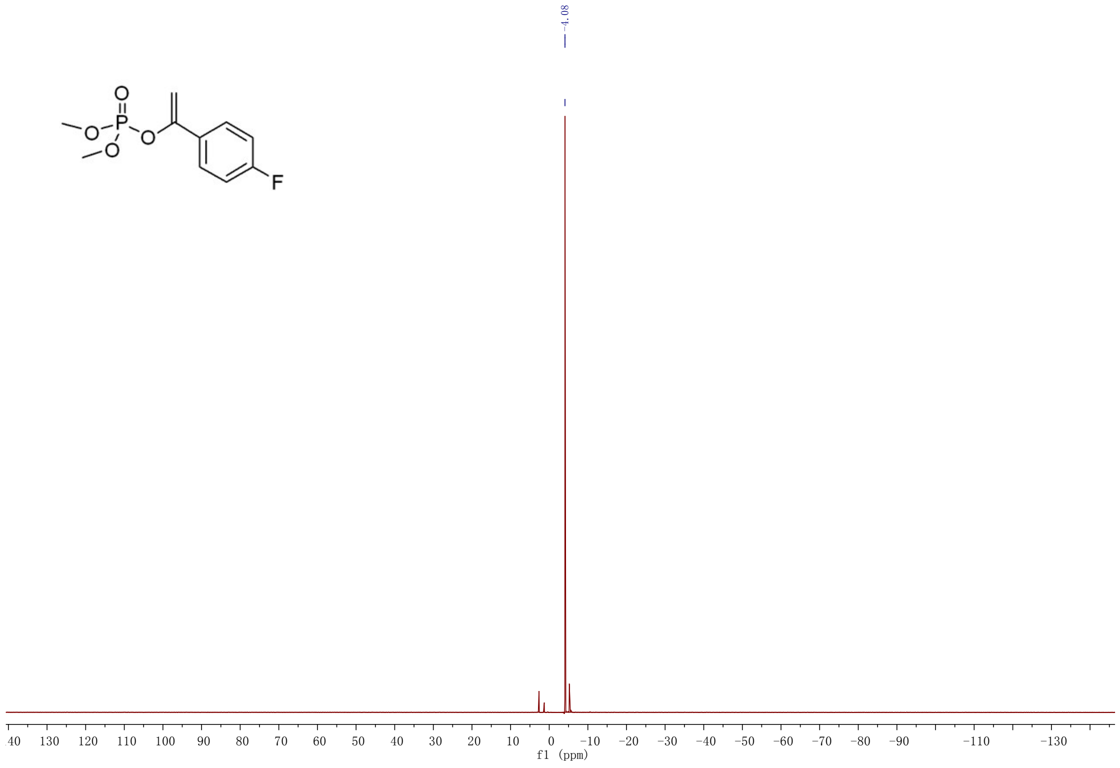

Fig.S 26 ^{13}C NMR of compound **3g**

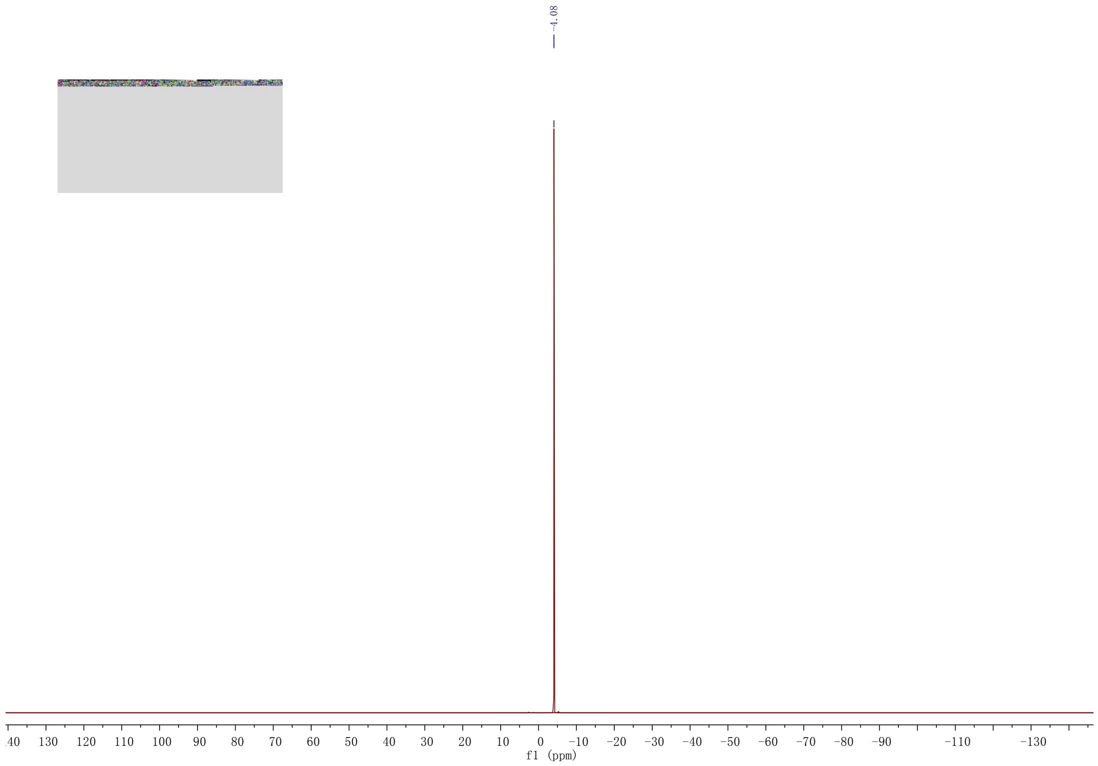

Fig.S 27 ^{31}P NMR of compound **3g**

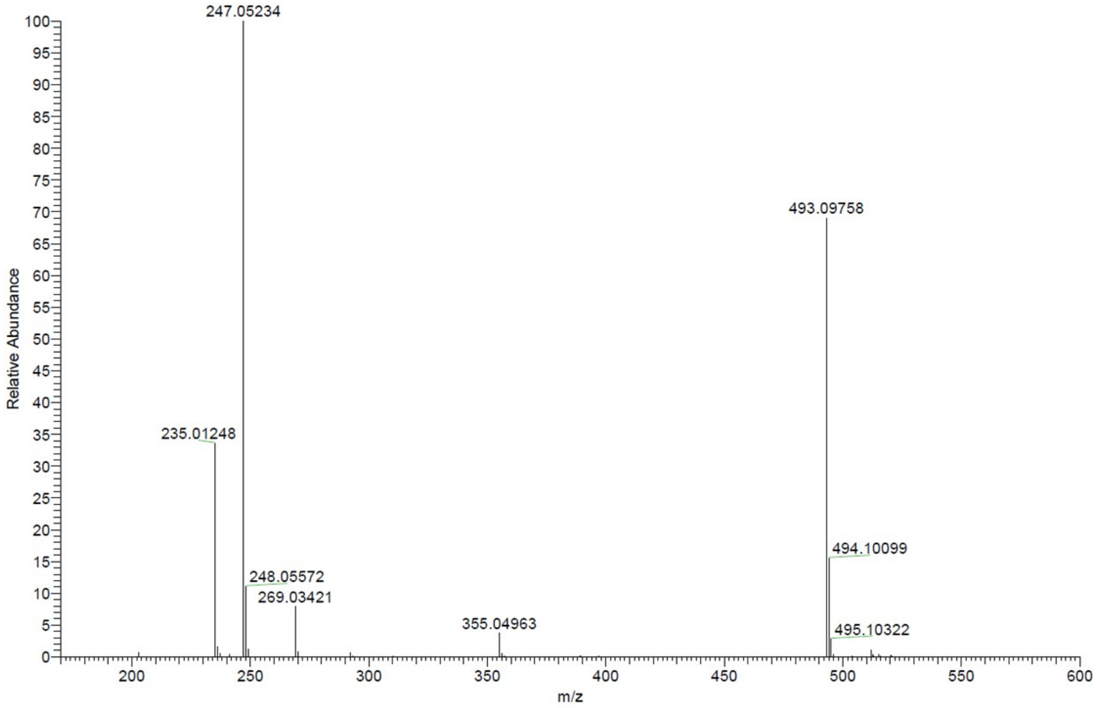

Fig.S 28 HRMS of compound **3g**

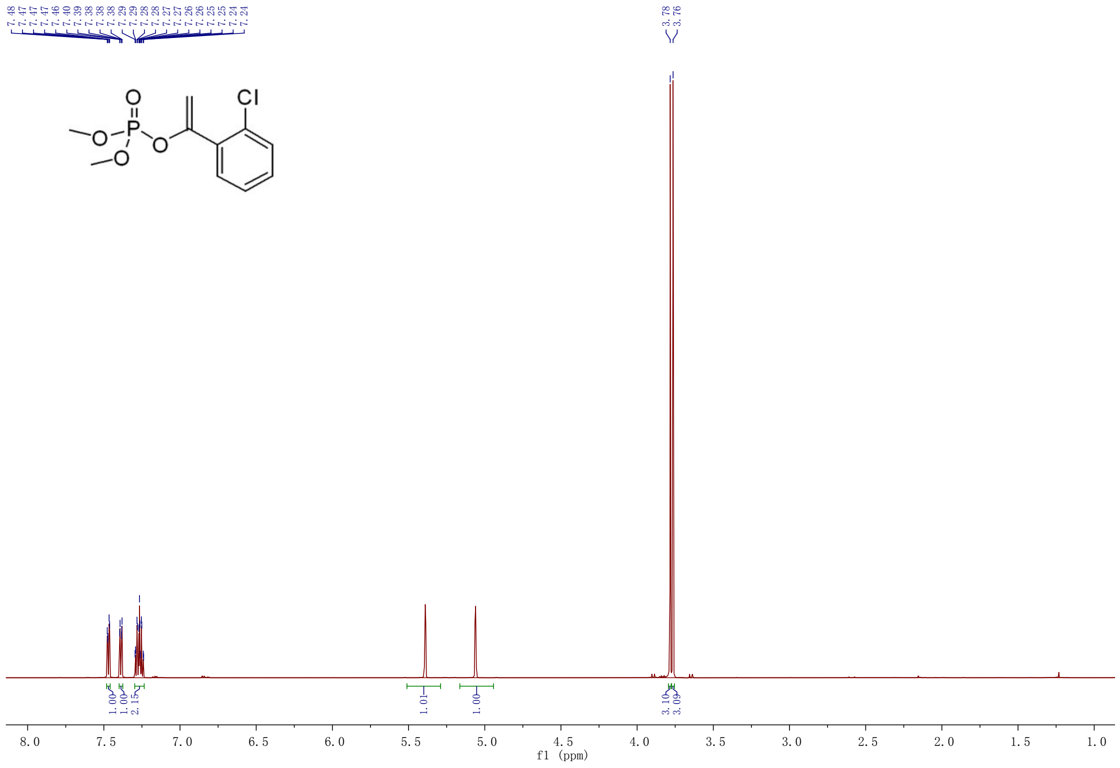

Fig.S 29 ^{1}H NMR of compound **3h**

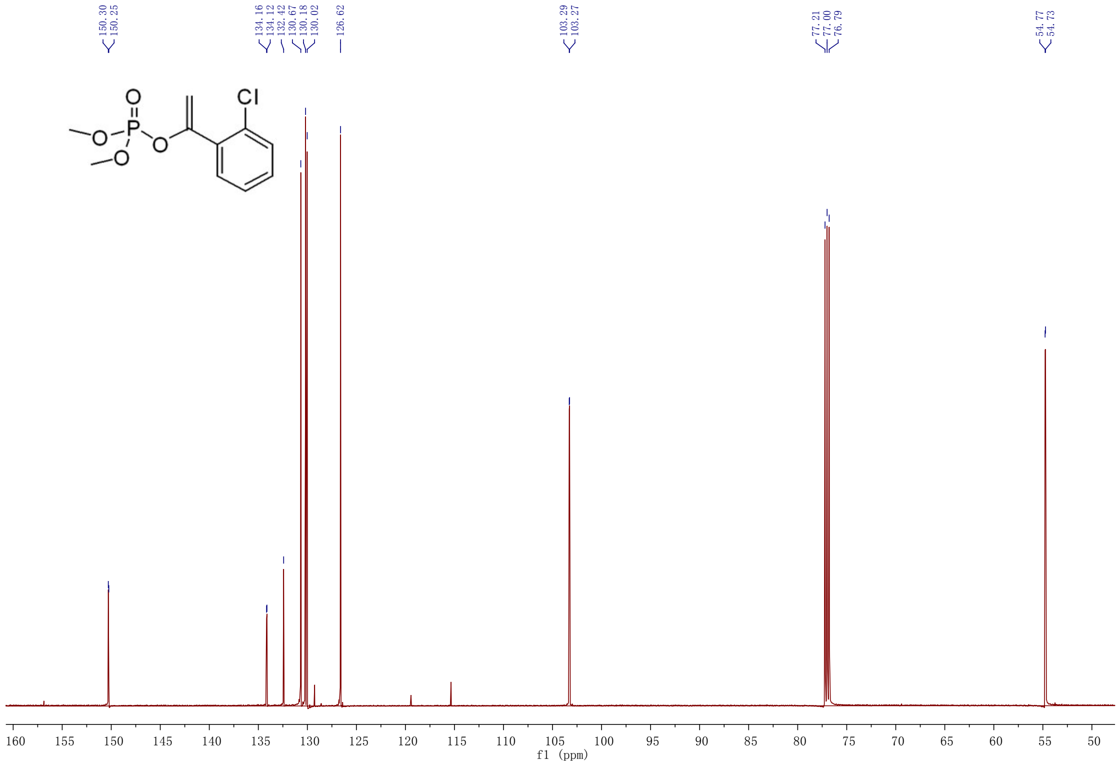

Fig.S 30 ^{13}C NMR of compound **3h**

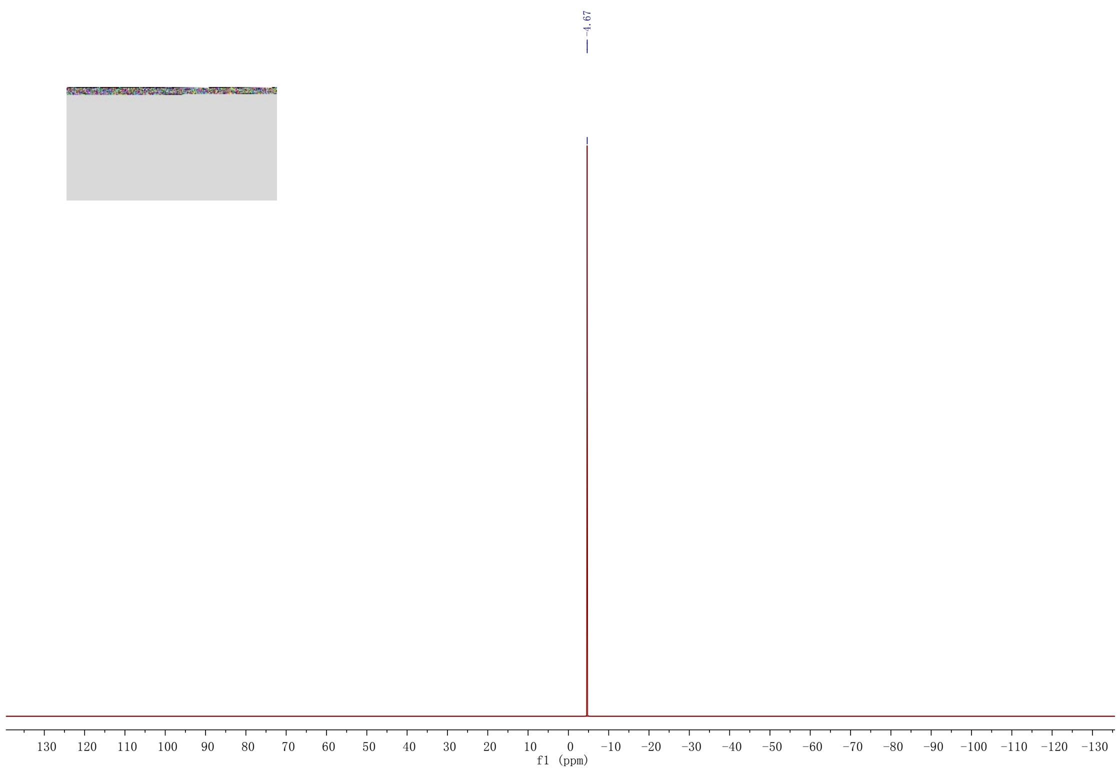

Fig.S 31 ^{31}P NMR of compound **3h**

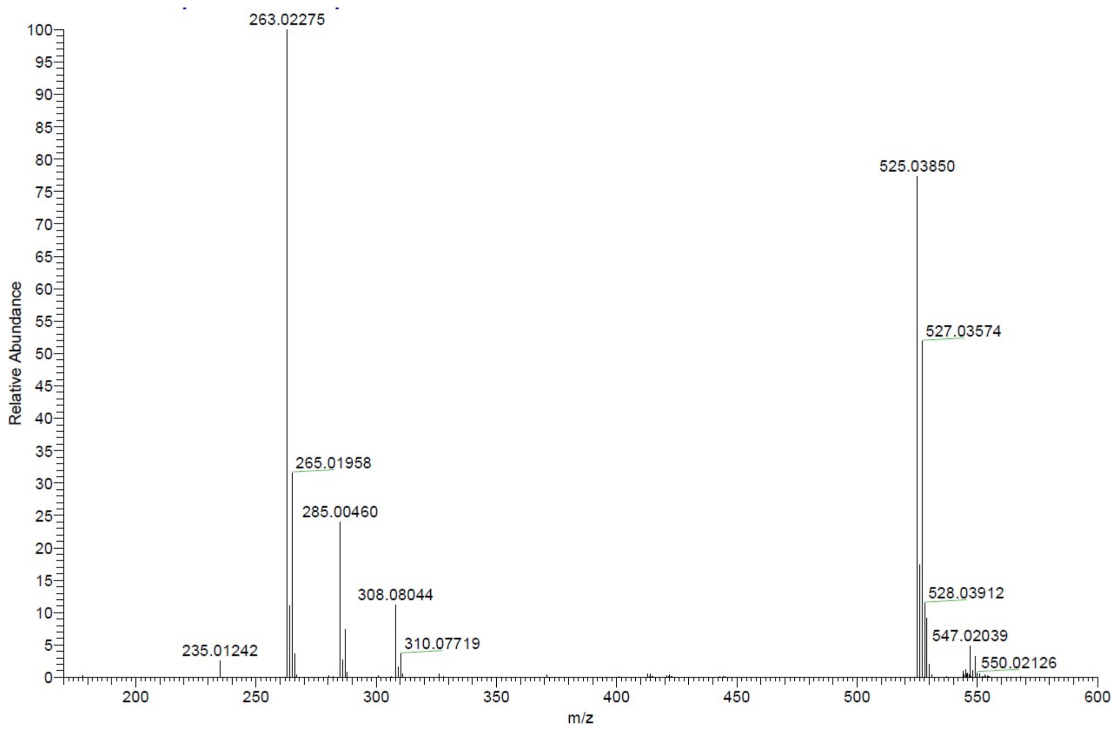

Fig.S 32 HRMS of compound **3h**

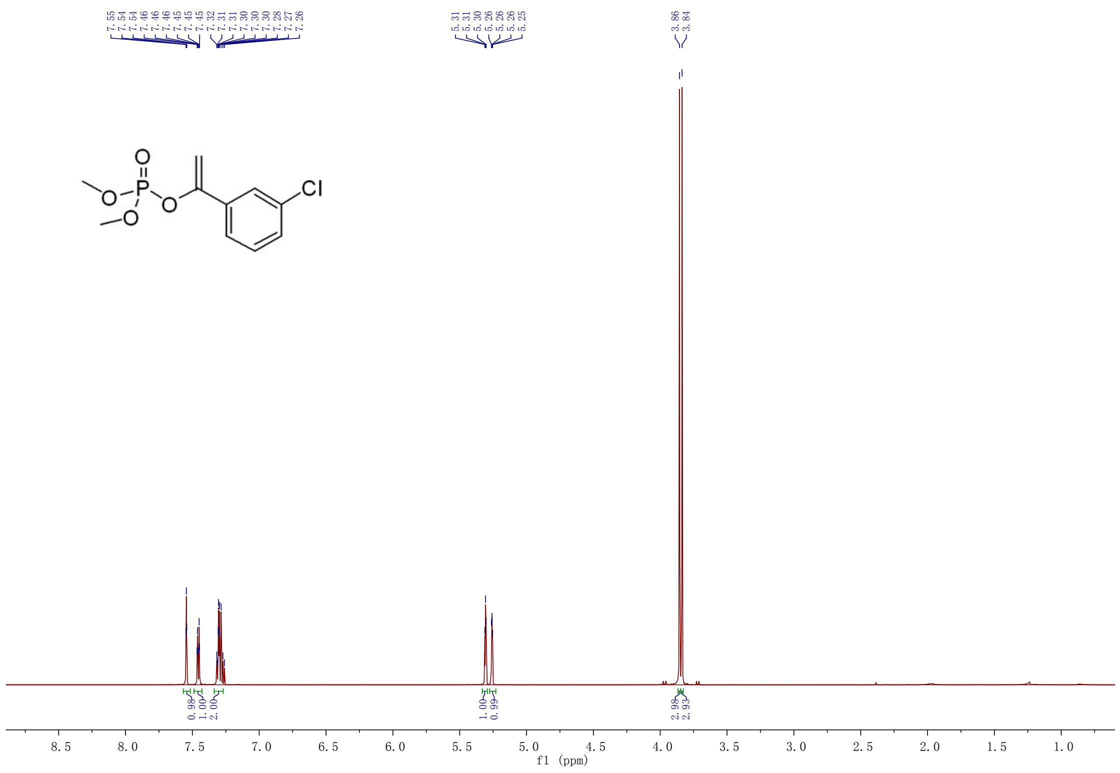

Fig.S 33 ^{1}H NMR of compound **3i**

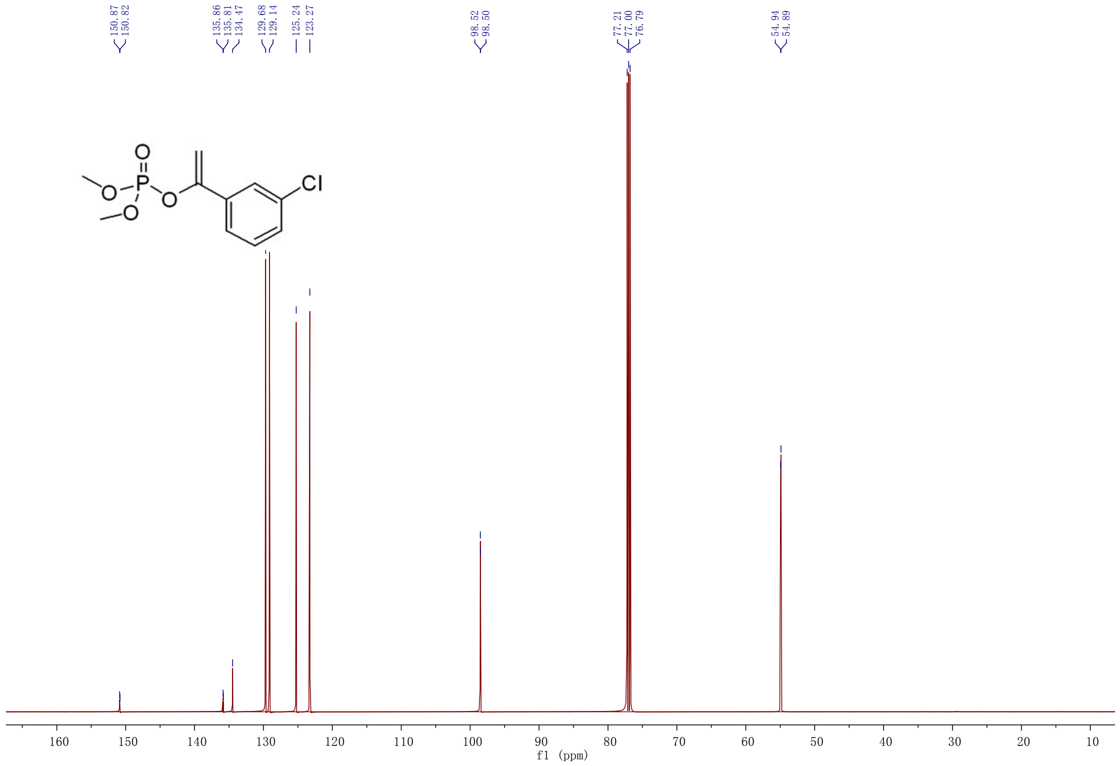

Fig.S 34 ^{13}C NMR of compound **3i**

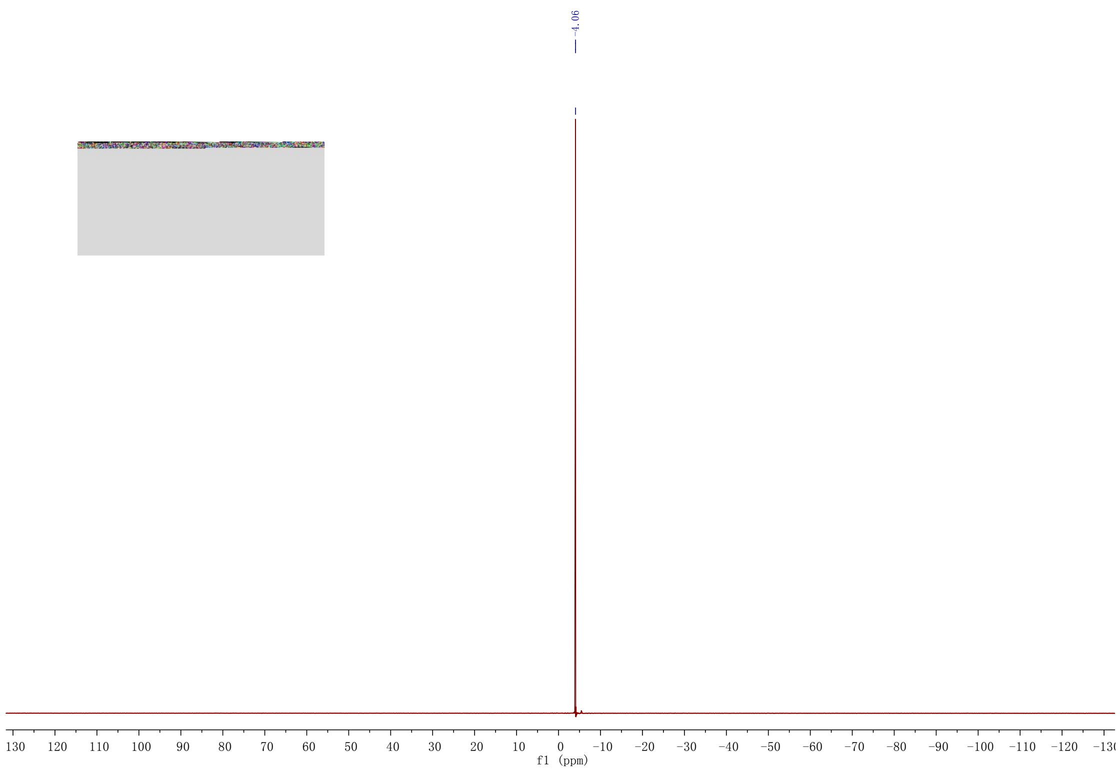

Fig.S 35 ^{31}P NMR of compound **3i**

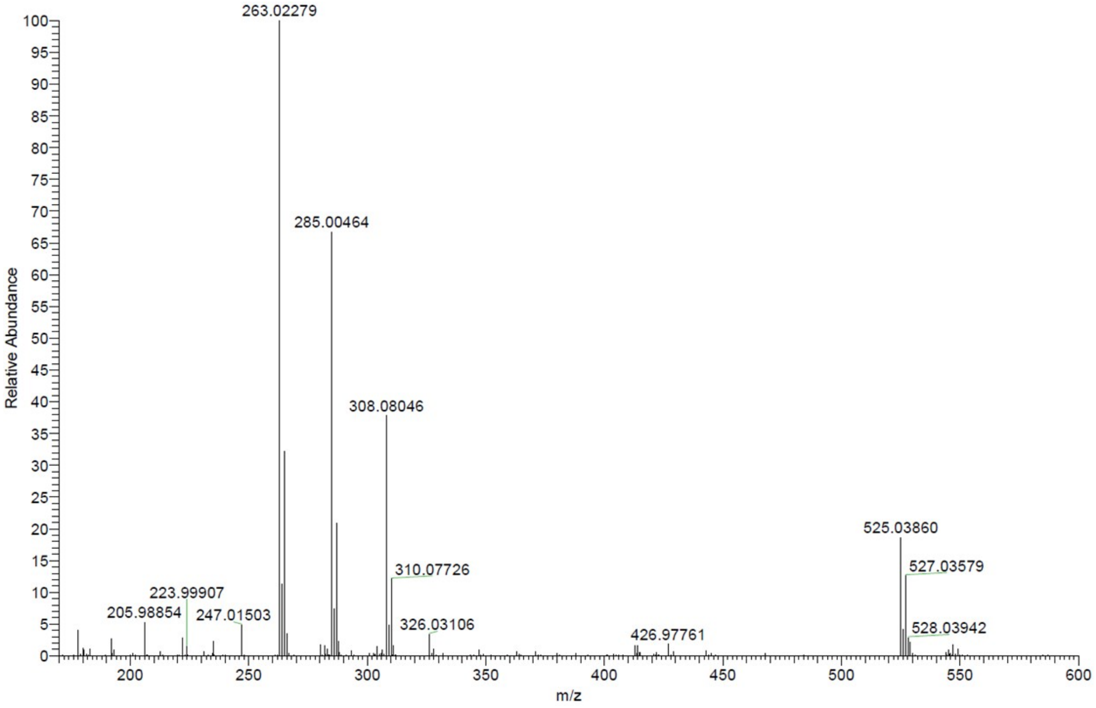

Fig.S 36 HRMS of compound **3i**

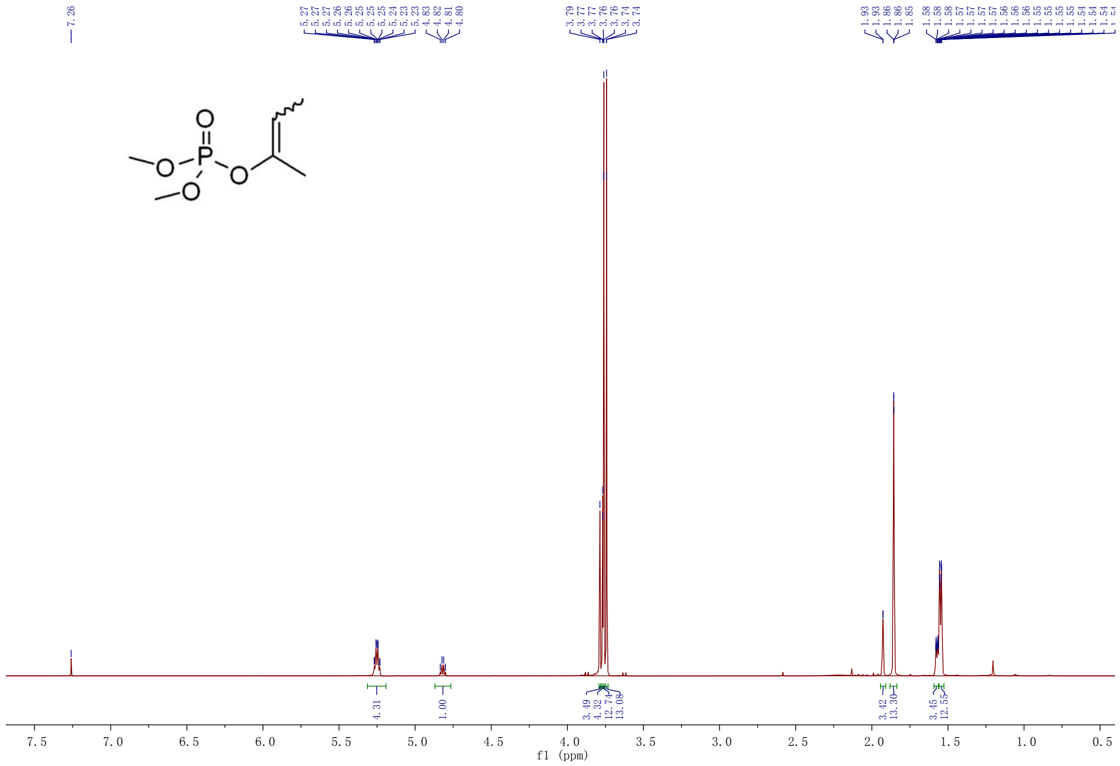

Fig.S 37 ^{1}H NMR of compound **3j**

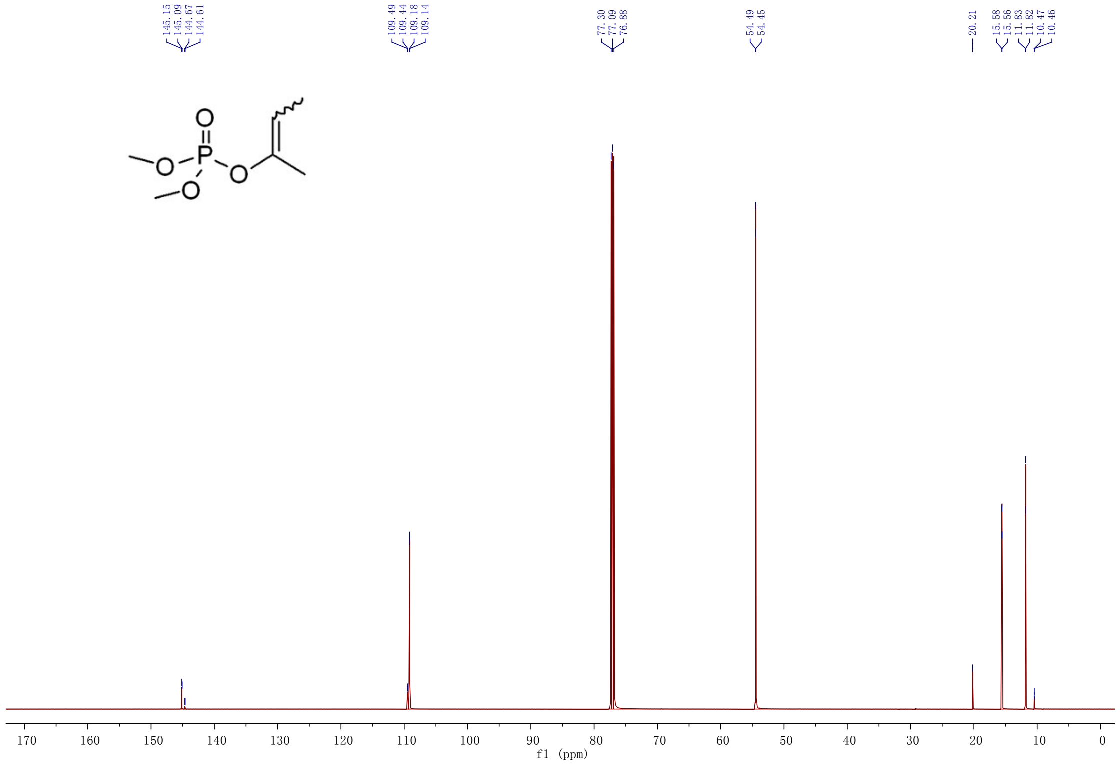

Fig.S 38 ^{13}C NMR of compound **3j**

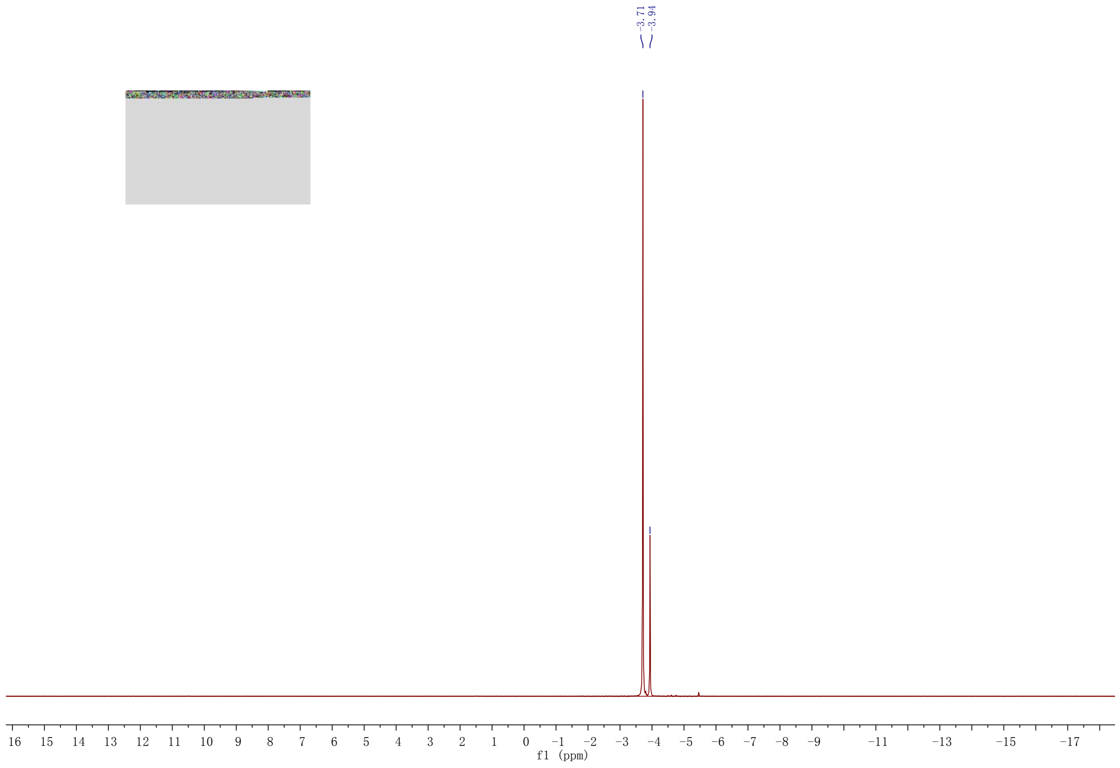

Fig.S 39 ^{31}P NMR of compound **3j**

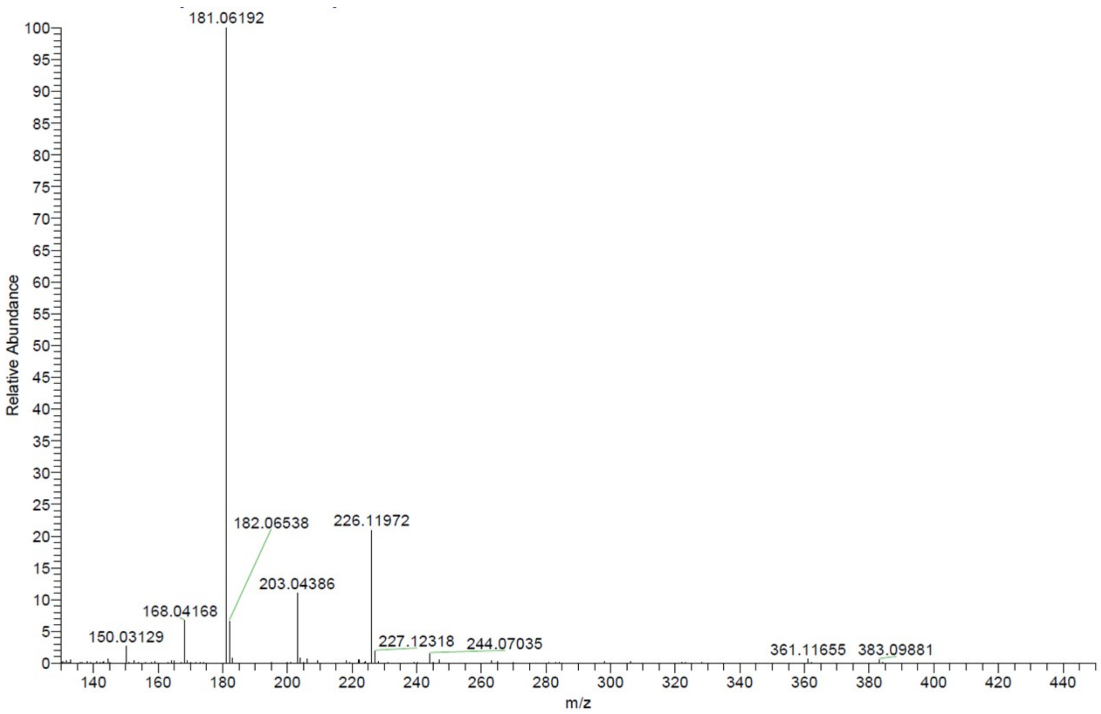

Fig.S 40 HRMS of compound **3j**

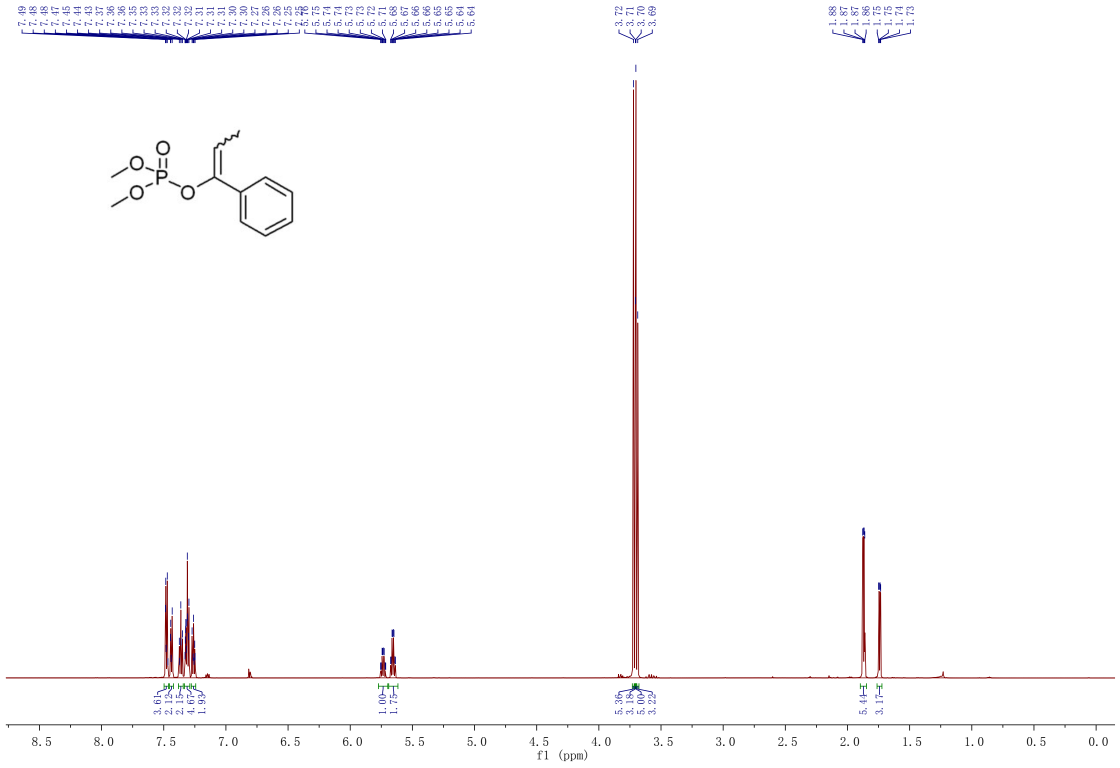

Fig.S 41 ^{1}H NMR of compound **3k**

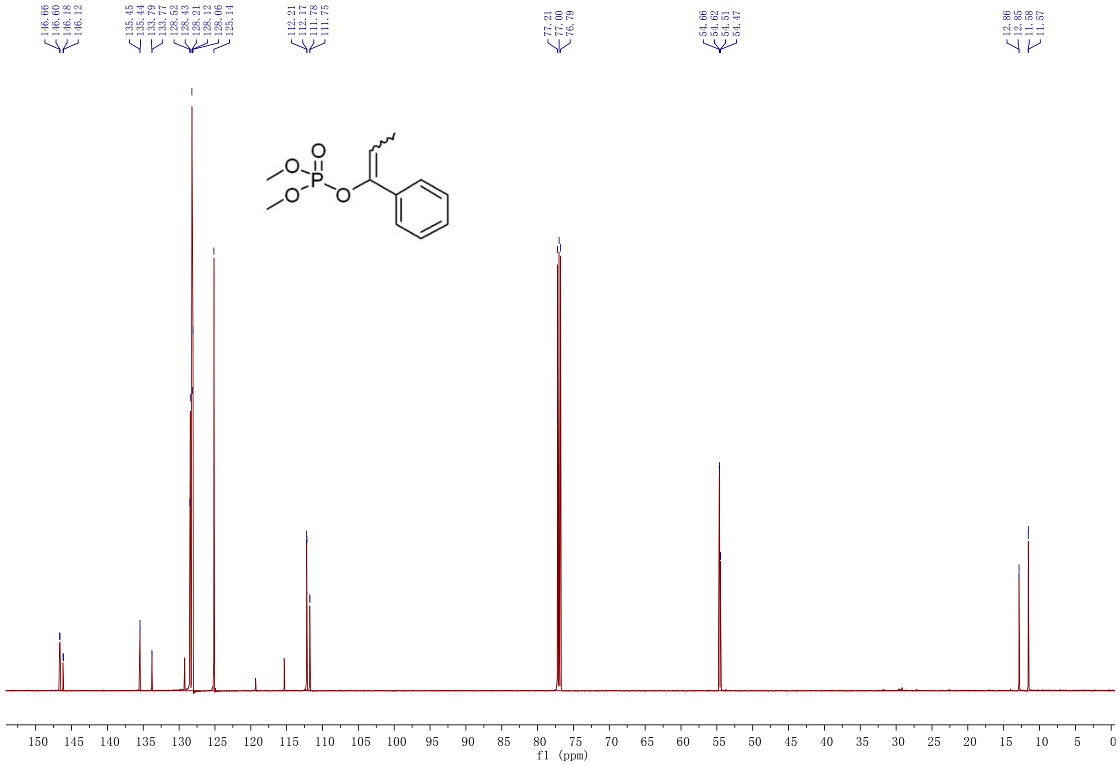

Fig.S 42 ^{13}C NMR of compound **3k**

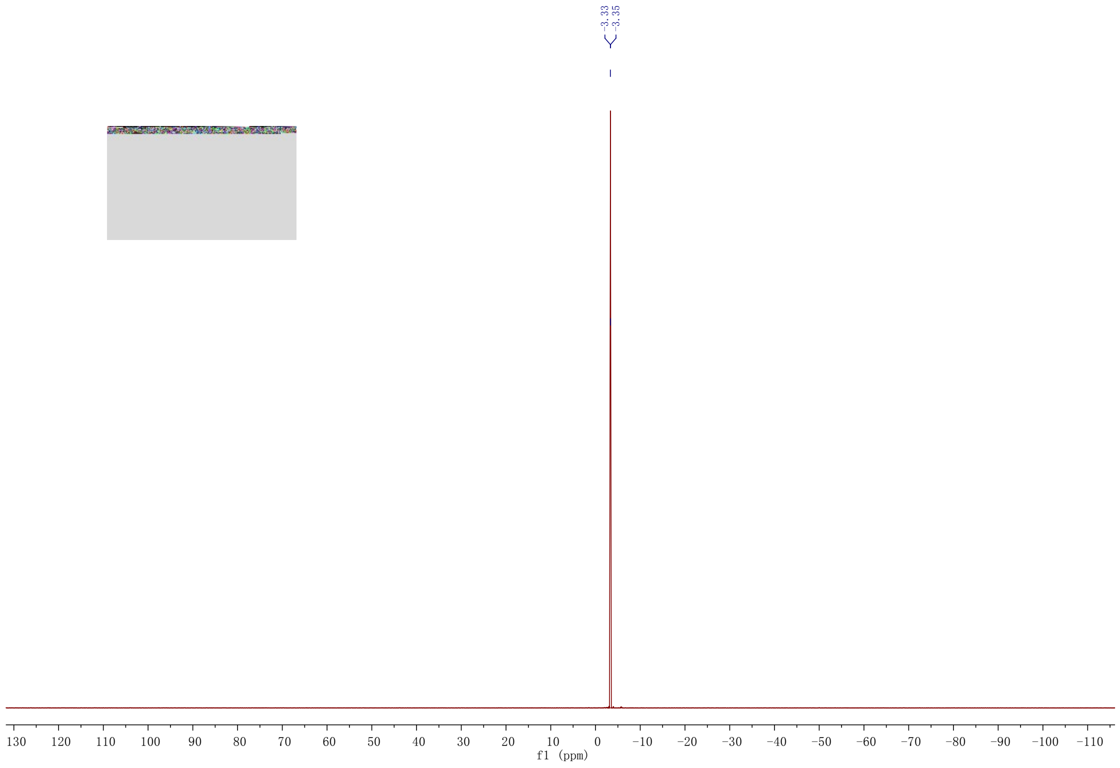

Fig.S 43 ^{31}P NMR of compound **3k**

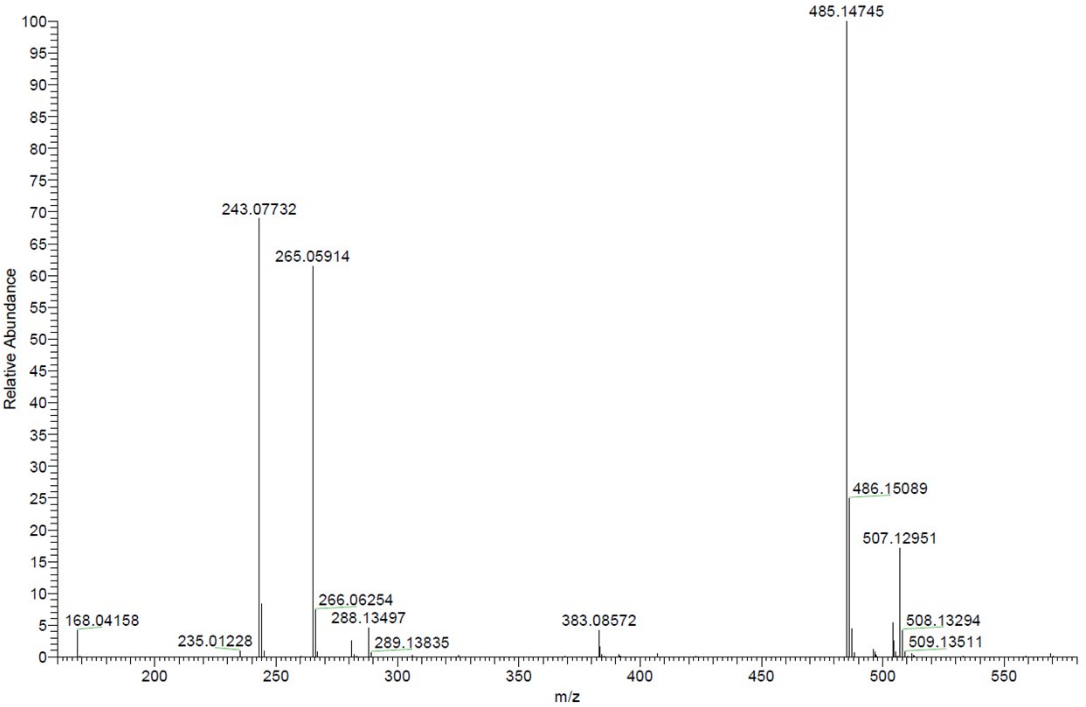

Fig.S 44 HRMS of compound **3k**

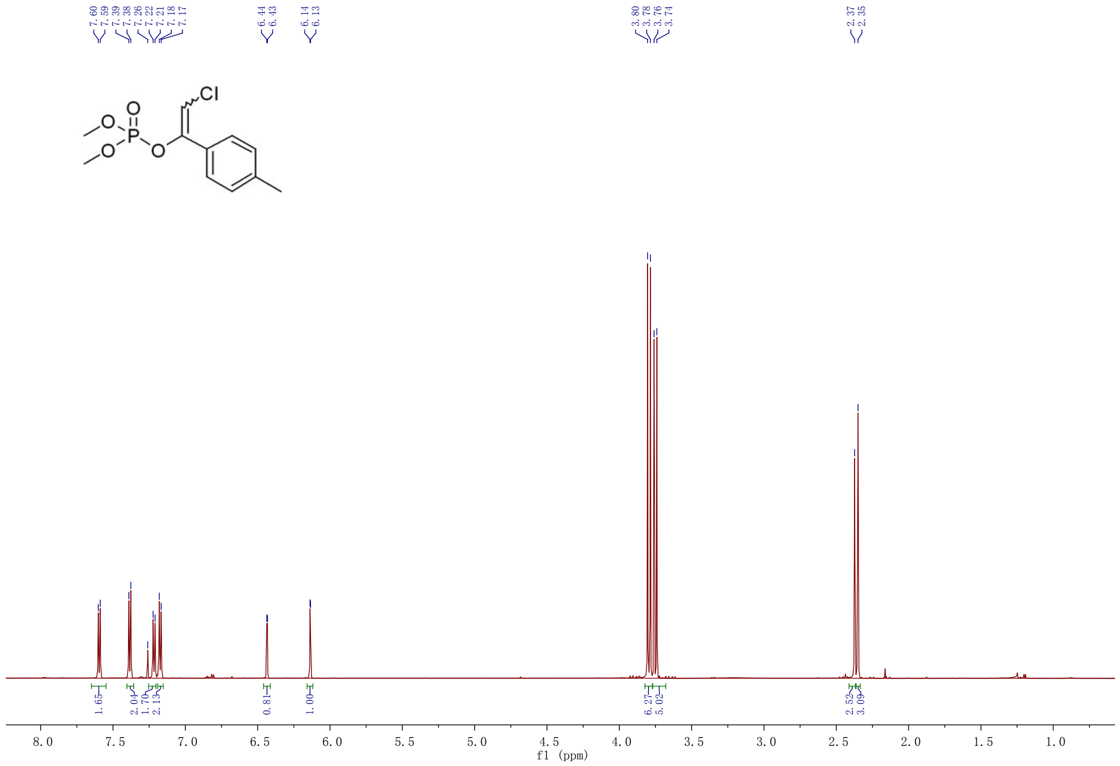

Fig.S 45 ^{1}H NMR of compound **3l**

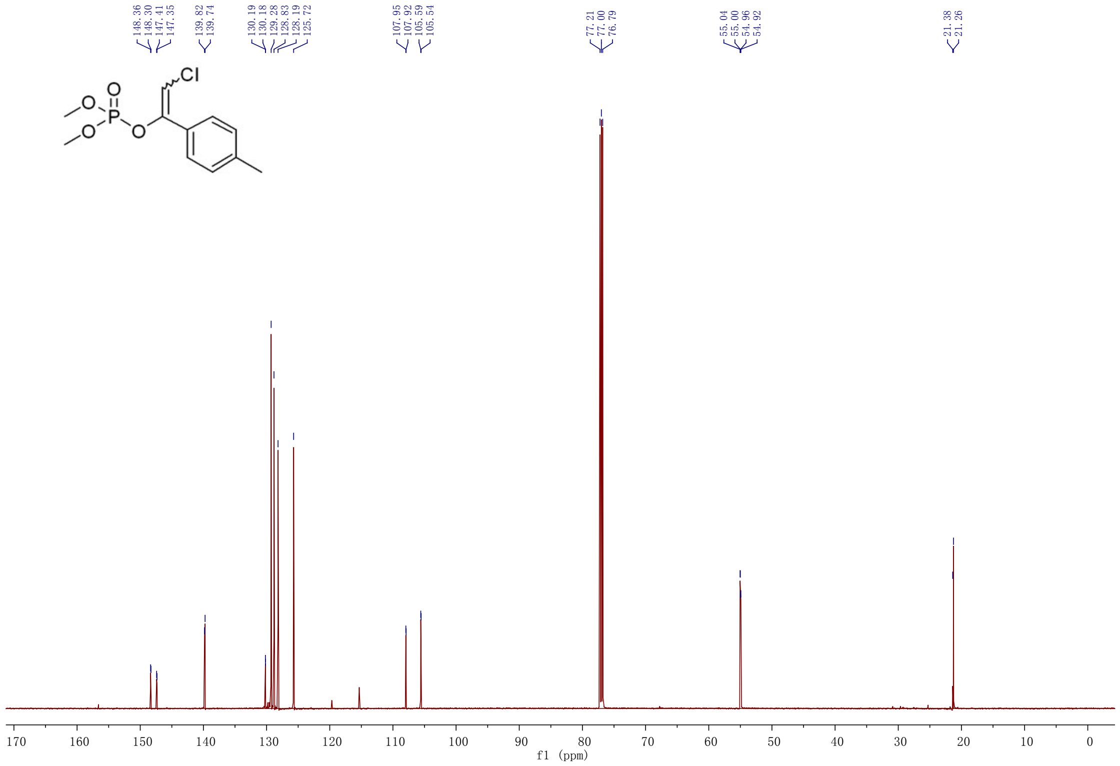

Fig.S 46 ^{13}C NMR of compound **3l**

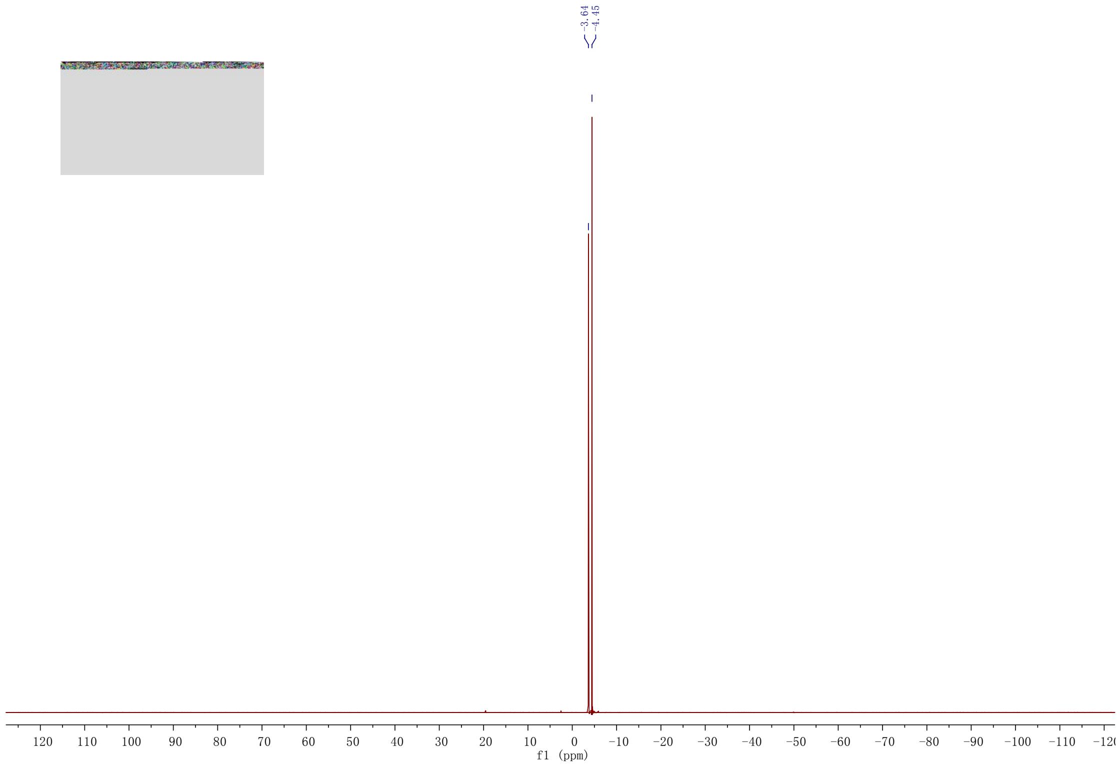

Fig.S 47 ^{31}P NMR of compound **3l**

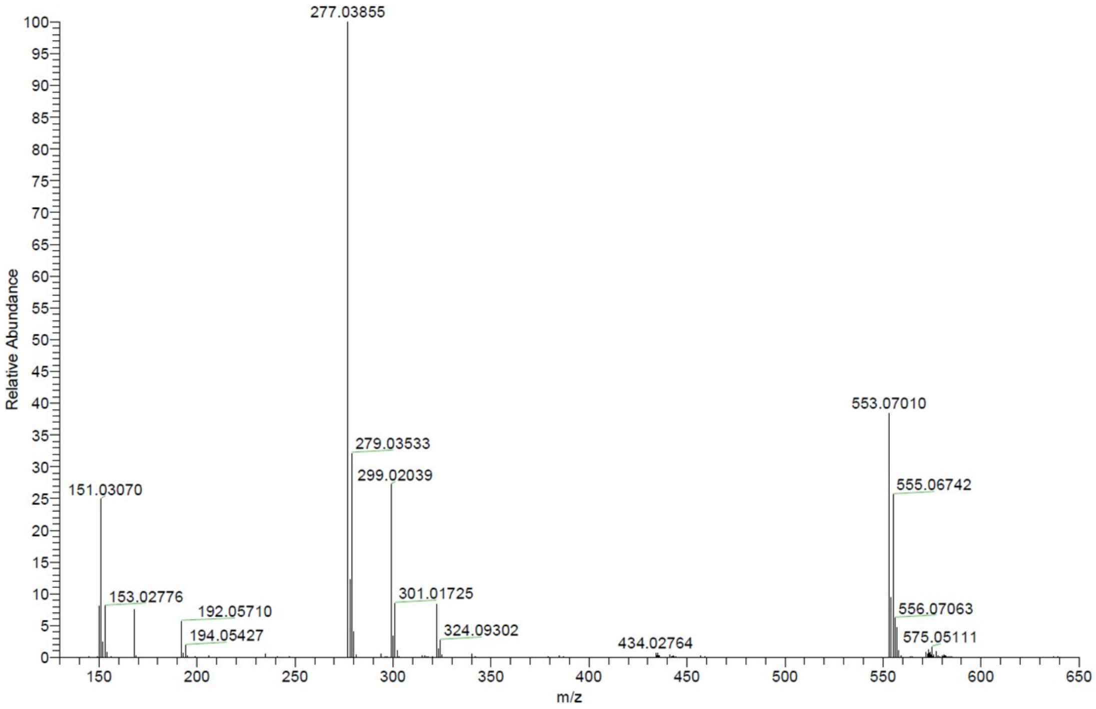

Fig.S 48 HRMS of compound **3l**

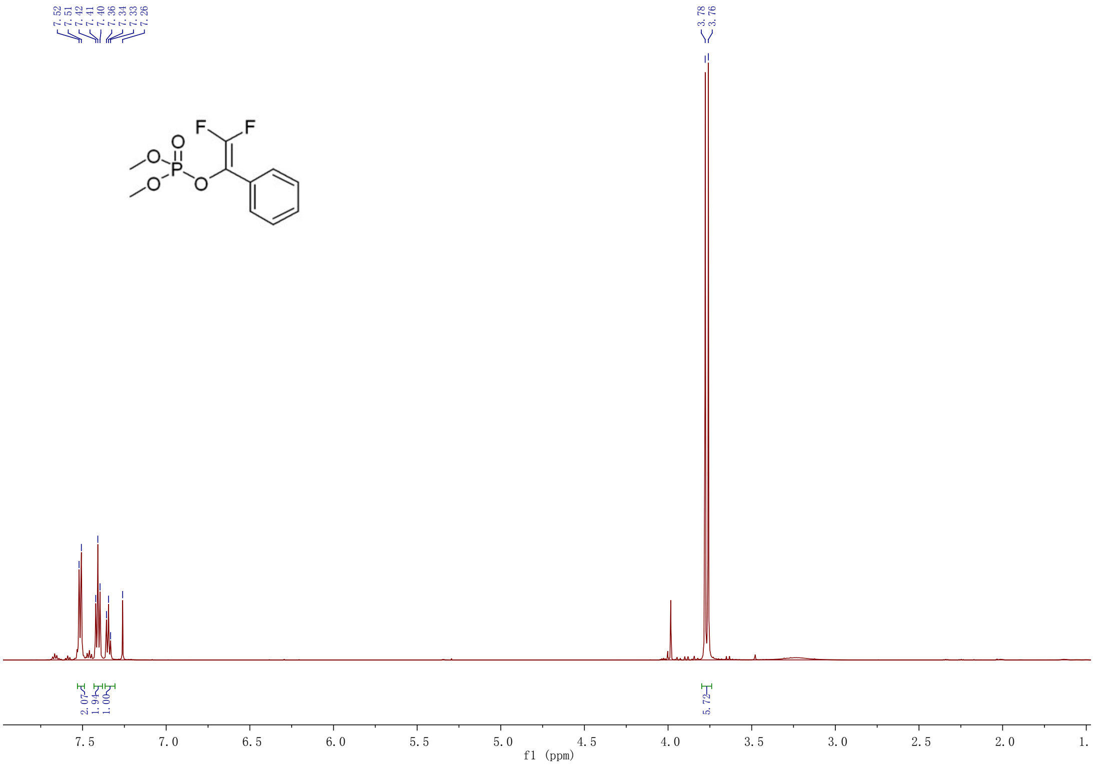

Fig.S 49 ^{1}H NMR of compound **3m**

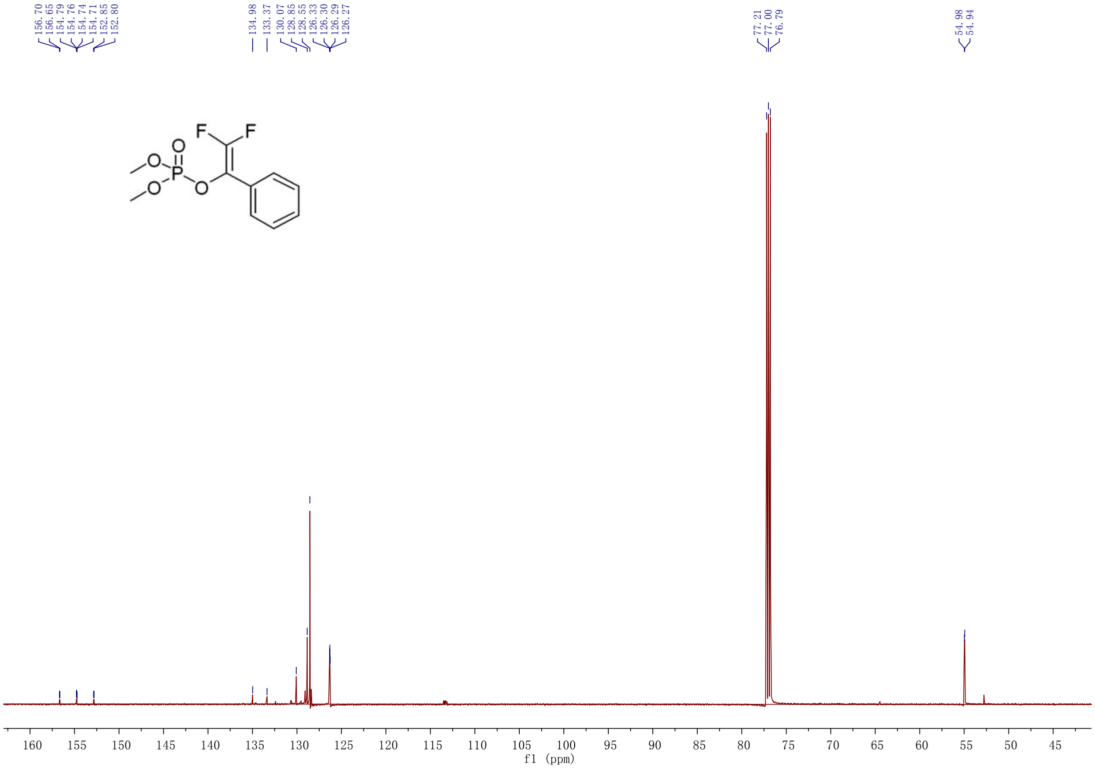

Fig.S 50 ^{13}C NMR of compound **3m**

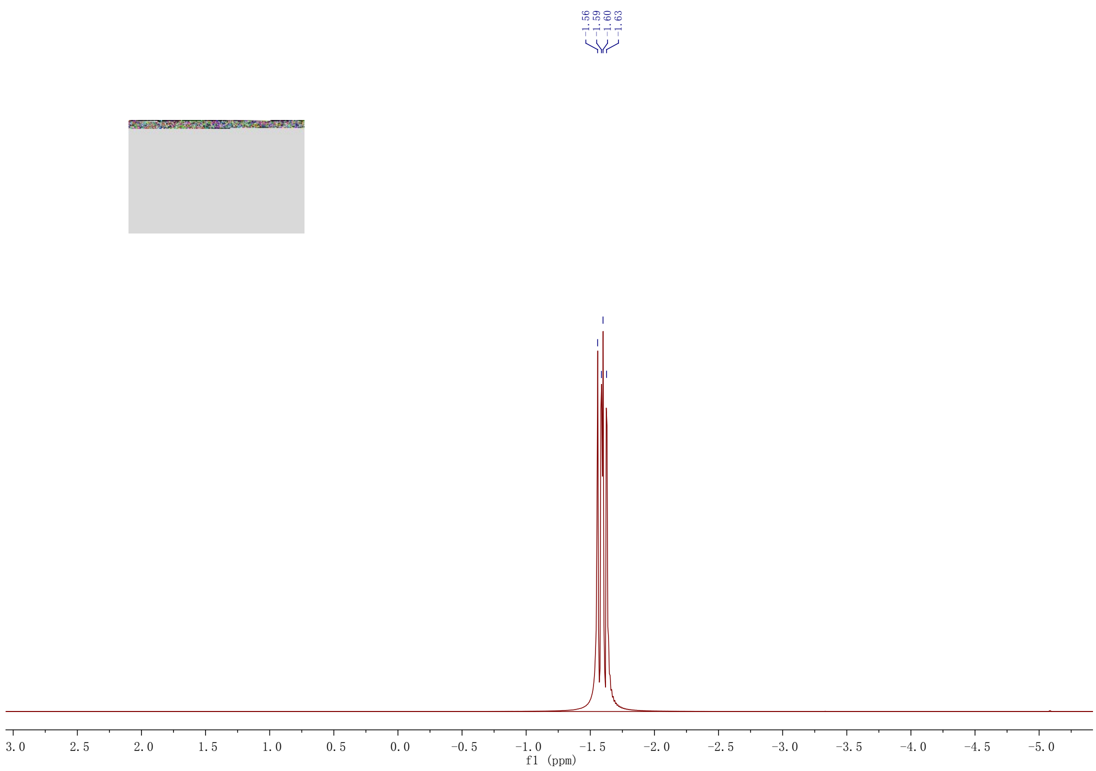

Fig.S 51 ^{31}P NMR of compound **3m**

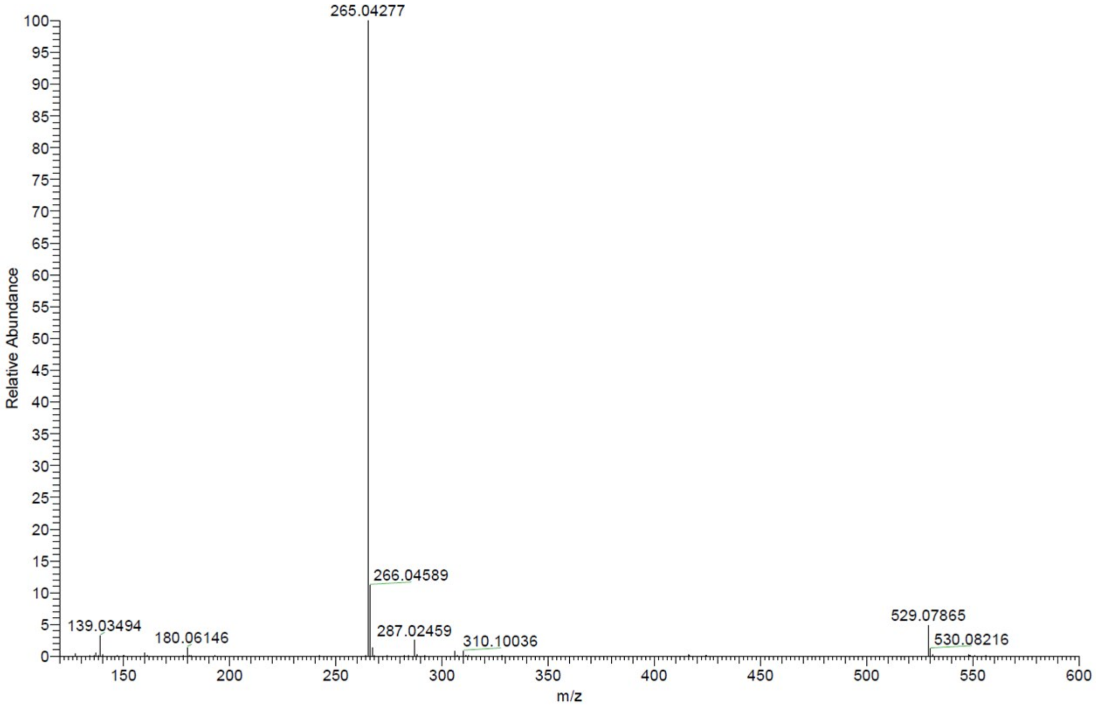

Fig.S 52 HRMS of compound **3m**

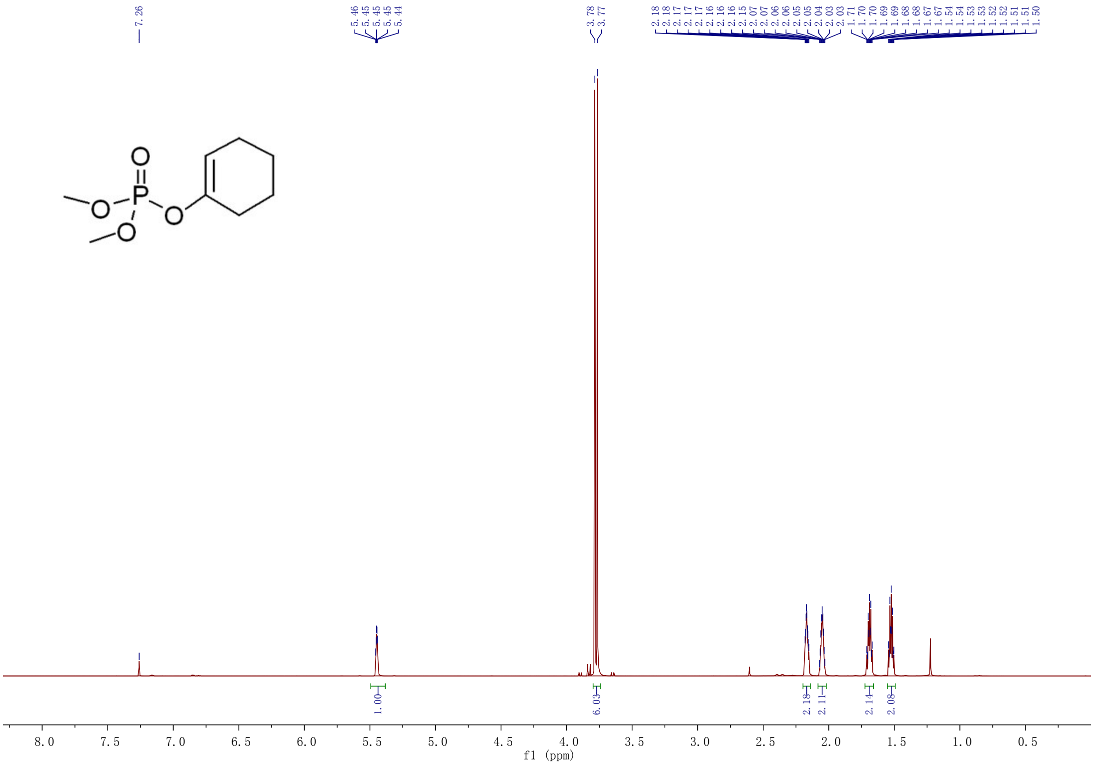

Fig.S 53 ^{1}H NMR of compound **3n**

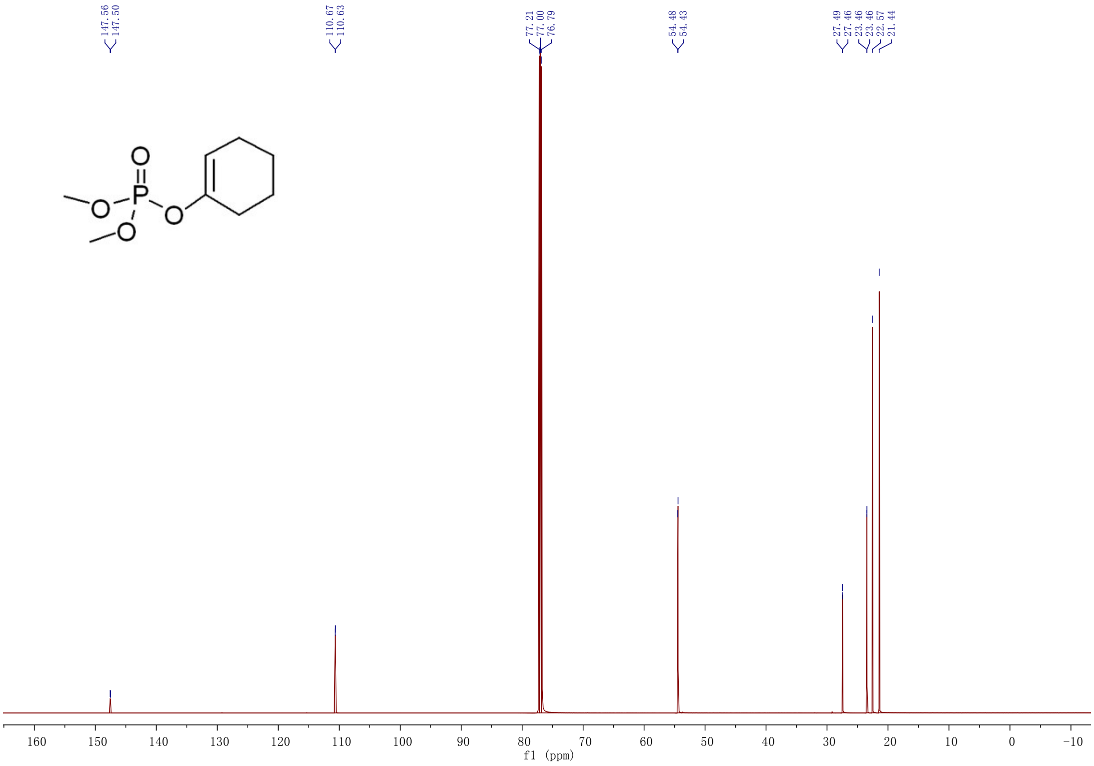

Fig.S 54 ^{13}C NMR of compound **3n**

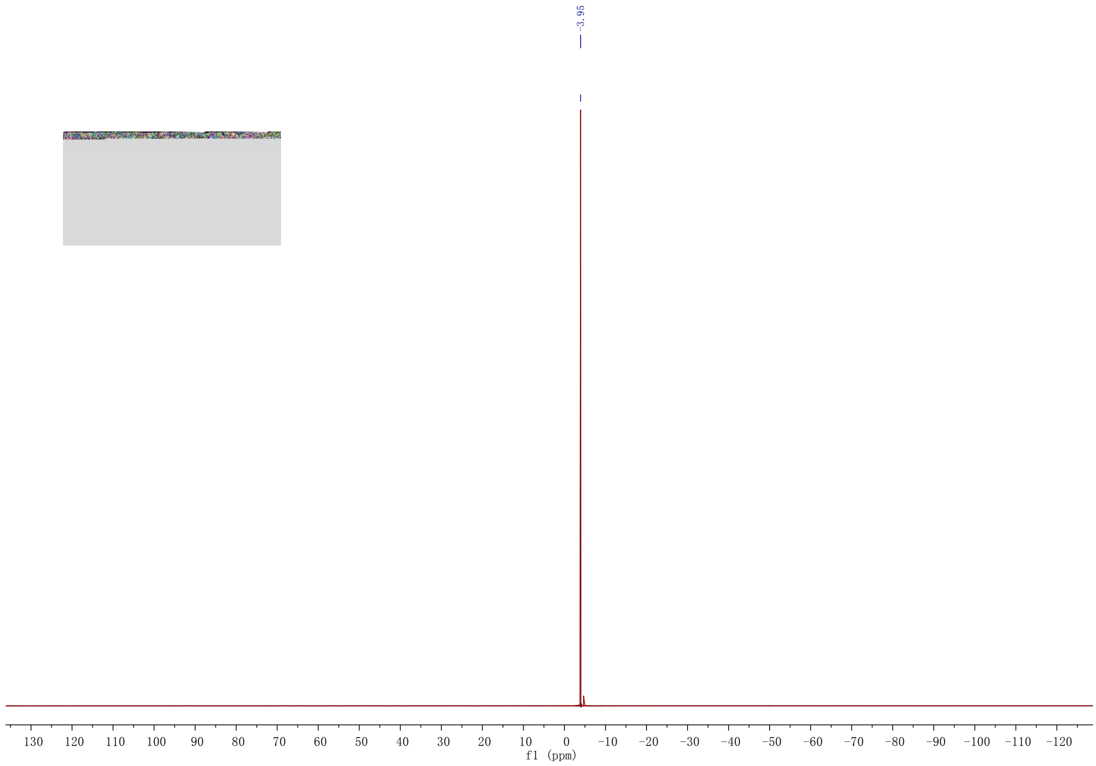

Fig.S 55 ^{31}P NMR of compound **3n**

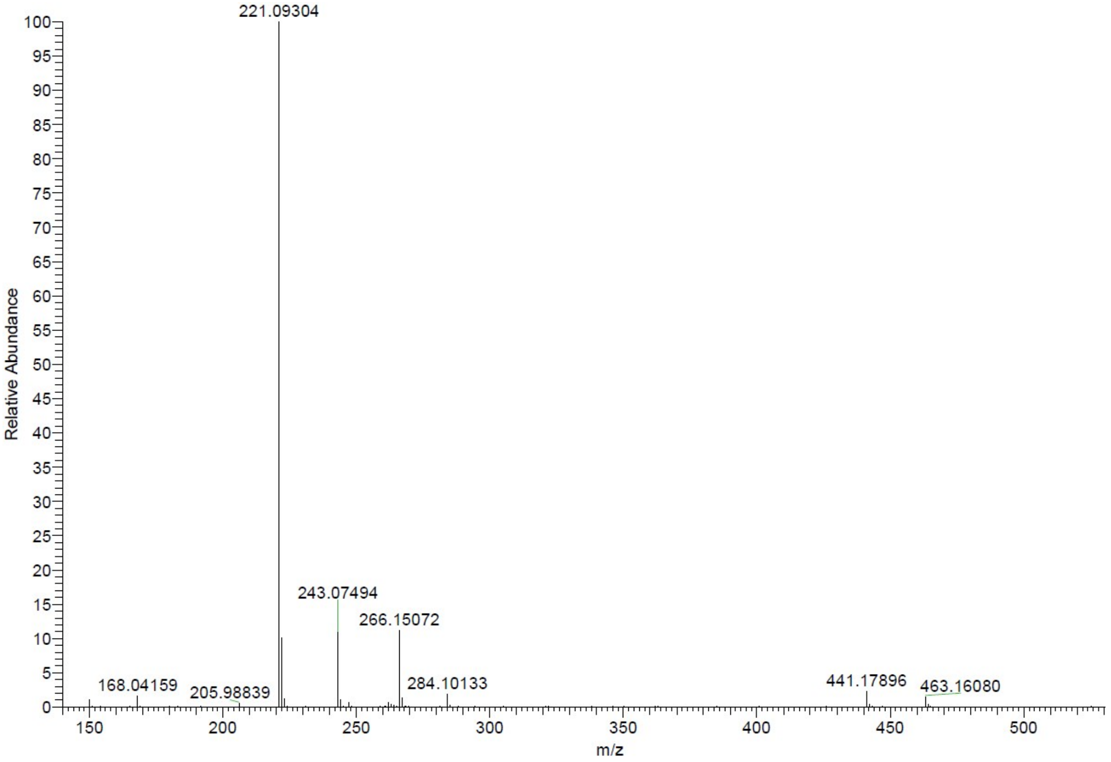

Fig.S 56 HRMS of compound **3n**

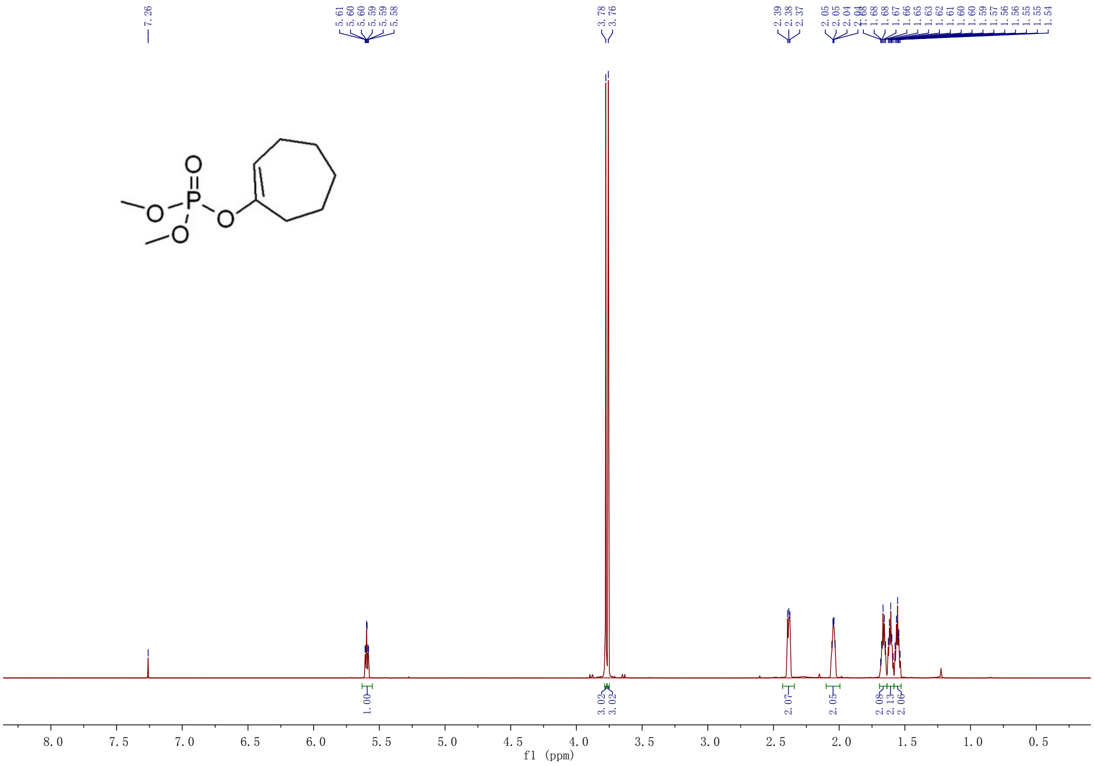

Fig.S 57 ^{1}H NMR of compound **3o**

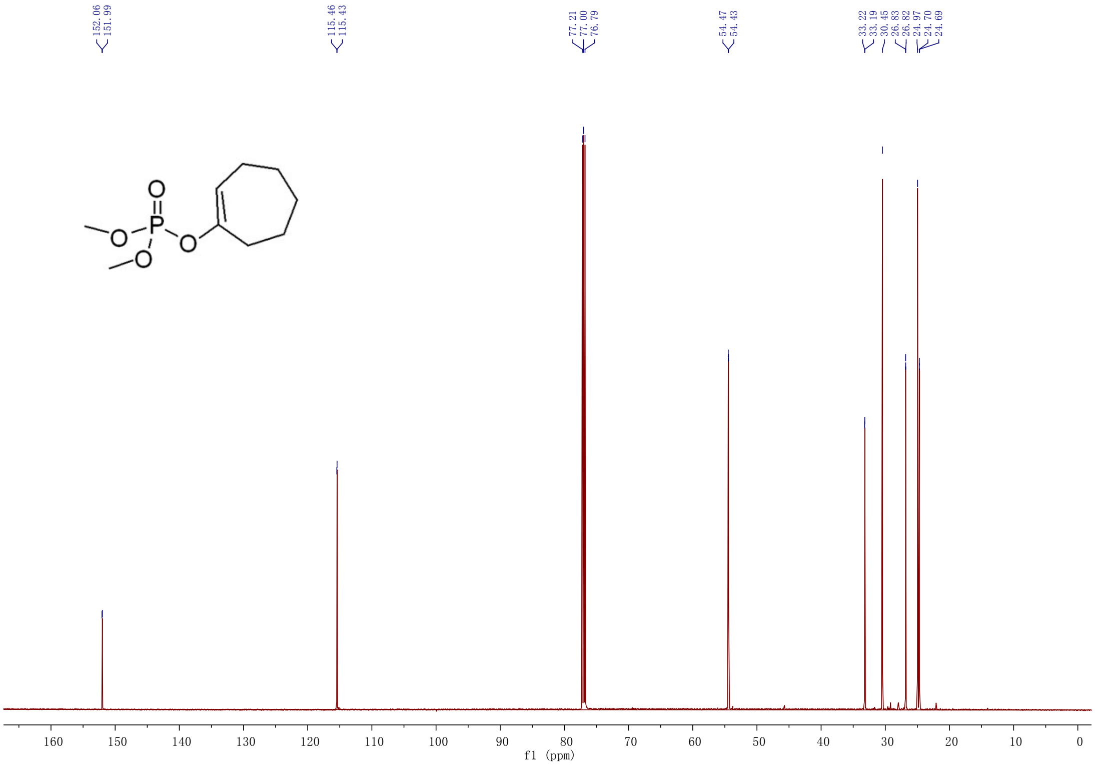

Fig.S 58 ^{13}C NMR of compound **3o**

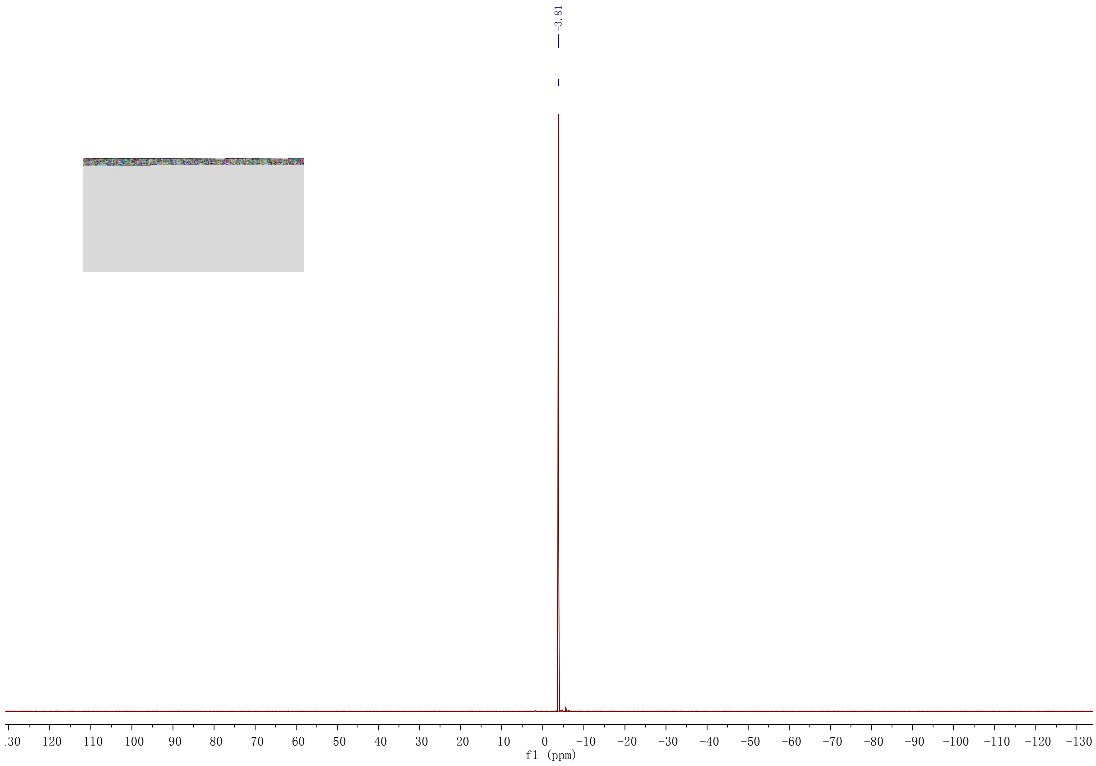

Fig.S 59 ^{31}P NMR of compound **3o**

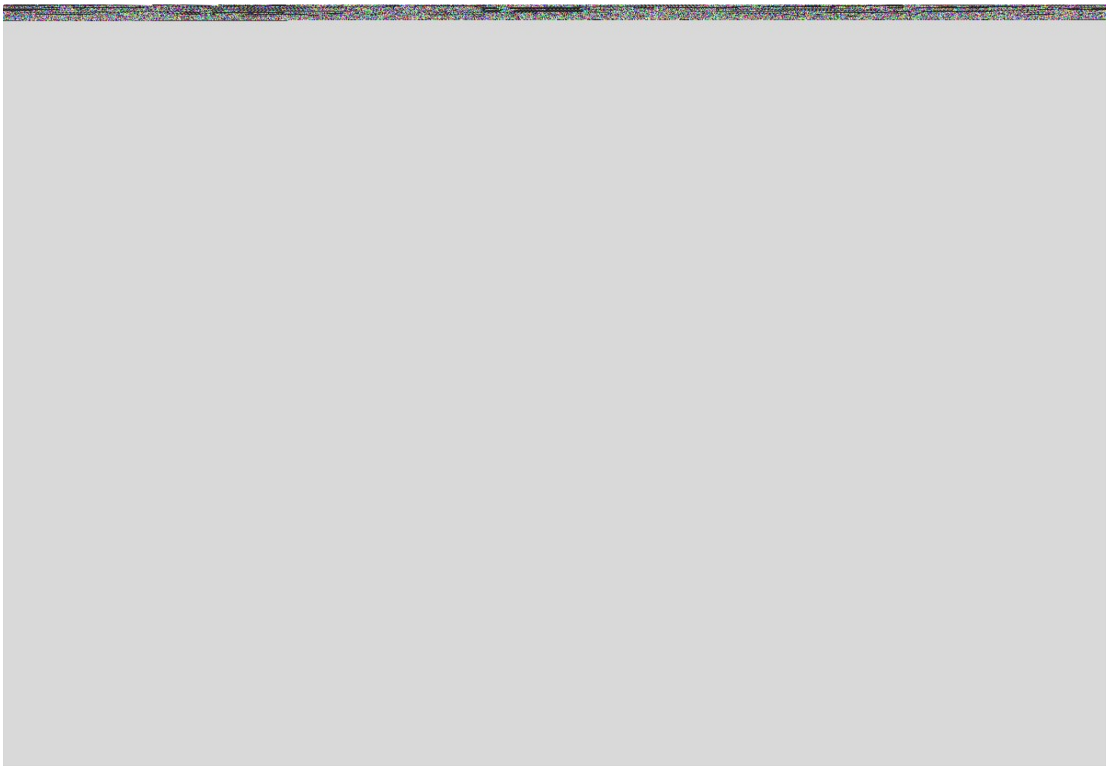

Fig.S 60 HRMS of compound **3o**

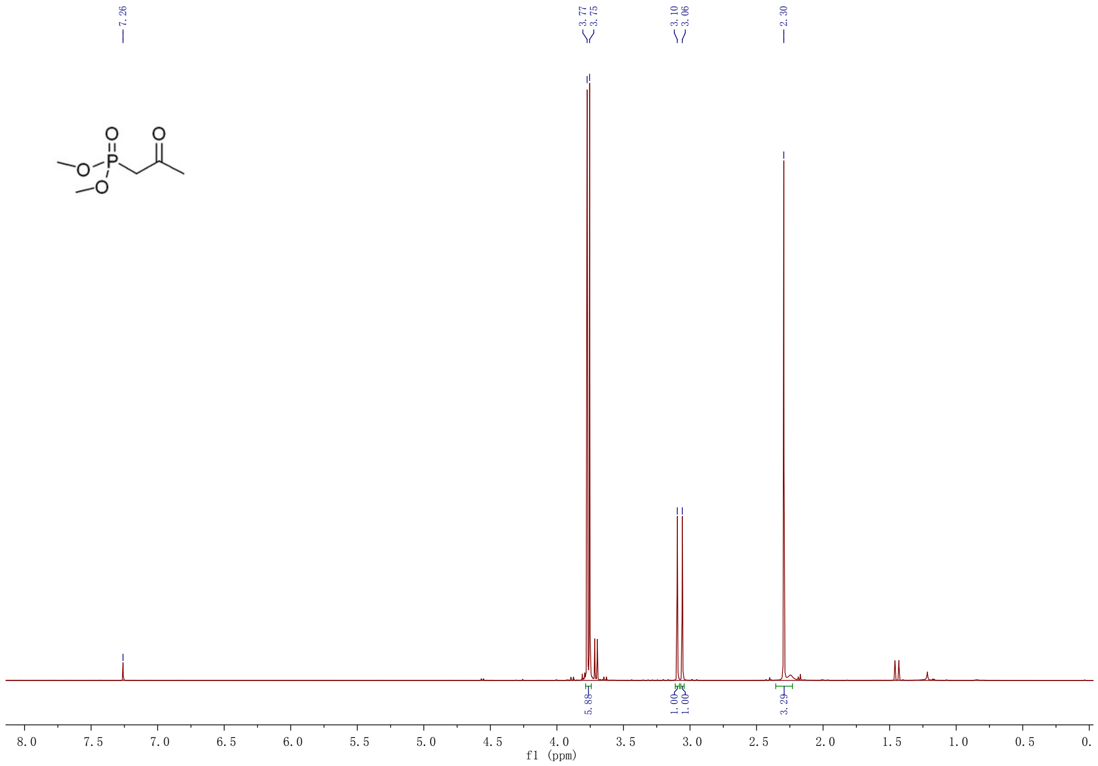

Fig.S 61 ^{1}H NMR of compound **4a**

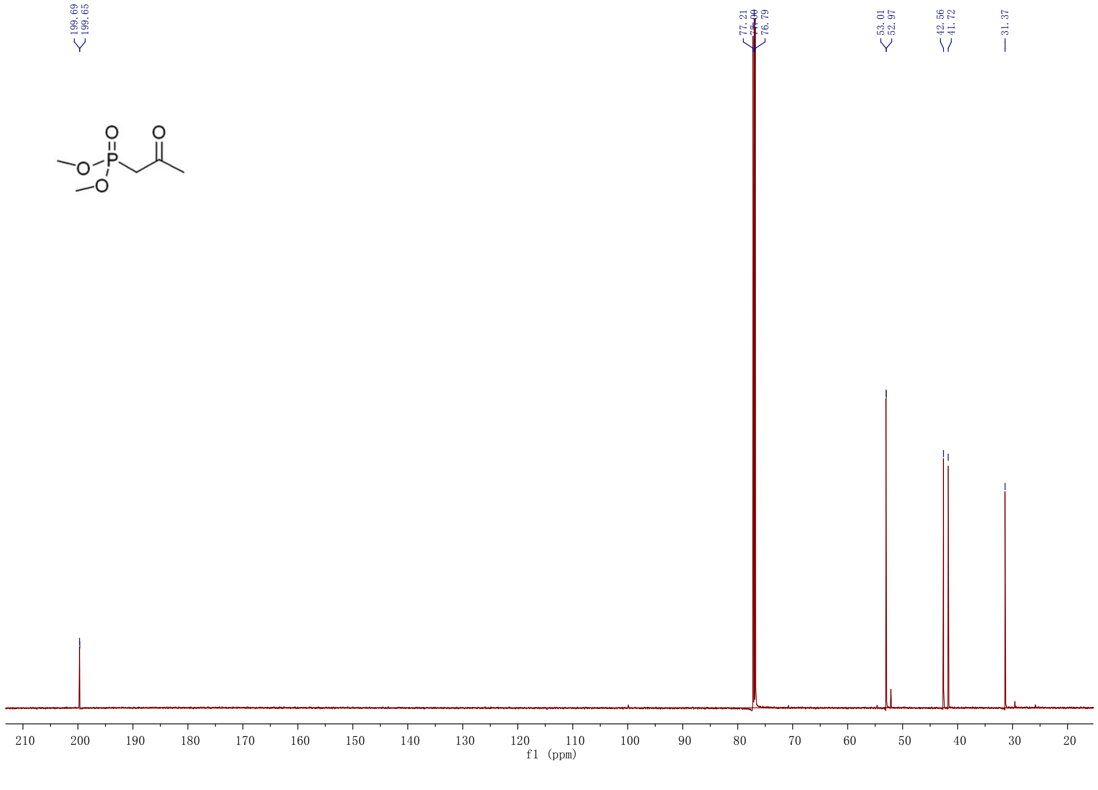

Fig.S 62 ^{13}C NMR of compound **4a**

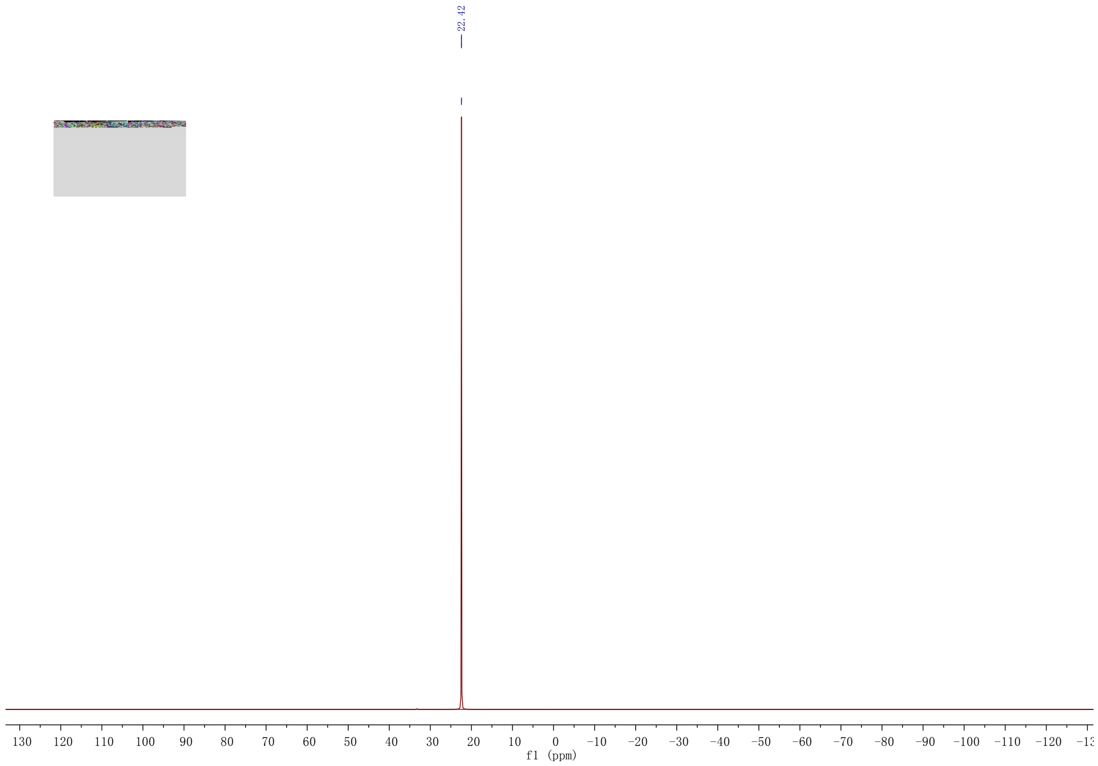

Fig.S 63 ^{31}P NMR of compound **4a**

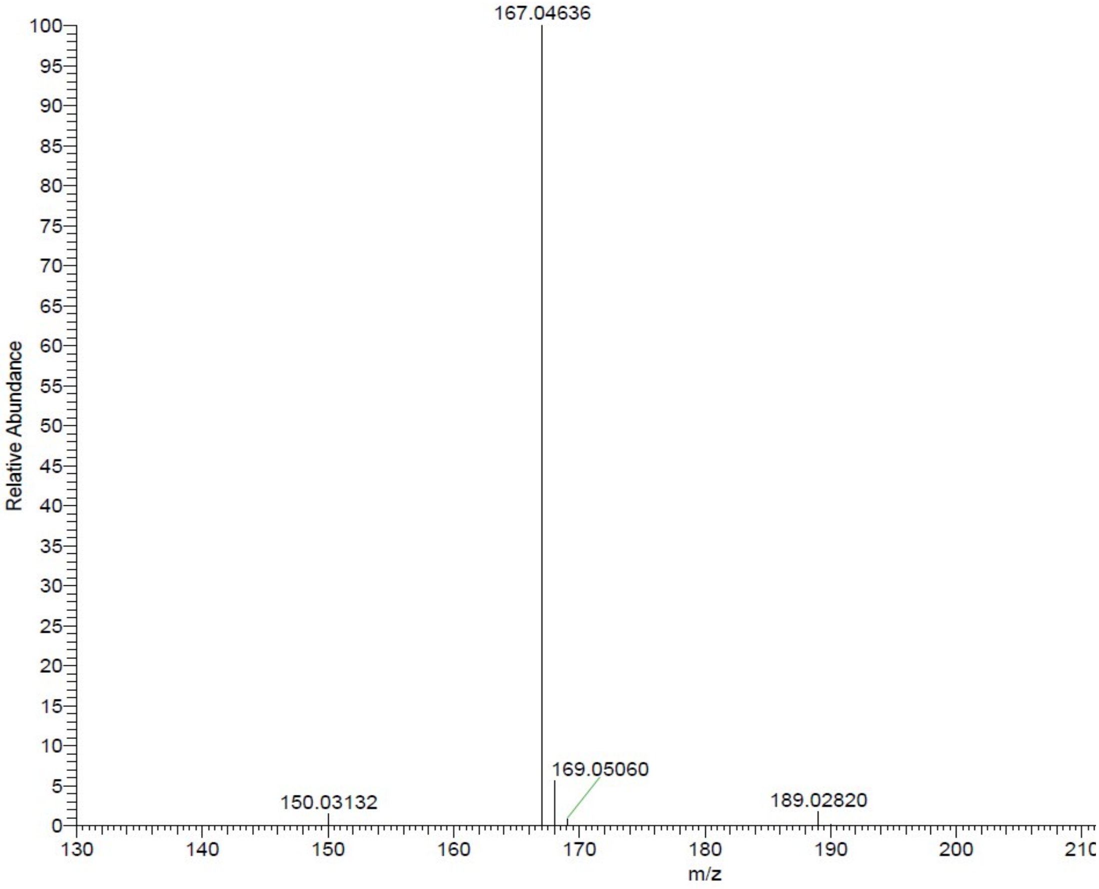

Fig.S 64 HRMS of compound **4a**

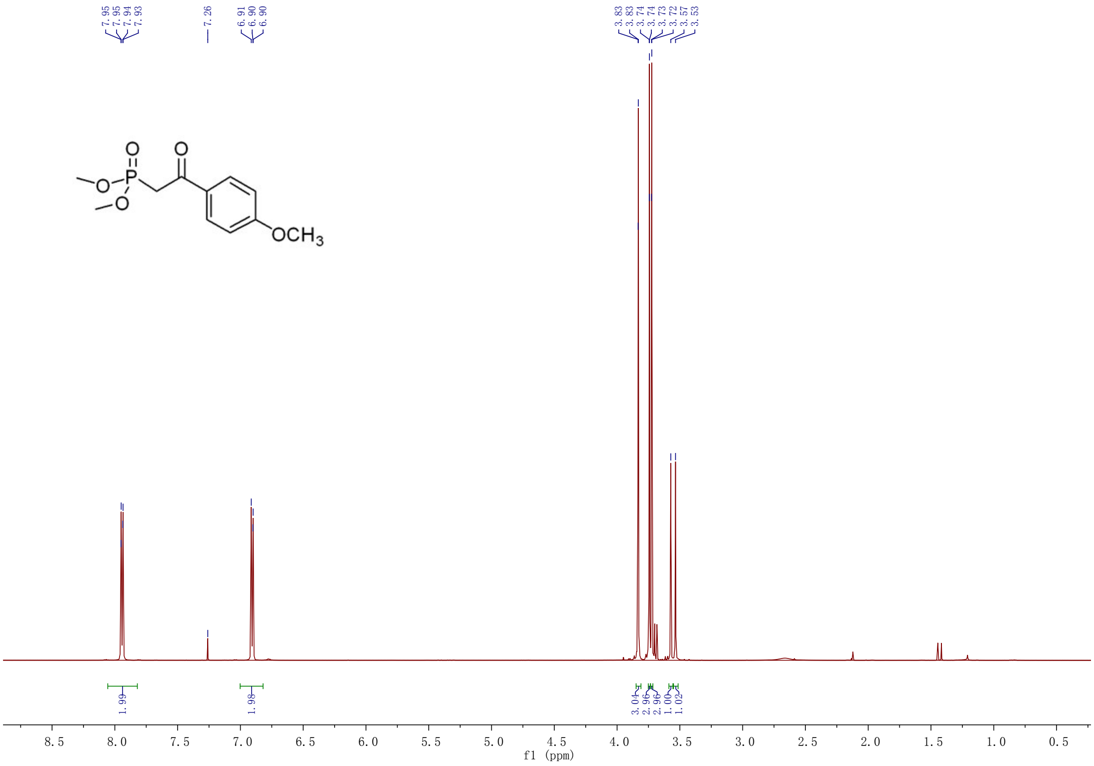

Fig.S 65 ^{1}H NMR of compound **4b**

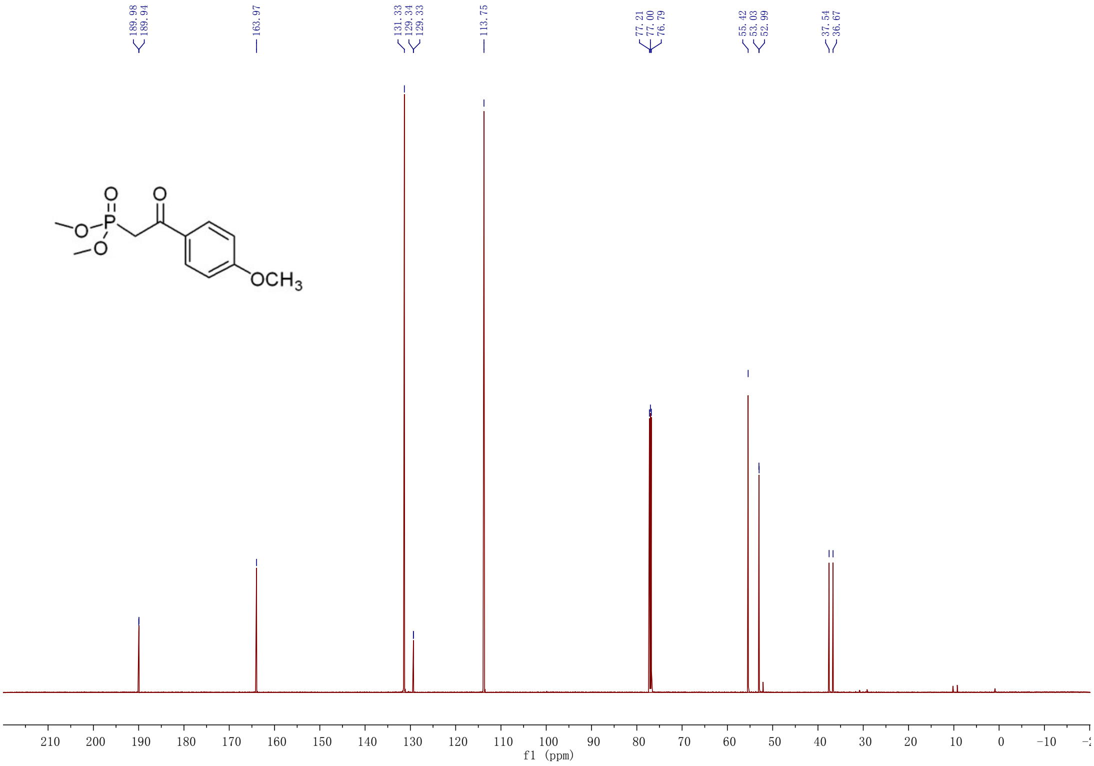

Fig.S 66 ^{13}C NMR of compound **4b**

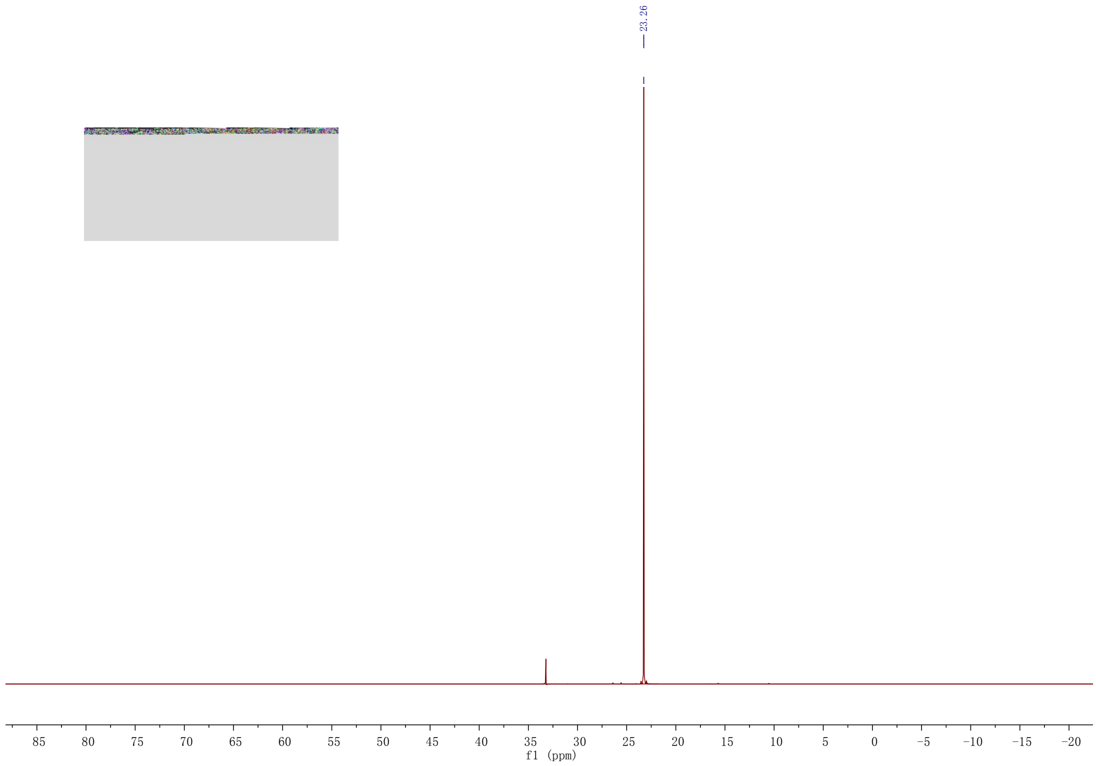

Fig.S 67 ^{31}P NMR of compound **4b**

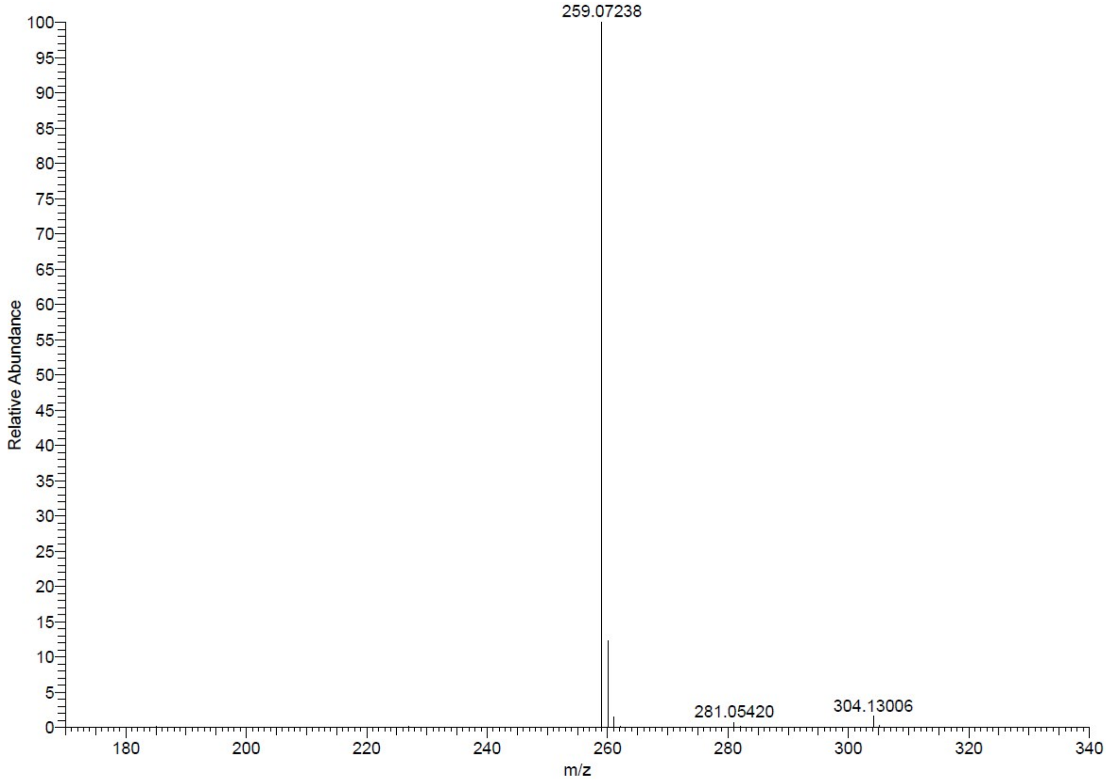

Fig.S 68 HRMS of compound **4b**

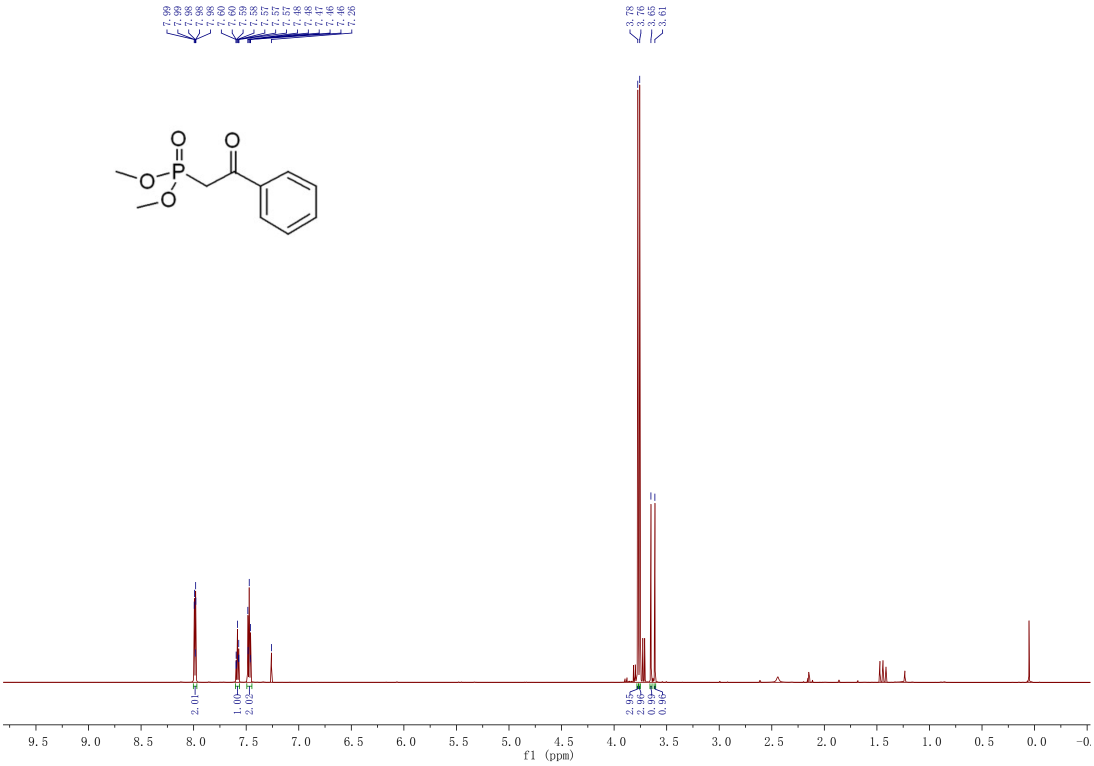

Fig.S 69 ^{1}H NMR of compound **4c**

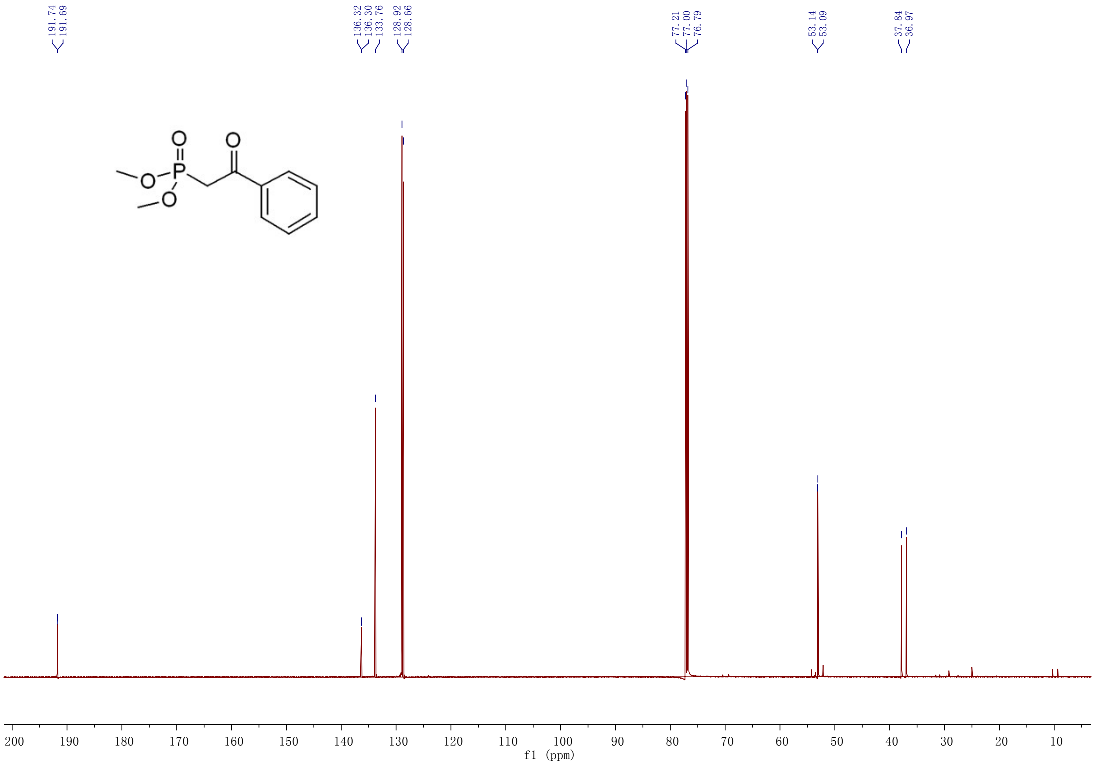

Fig.S 70 ^{13}C NMR of compound **4c**

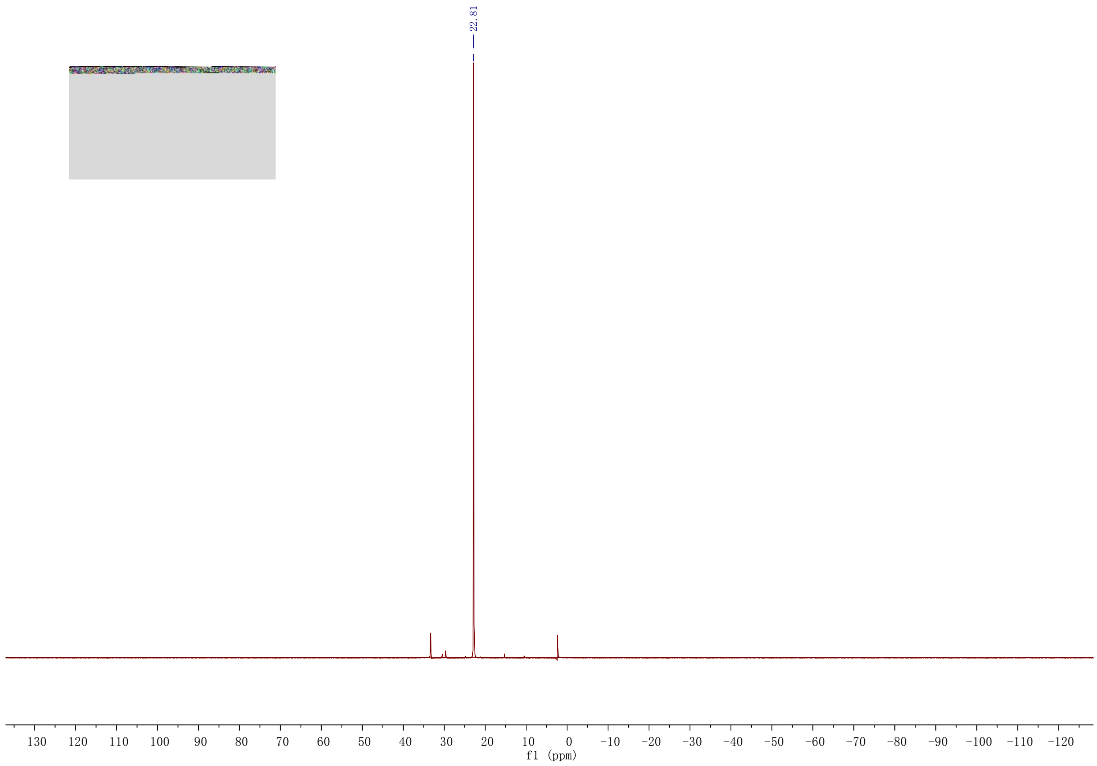

Fig.S 71 ^{31}P NMR of compound **4c**

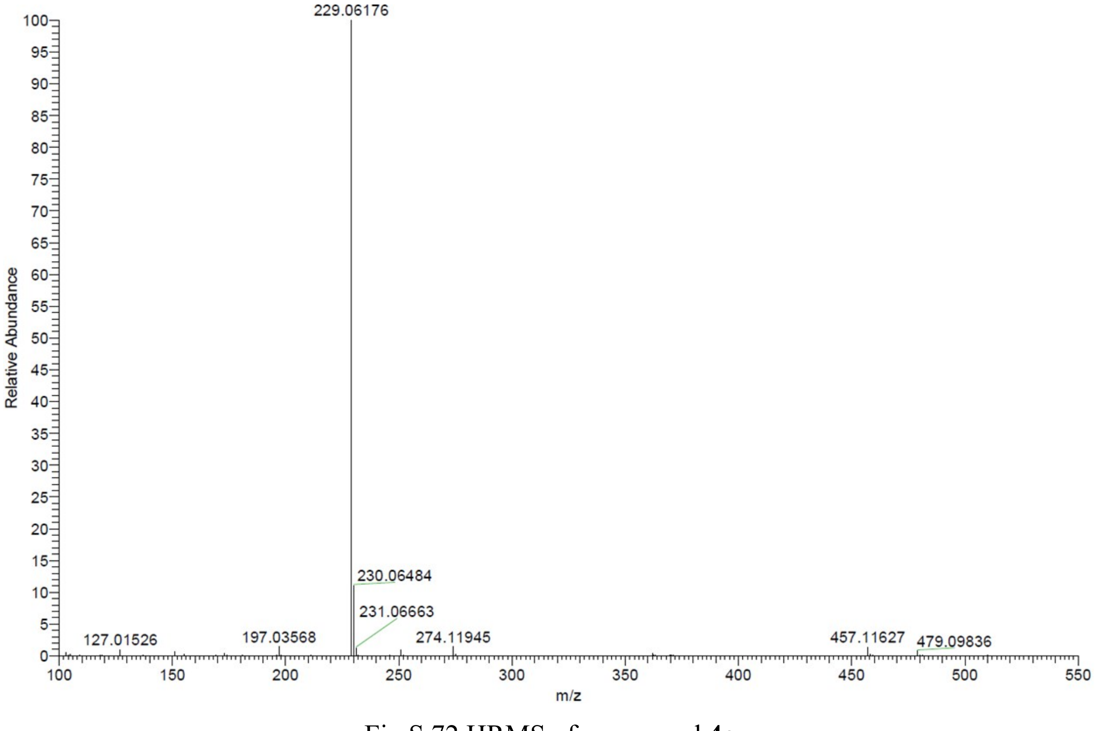

Fig.S 72 HRMS of compound **4c**

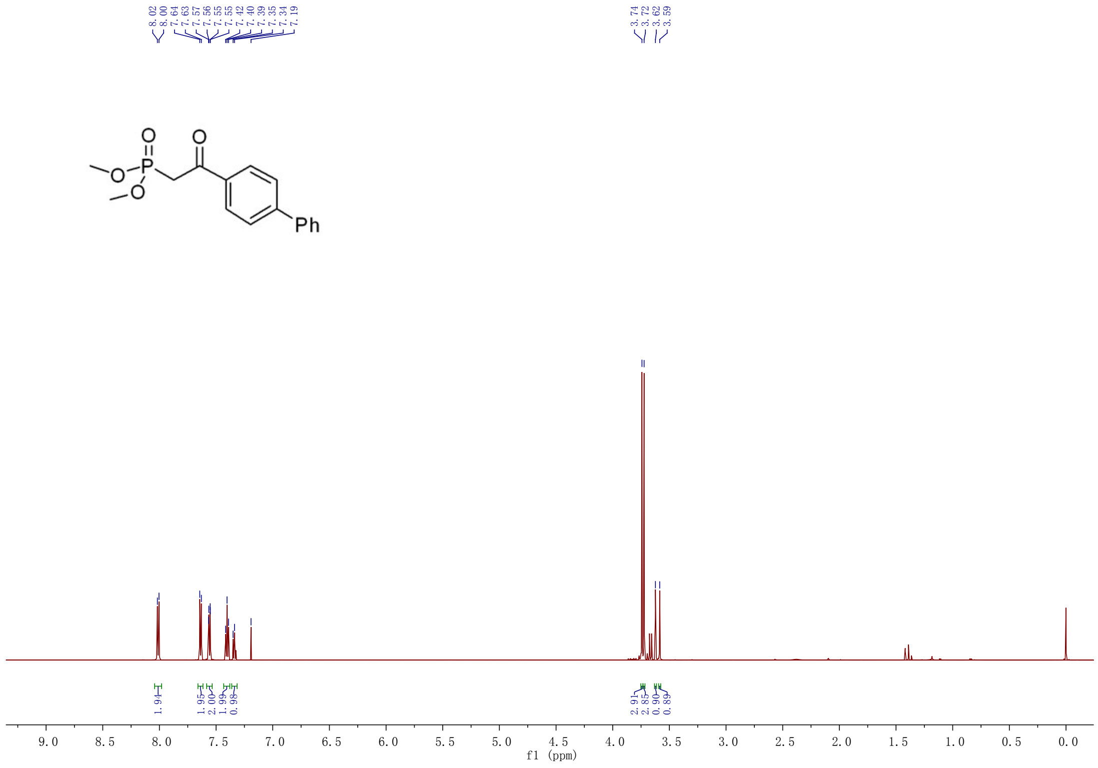

Fig.S 73 ^{1}H NMR of compound **4d**

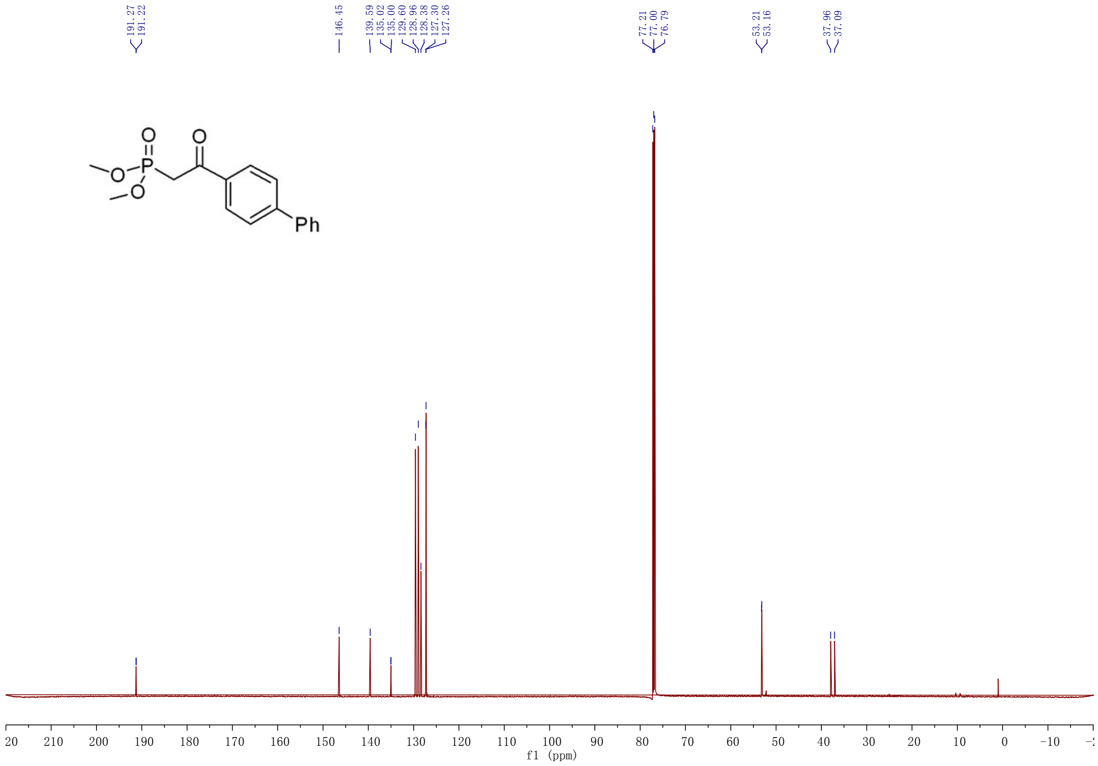

Fig.S 74 ^{13}C NMR of compound **4d**

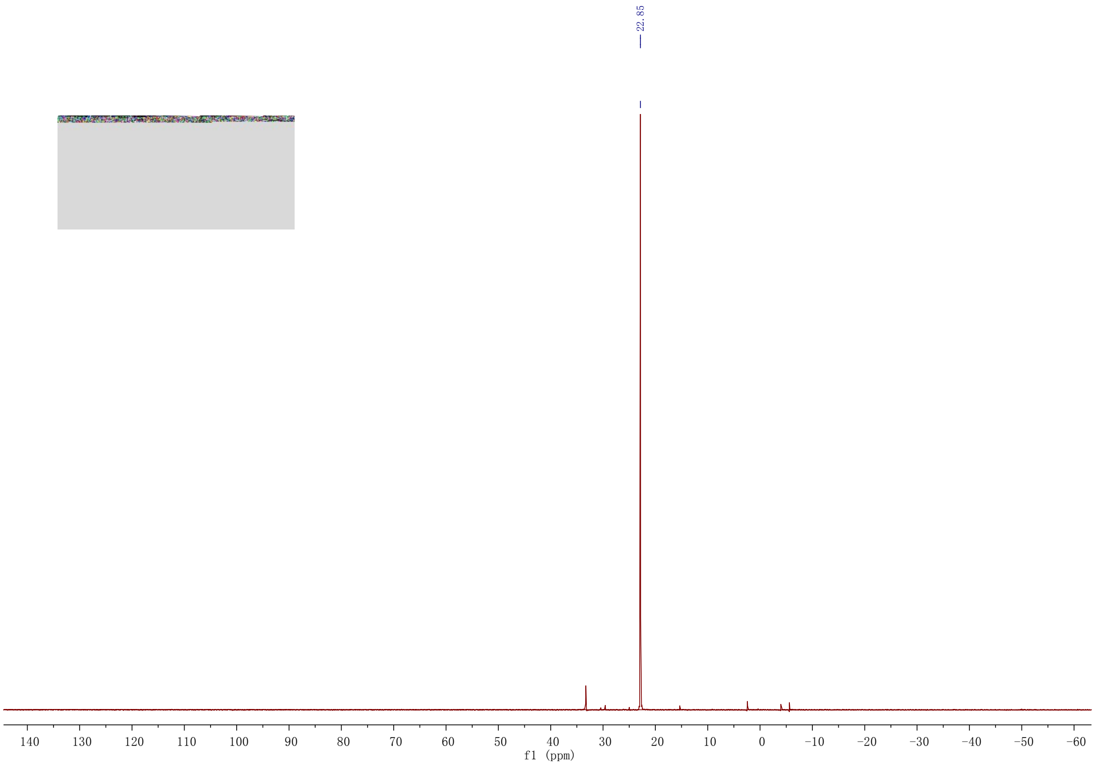

Fig.S 75 ^{31}P NMR of compound **4d**

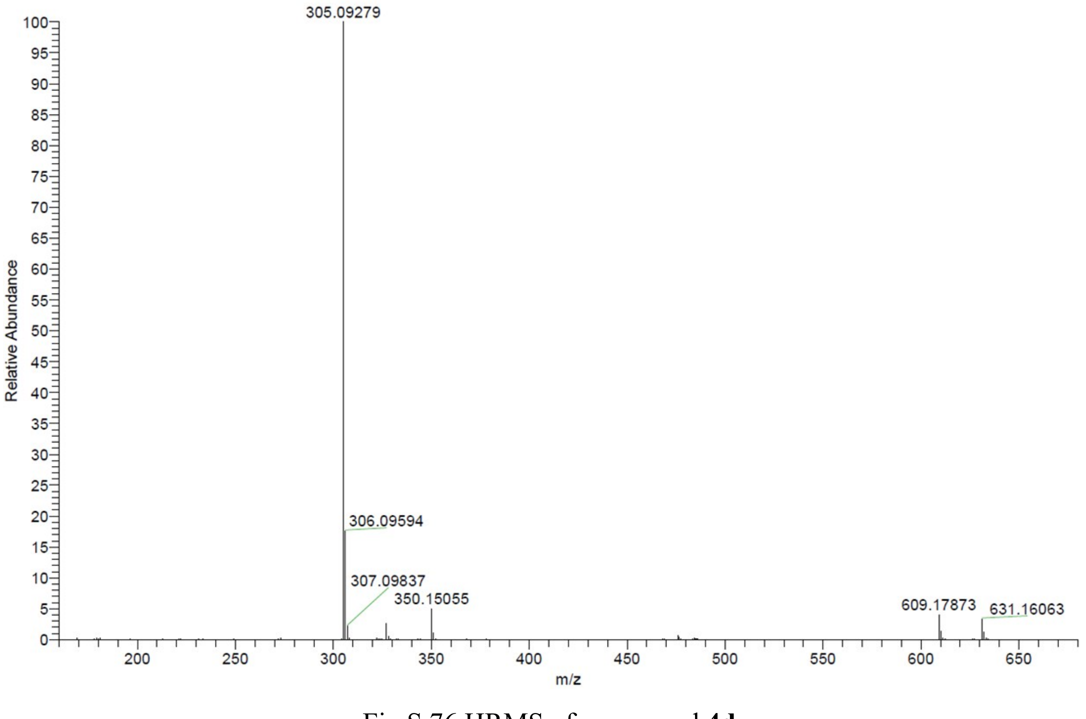

Fig.S 76 HRMS of compound **4d**

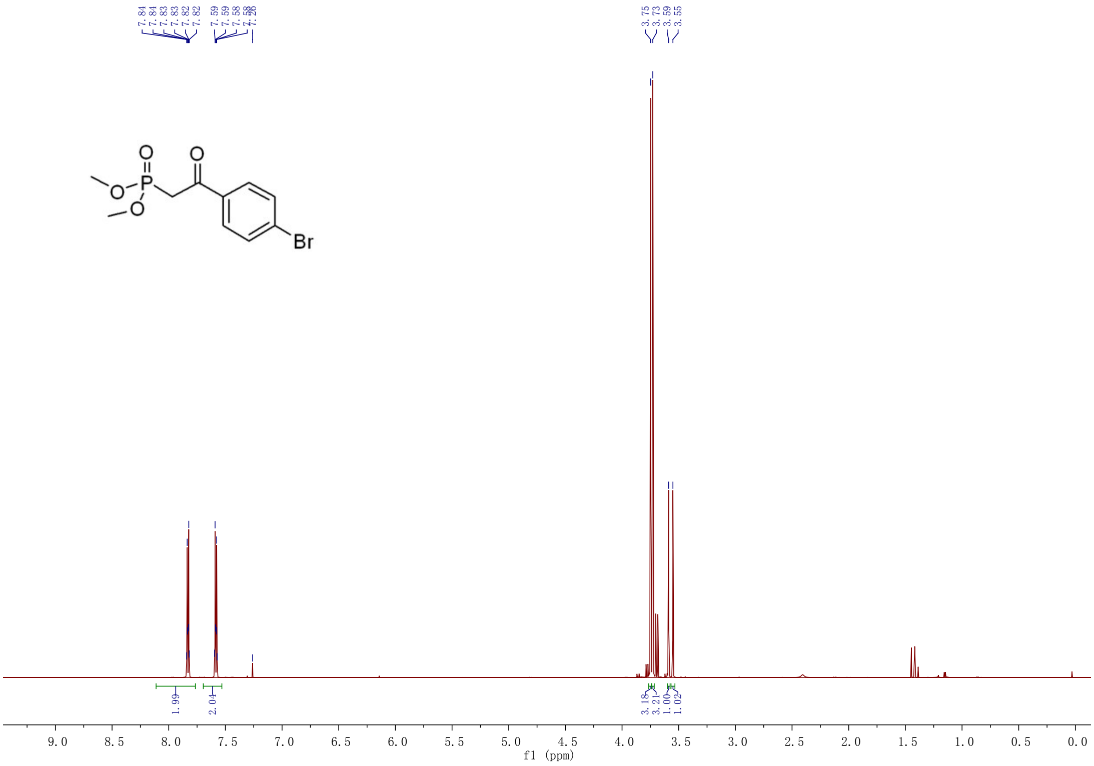

Fig.S 77 ^{1}H NMR of compound **4e**

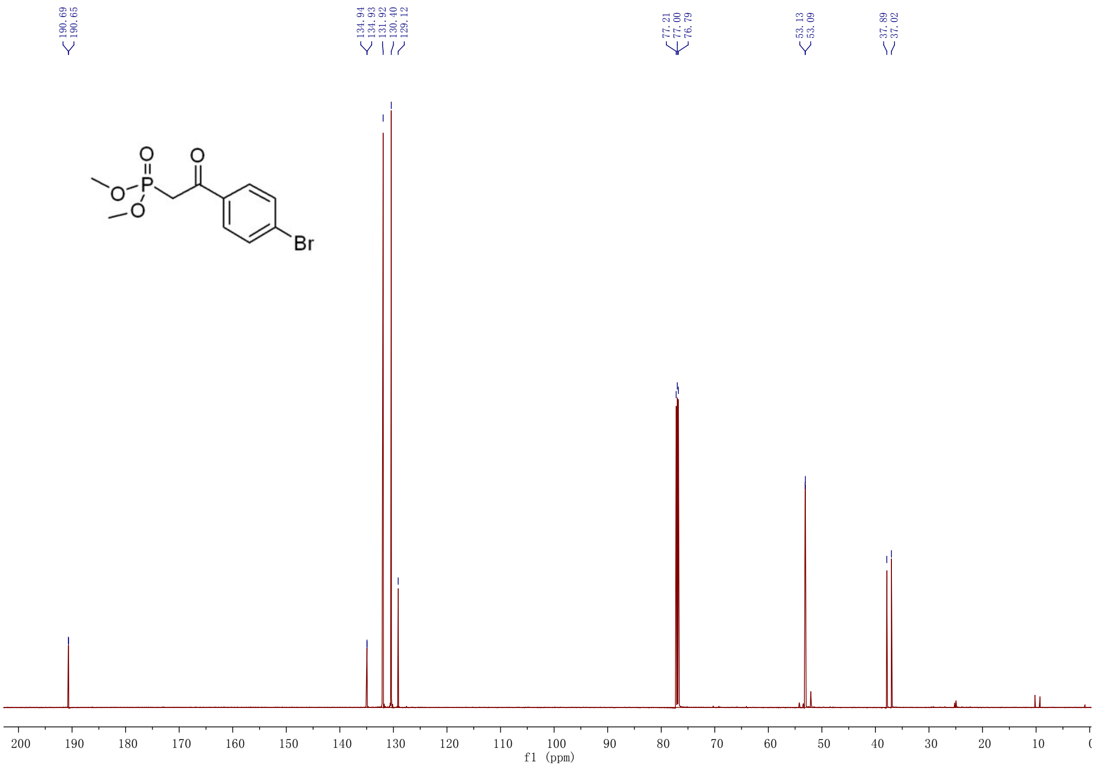

Fig.S 78 ^{13}C NMR of compound **4e**

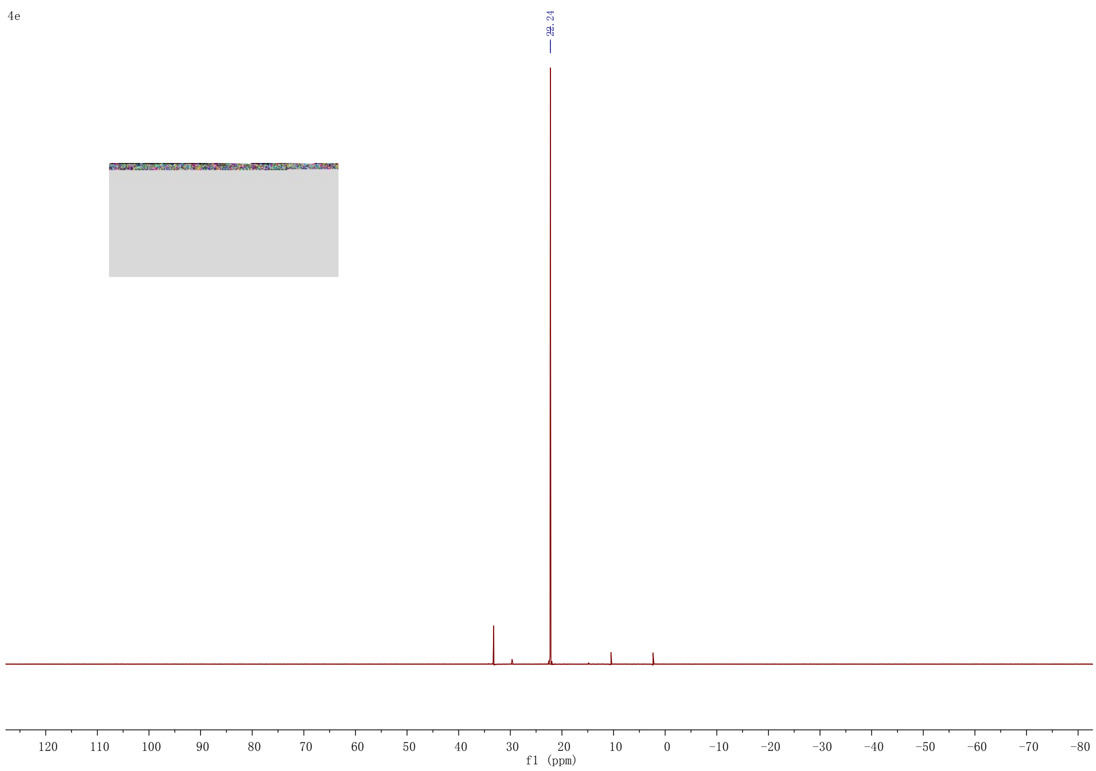

Fig.S 79 ^{31}P NMR of compound **4e**

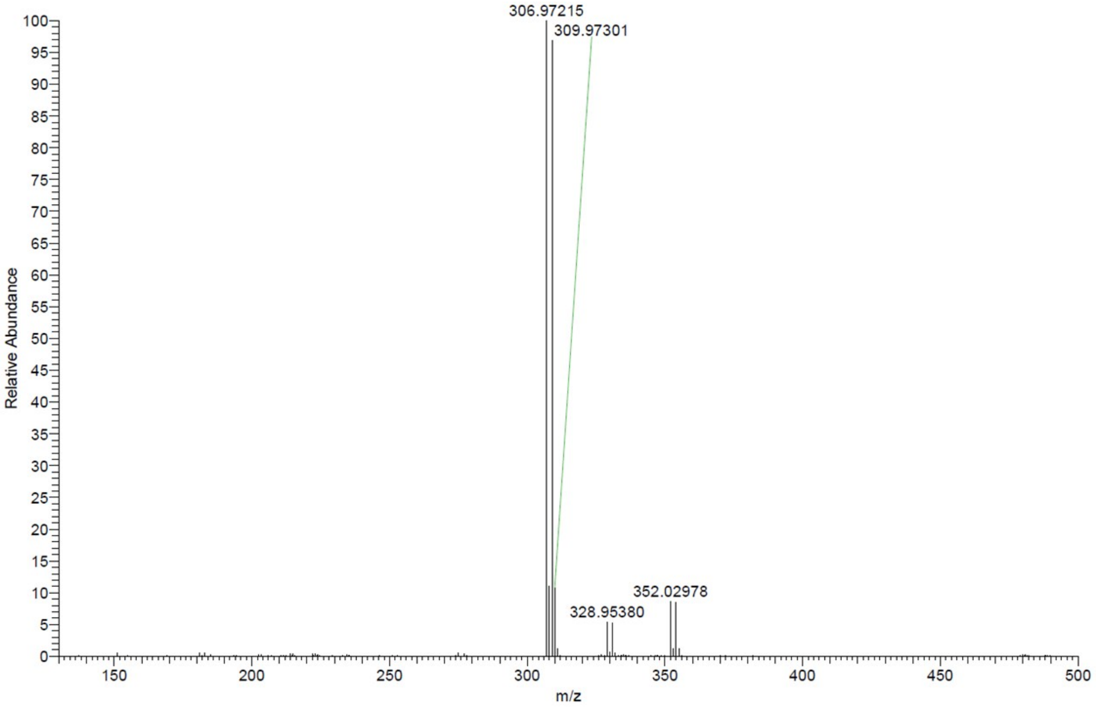

Fig.S 80 HRMS of compound **4e**

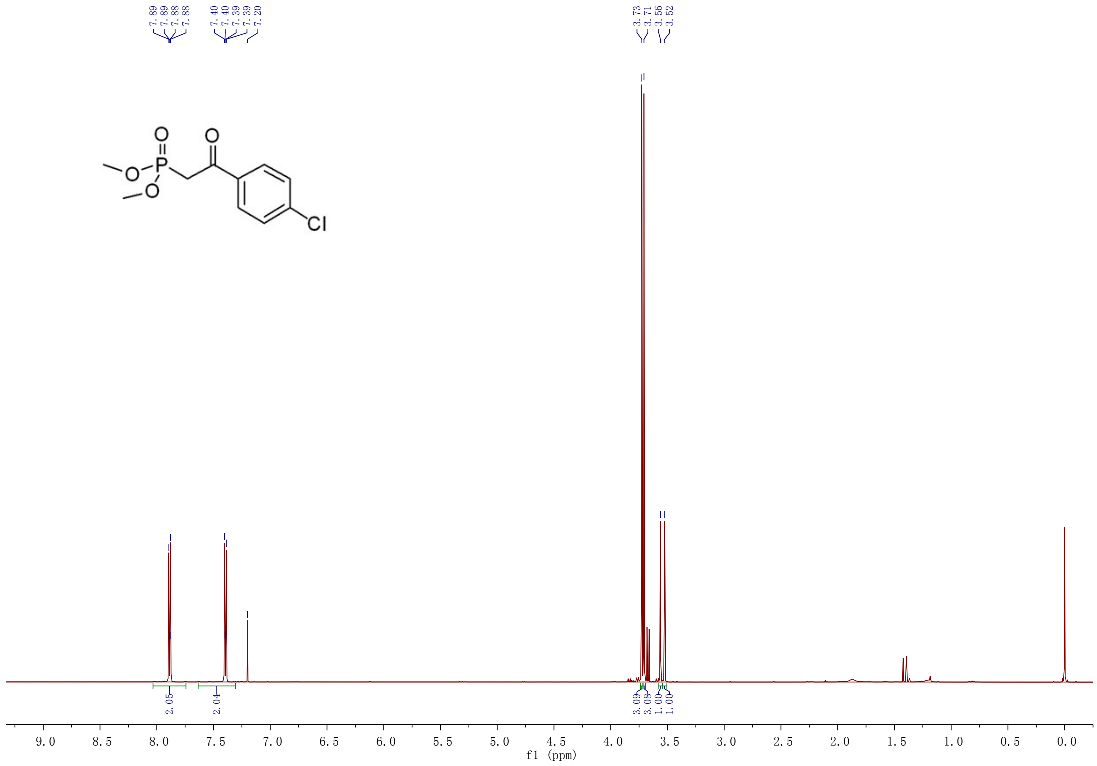

Fig.S 81 ^{1}H NMR of compound **4f**

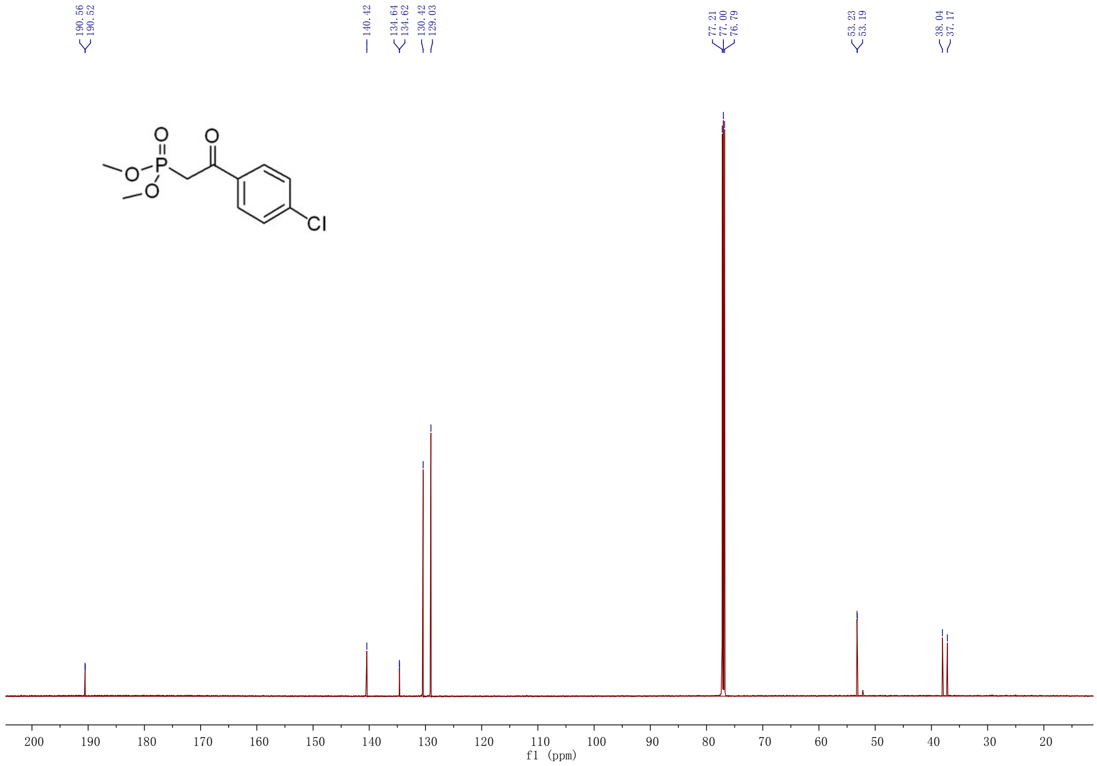

Fig.S 82 ^{13}C NMR of compound **4f**

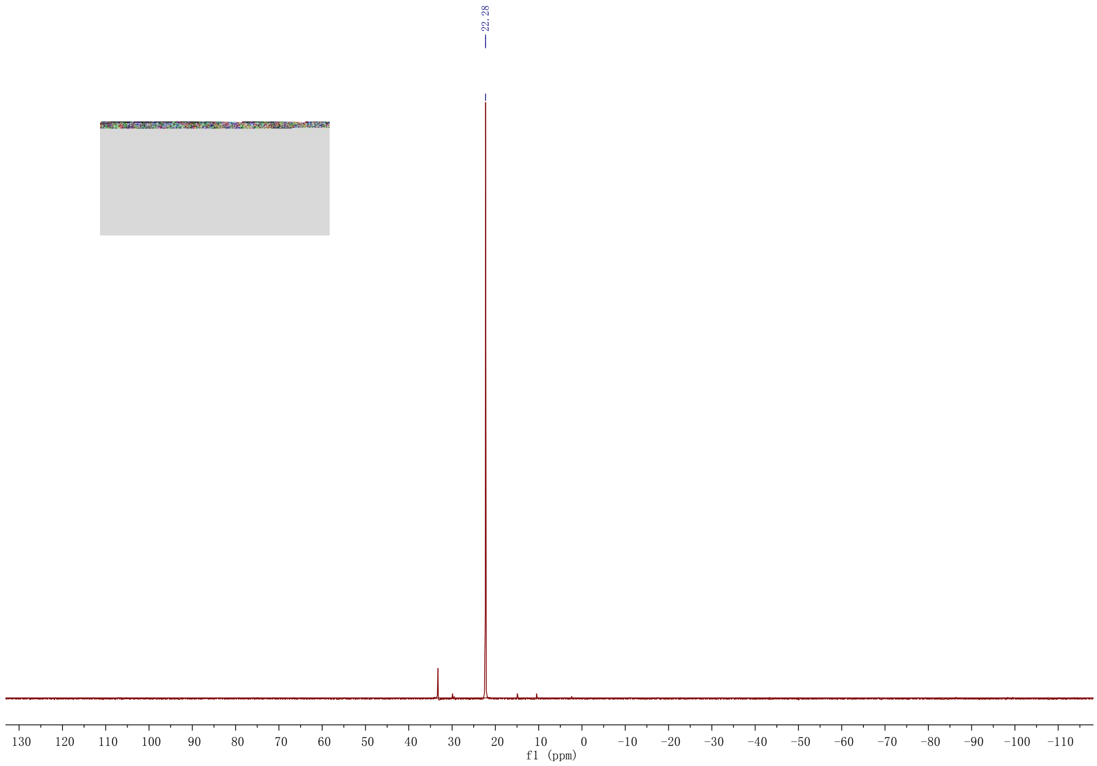

Fig.S 83 ^{31}P NMR of compound **4f**

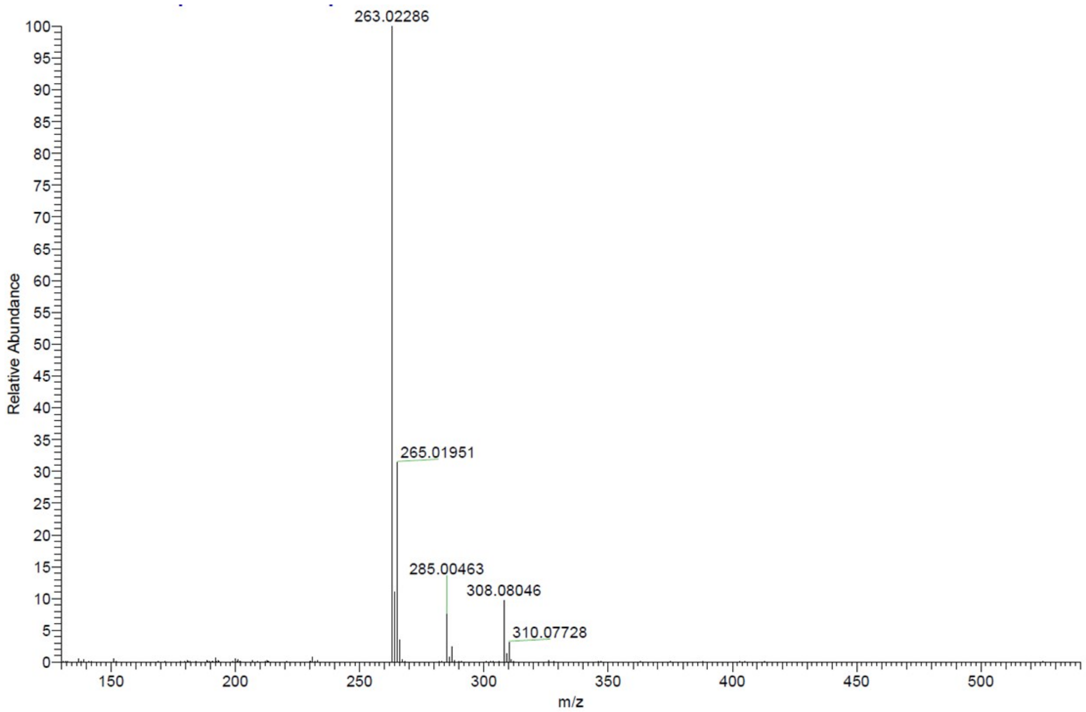

Fig.S 84 HRMS of compound **4f**

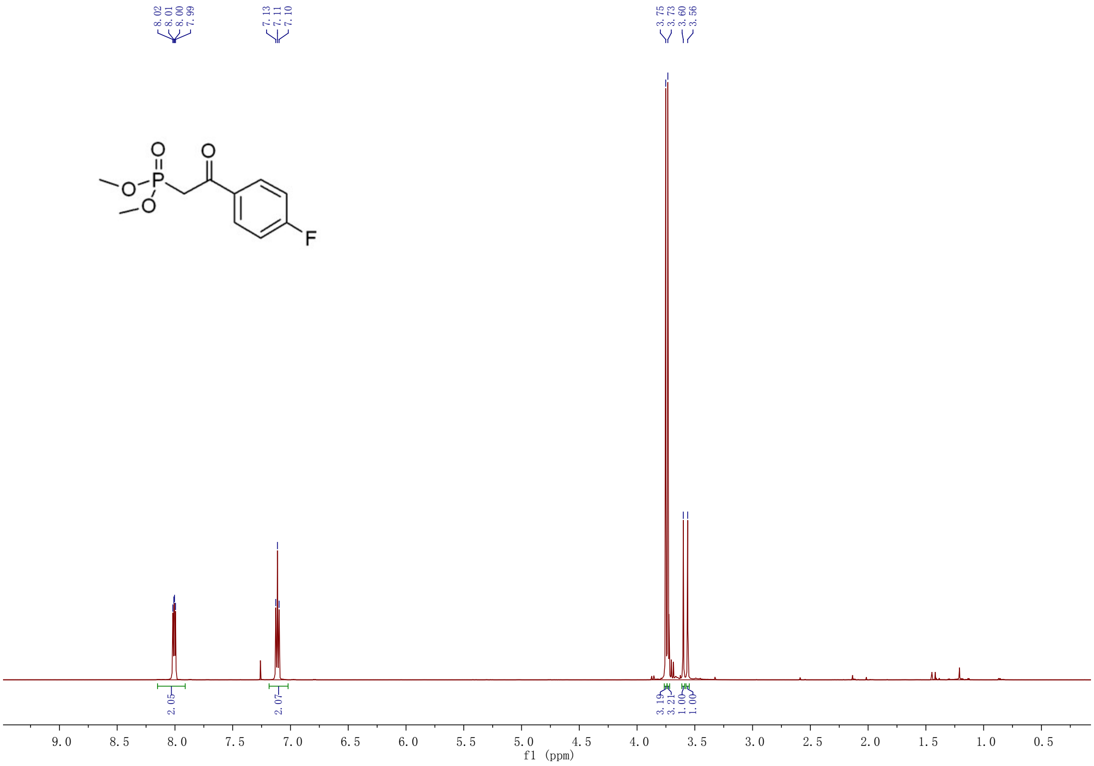

Fig.S 85 ^{1}H NMR of compound **4g**

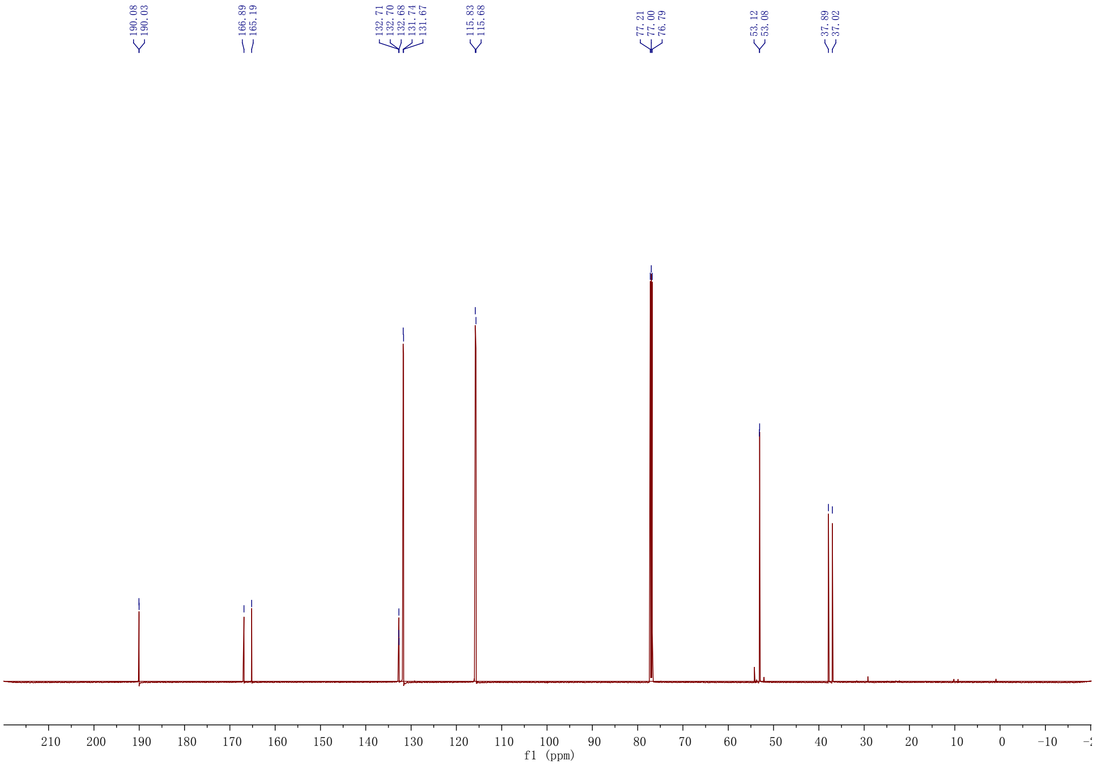

Fig.S 86 ^{13}C NMR of compound **4g**

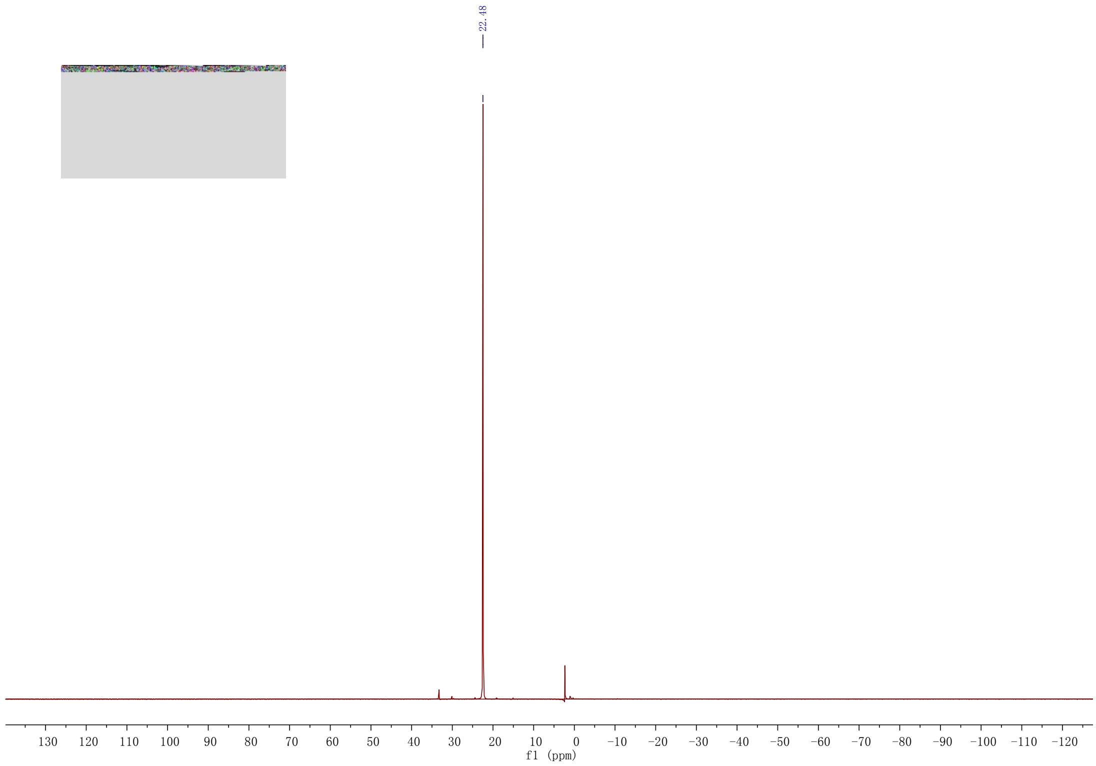

Fig.S 87 ^{31}P NMR of compound **4g**

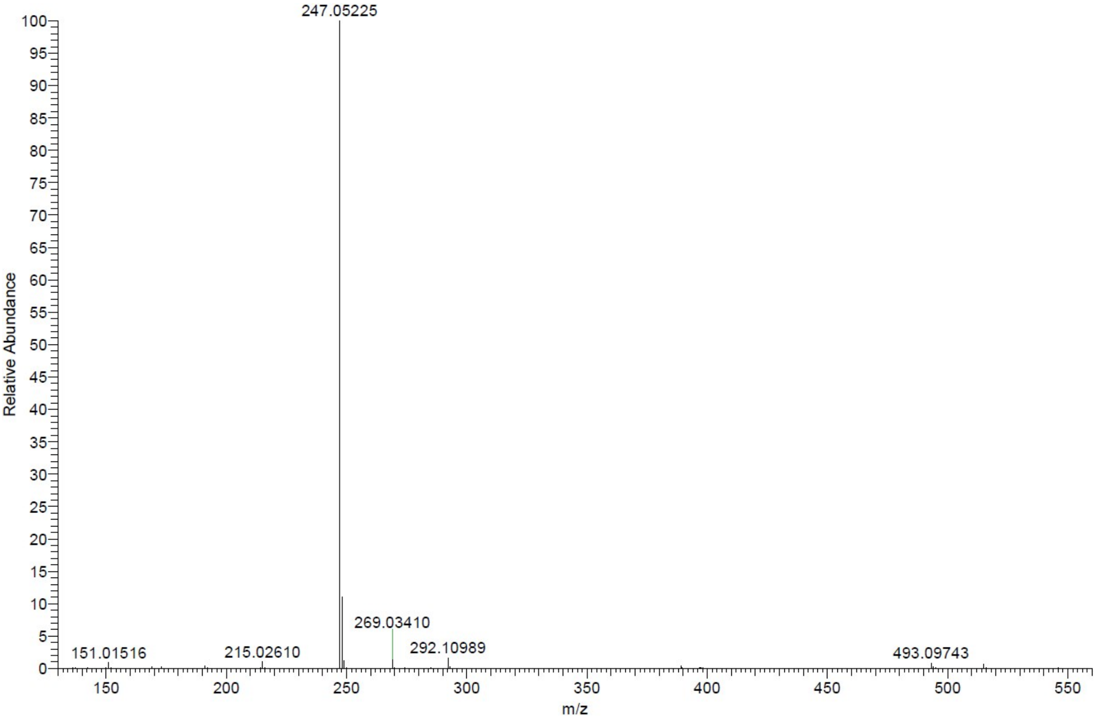

Fig.S 88 HRMS of compound **4g**

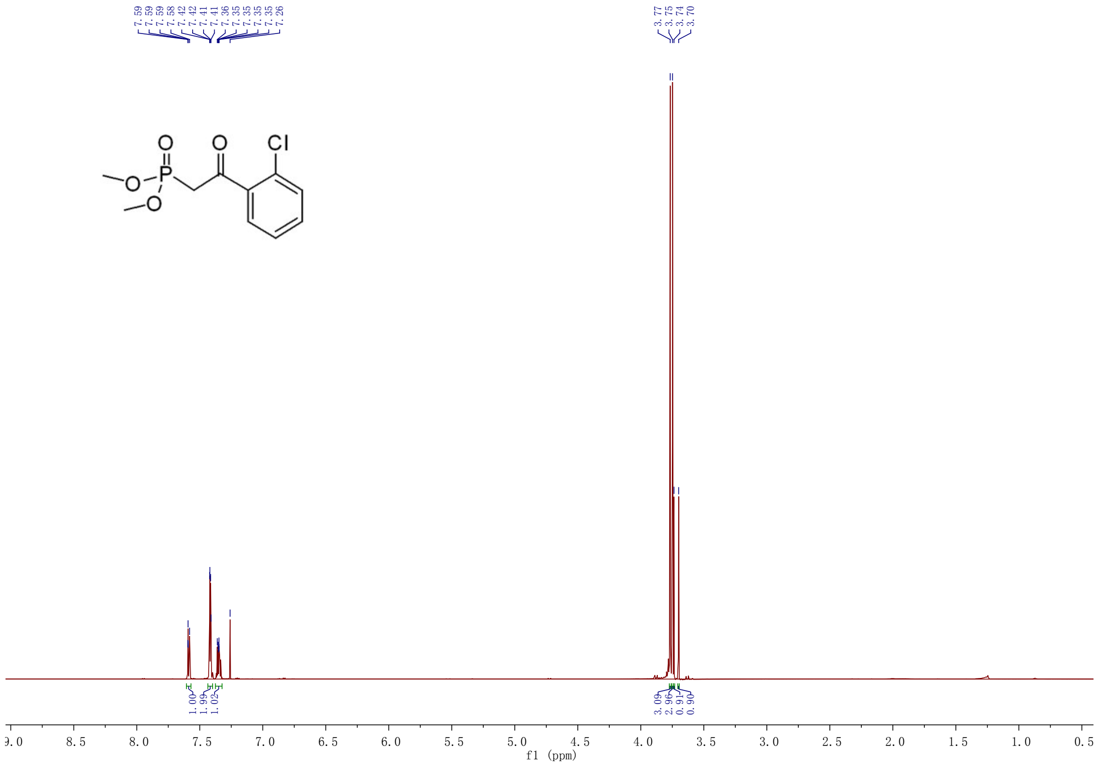

Fig.S 89 ^{1}H NMR of compound **4h**

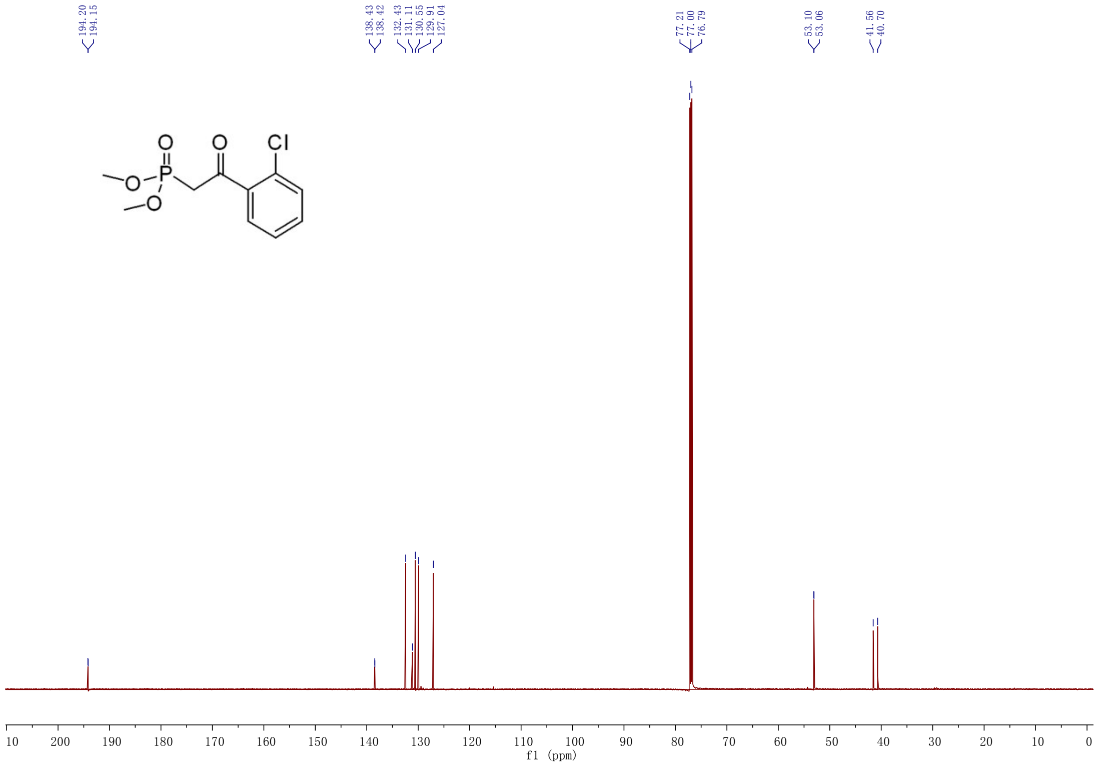

Fig.S 90 ^{13}C NMR of compound **4h**

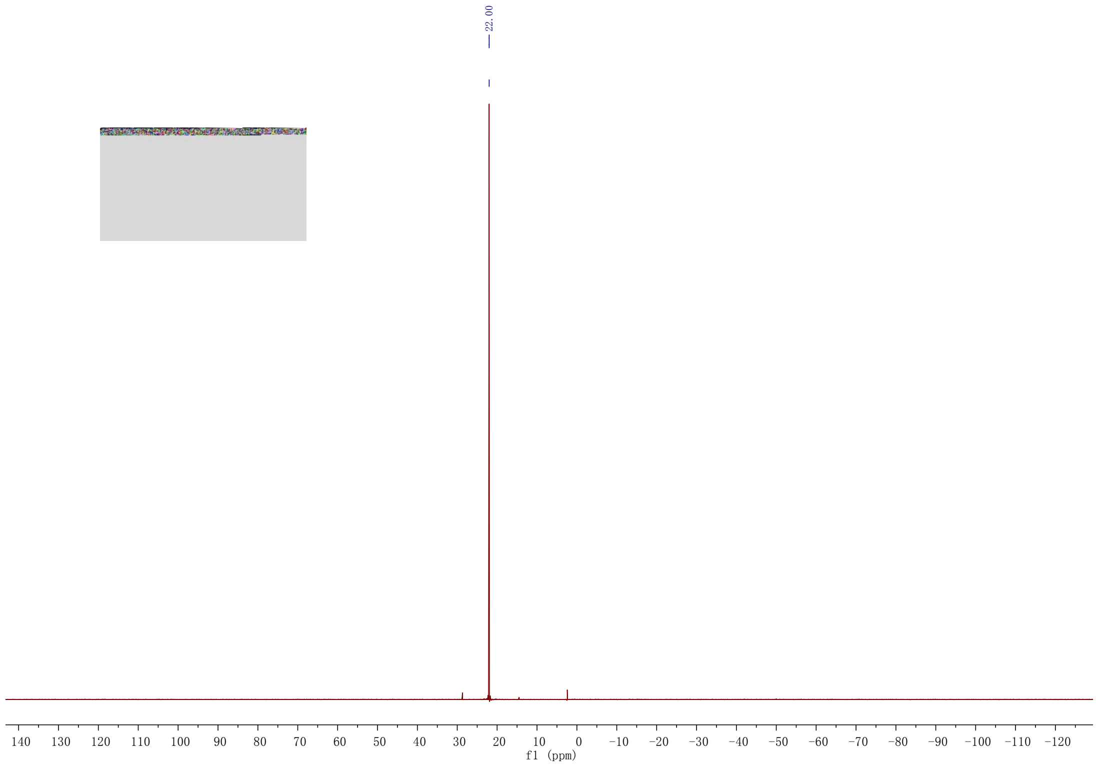

Fig.S 91 ^{31}P NMR of compound **4h**

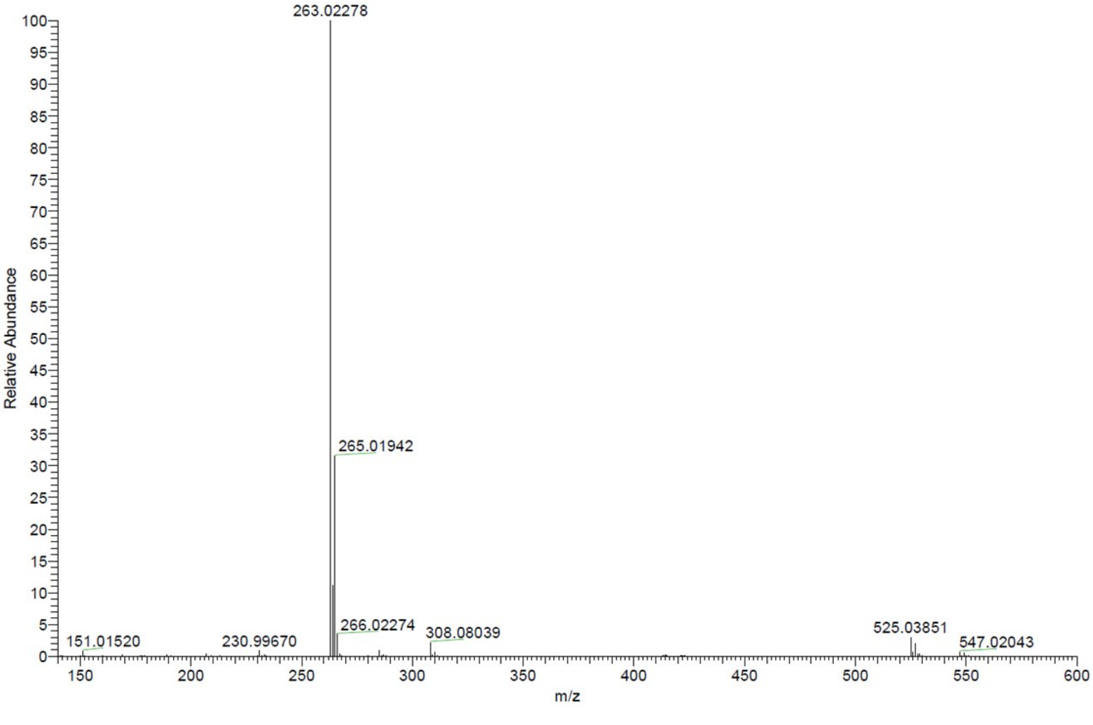

Fig.S 92 HRMS of compound **4h**

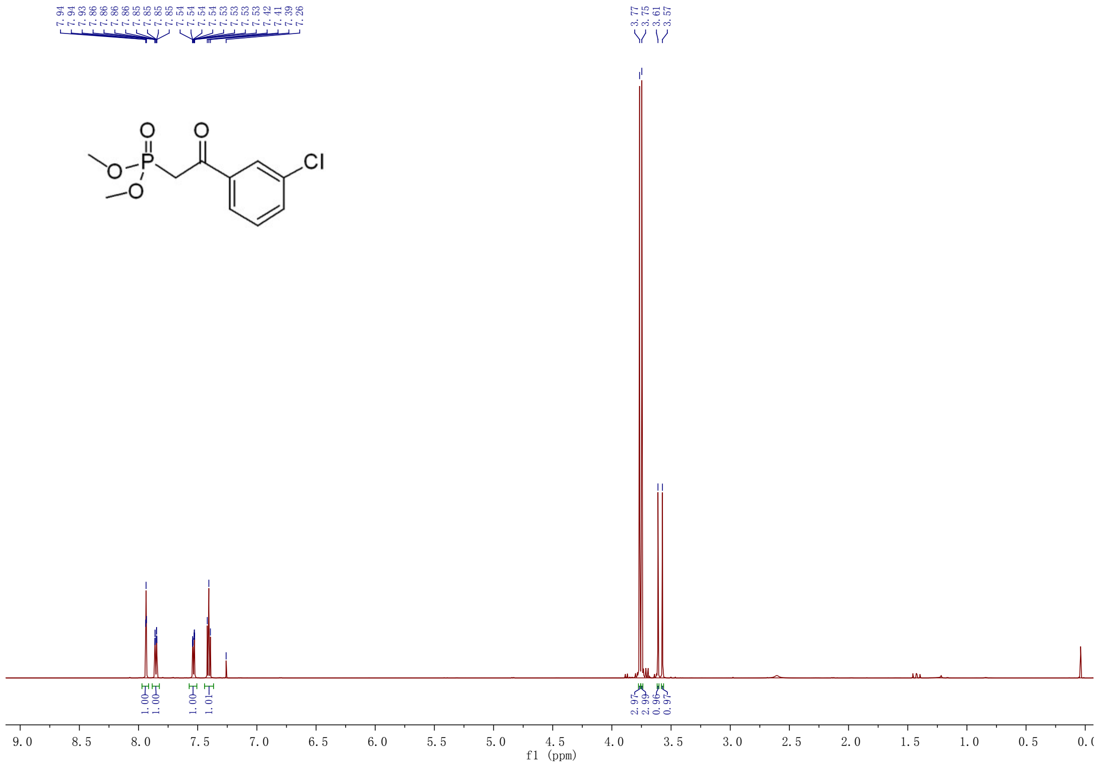

Fig.S 93 ^{1}H NMR of compound **4i**

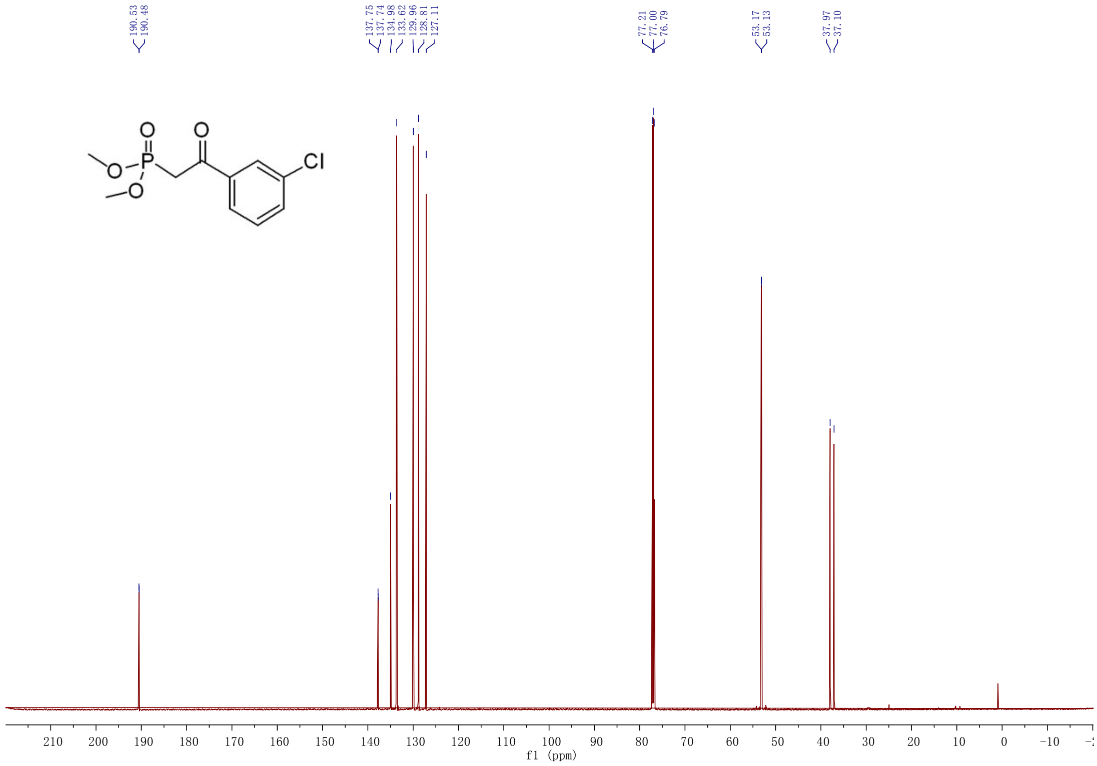

Fig.S 94 ^{13}C NMR of compound **4i**

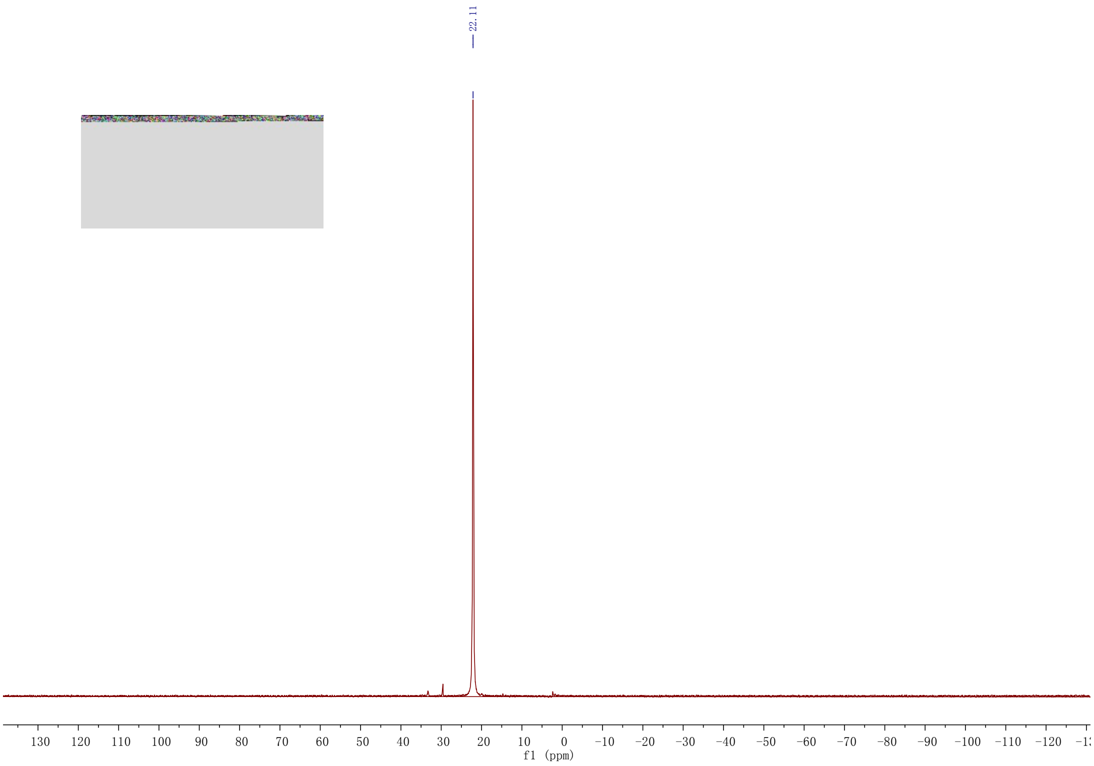

Fig.S 95 ^{31}P NMR of compound **4i**

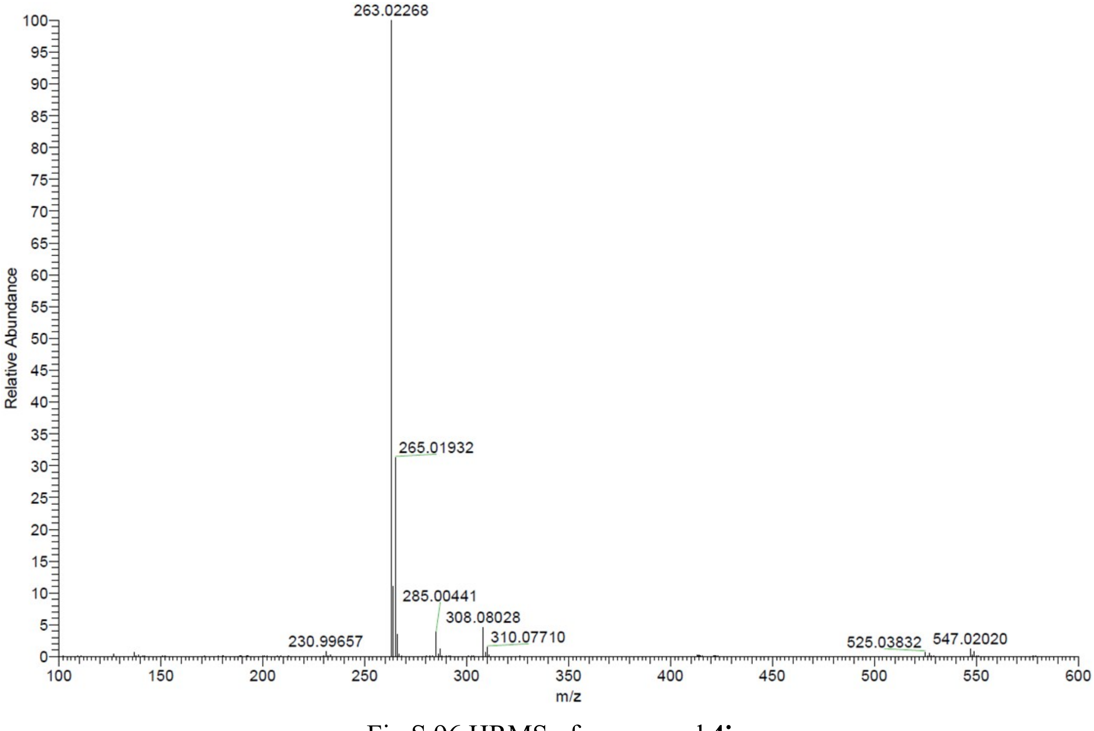

Fig.S 96 HRMS of compound **4i**

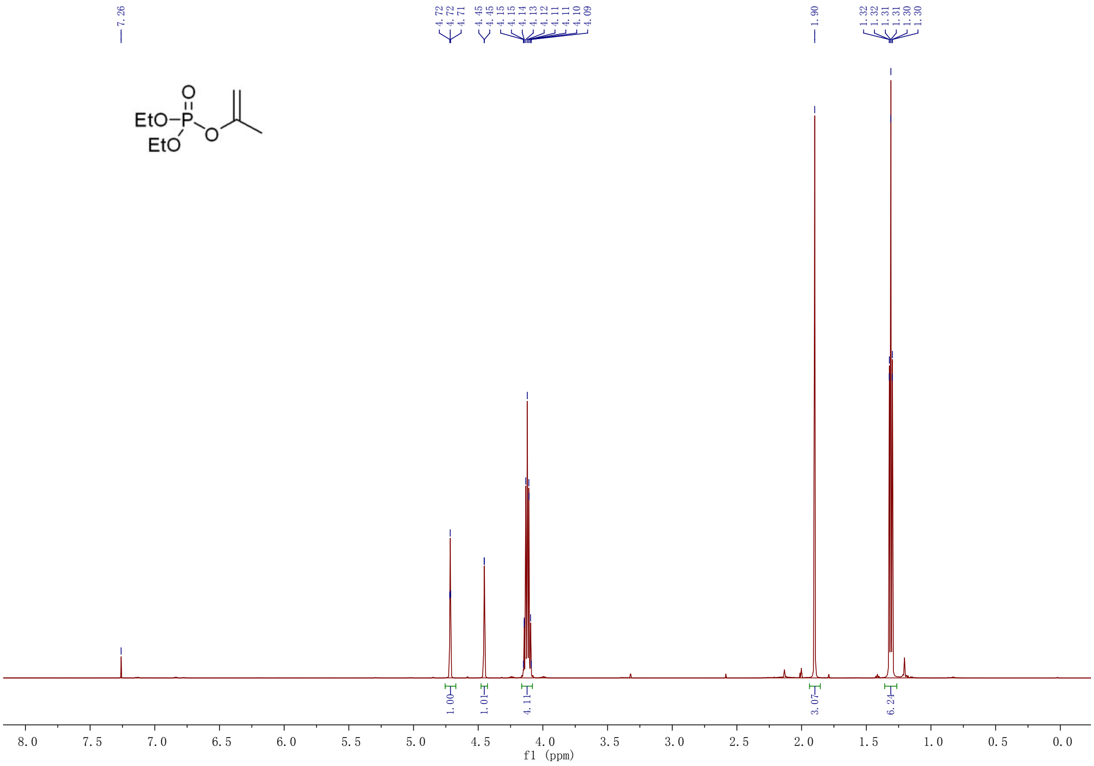

Fig.S 97 ^{1}H NMR of compound **5a**

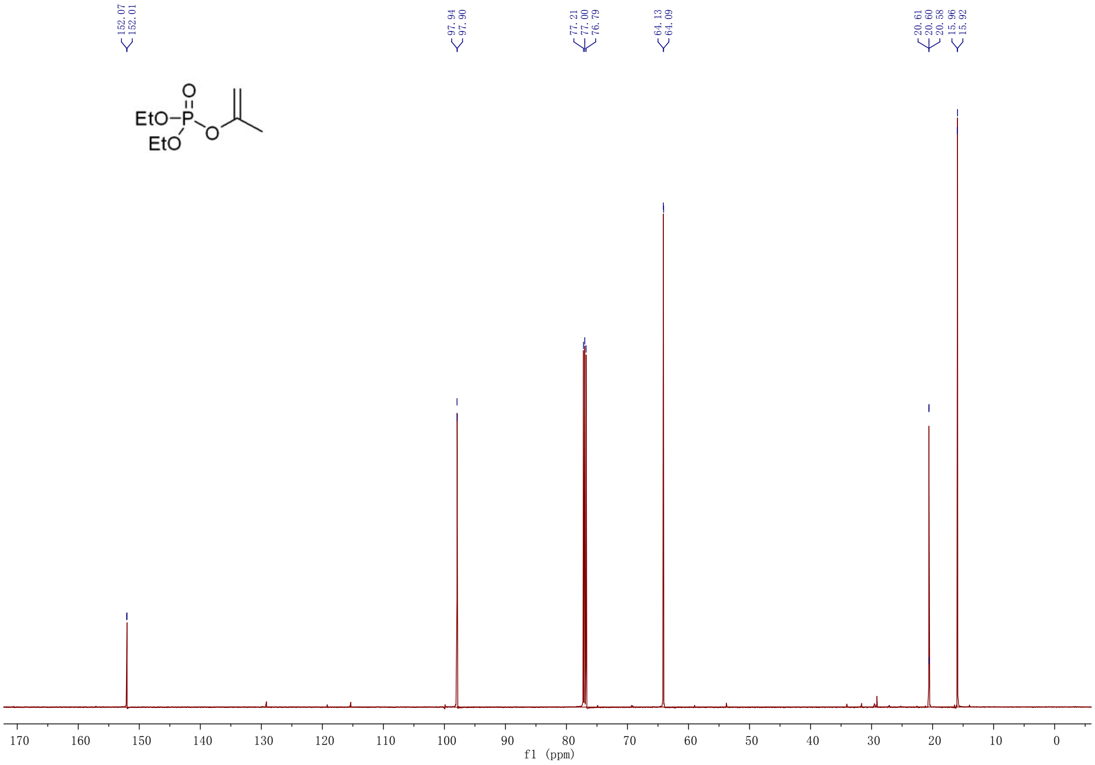

Fig.S 98 ^{13}C NMR of compound **5a**

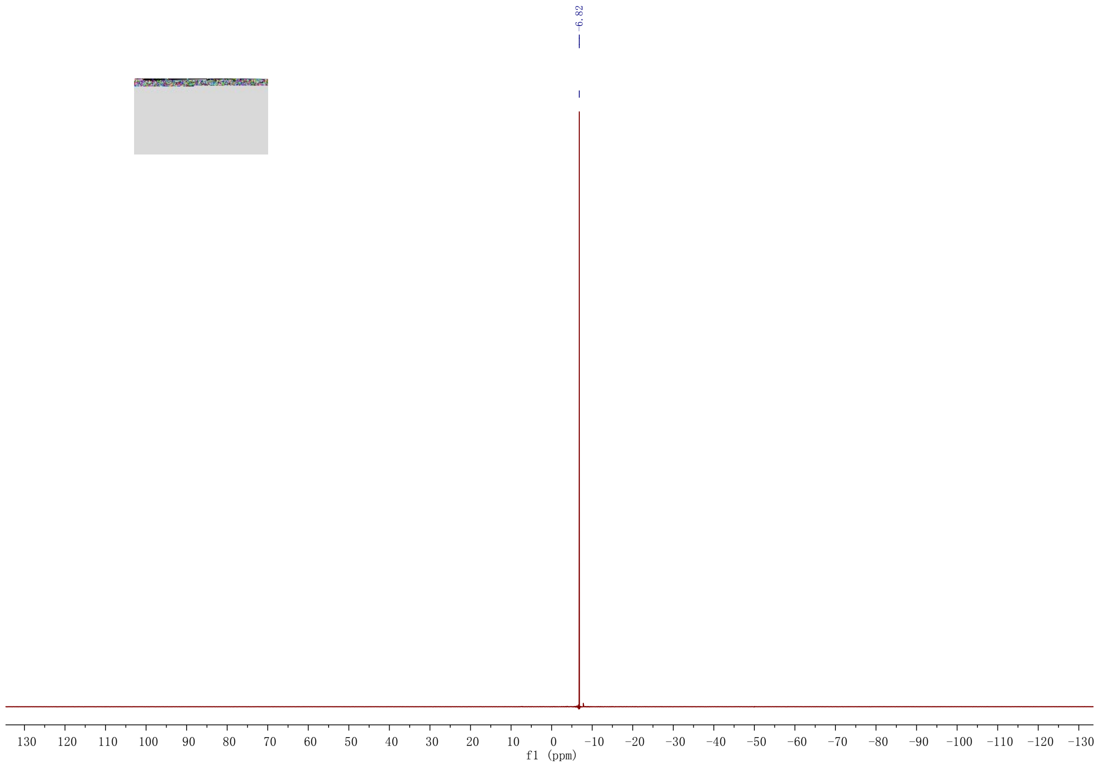

Fig.S 99 ^{31}P NMR of compound **5a**

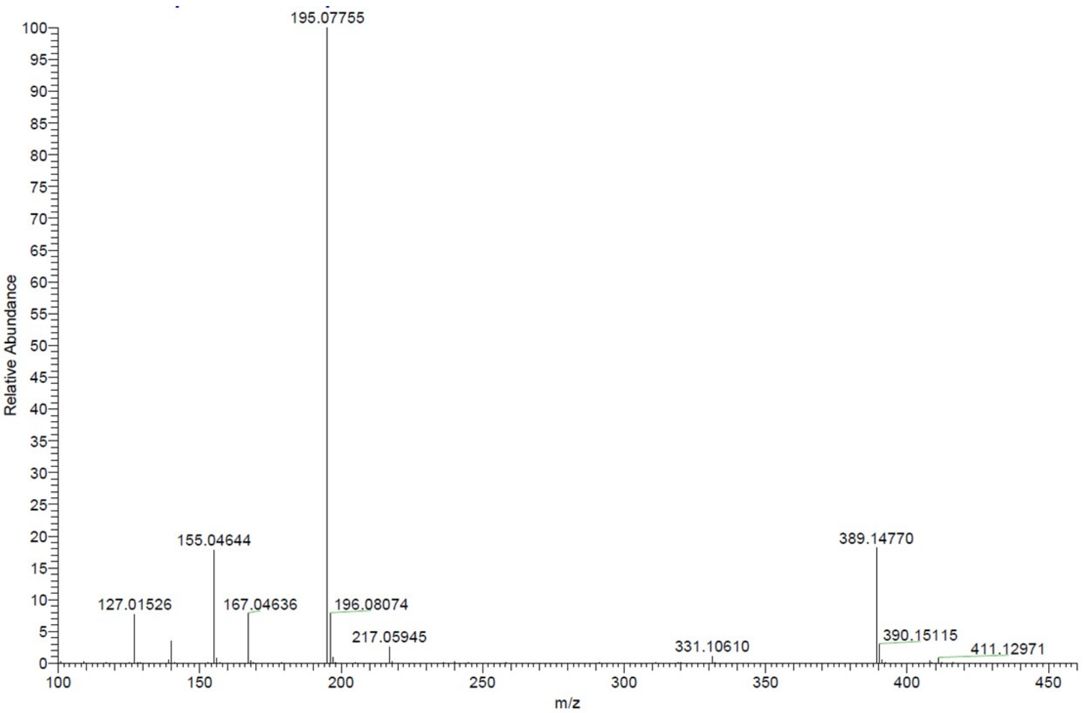

Fig.S 100 HRMS of compound **5a**

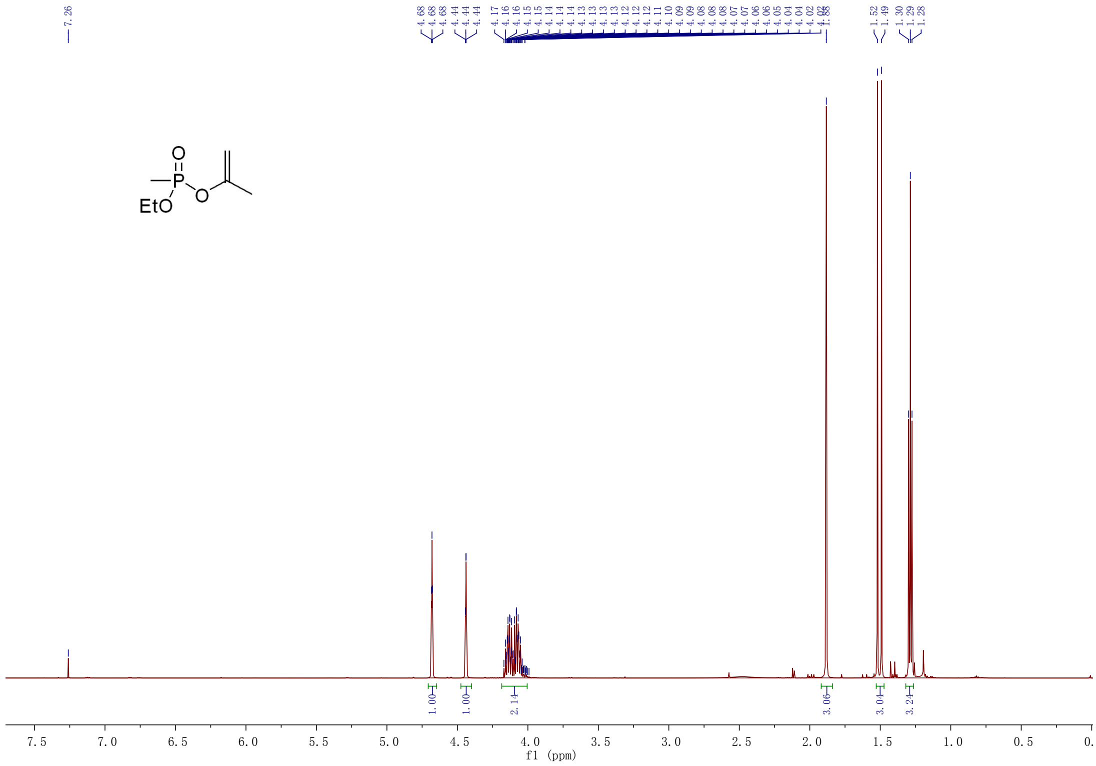

Fig.S 101 ^{1}H NMR of compound **5b**

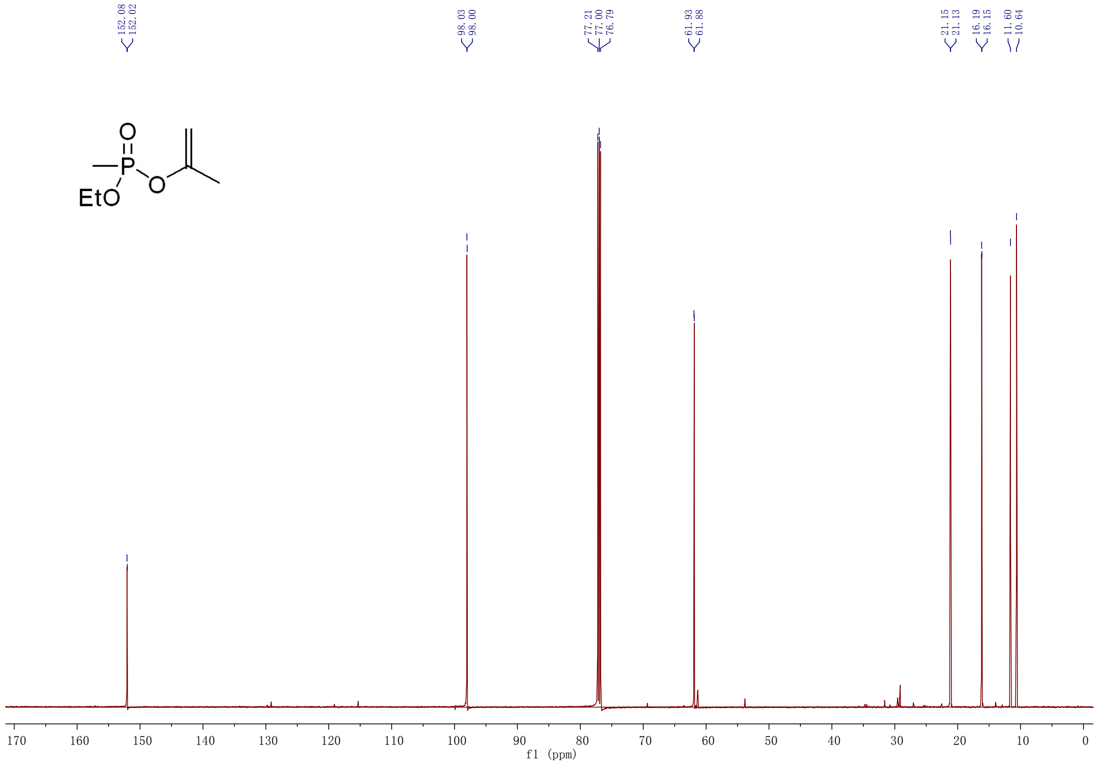

Fig.S 102 ^{13}C NMR of compound **5b**

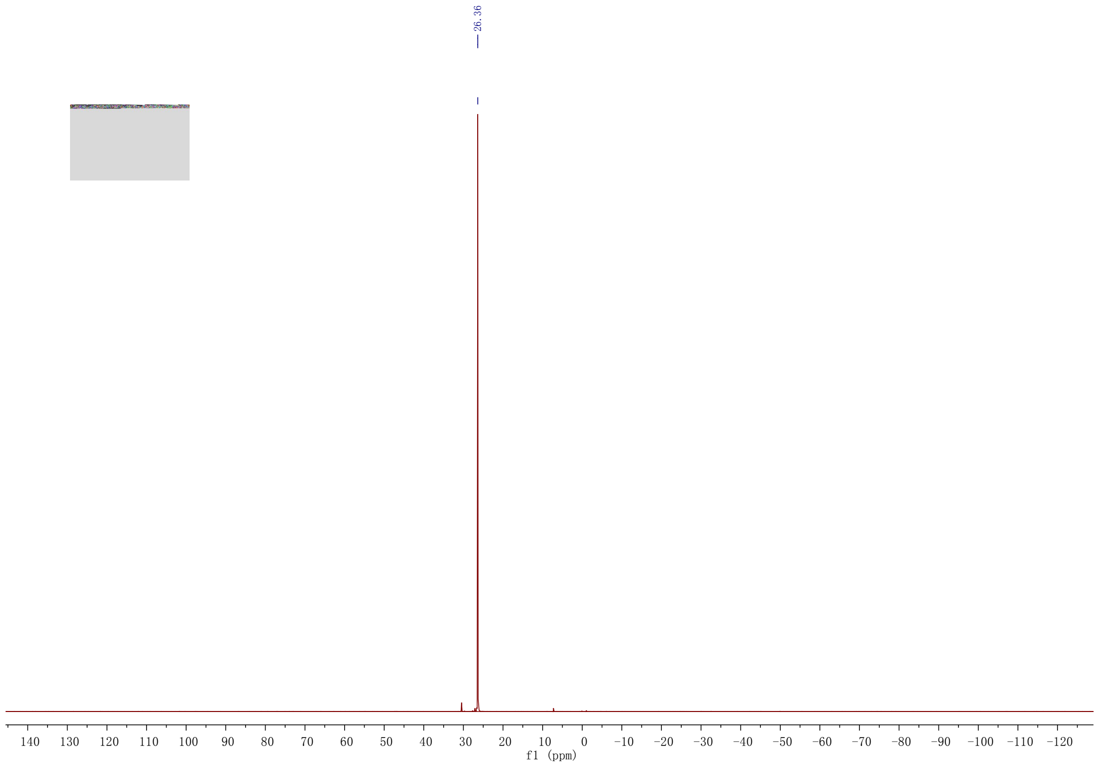

Fig.S 103 ^{31}P NMR of compound **5b**

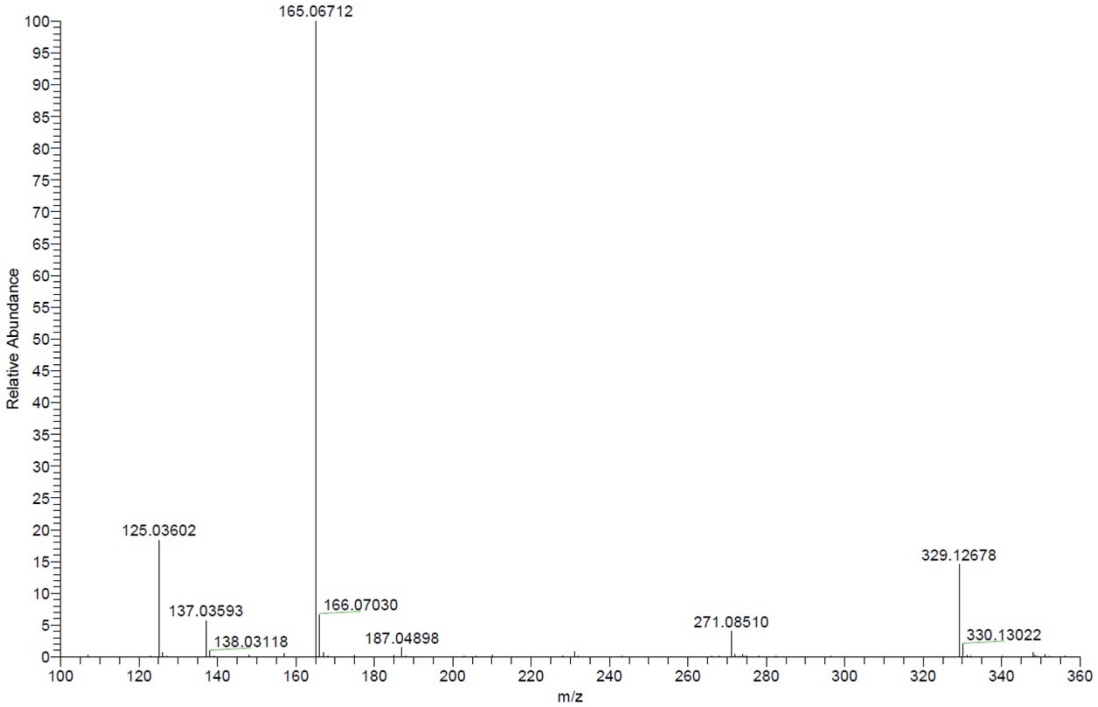

Fig.S 104 HRMS of compound **5b**

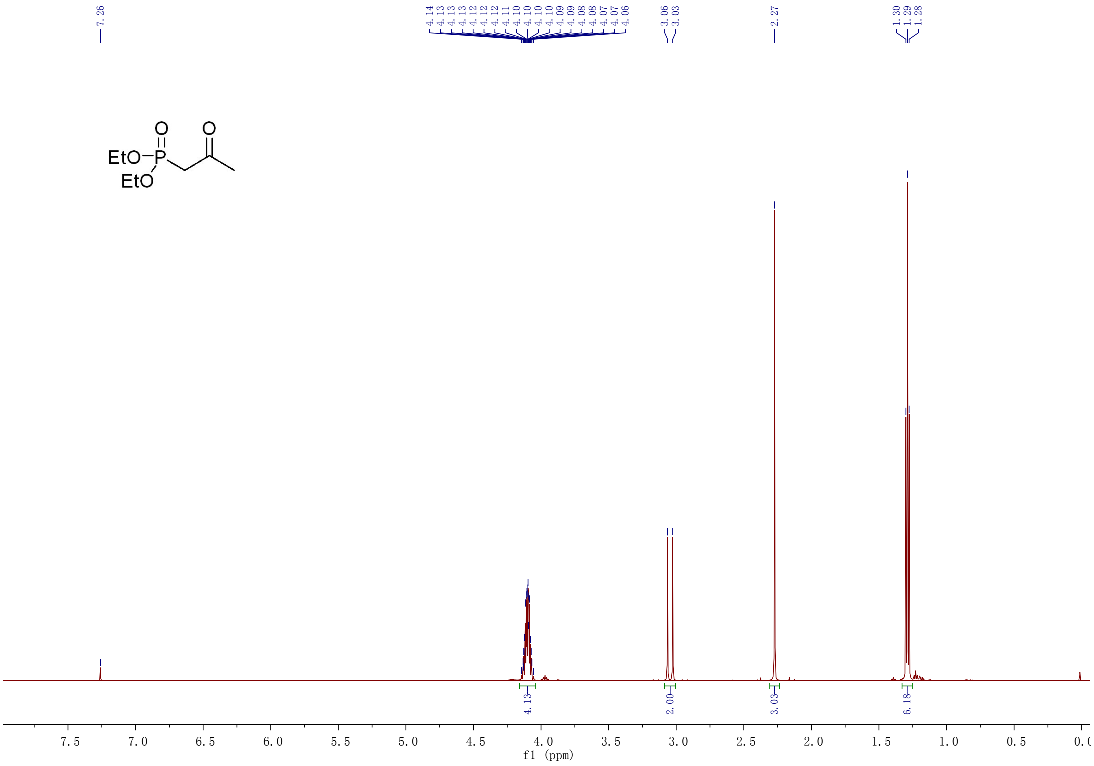

Fig.S 105 ^{1}H NMR of compound **6a**

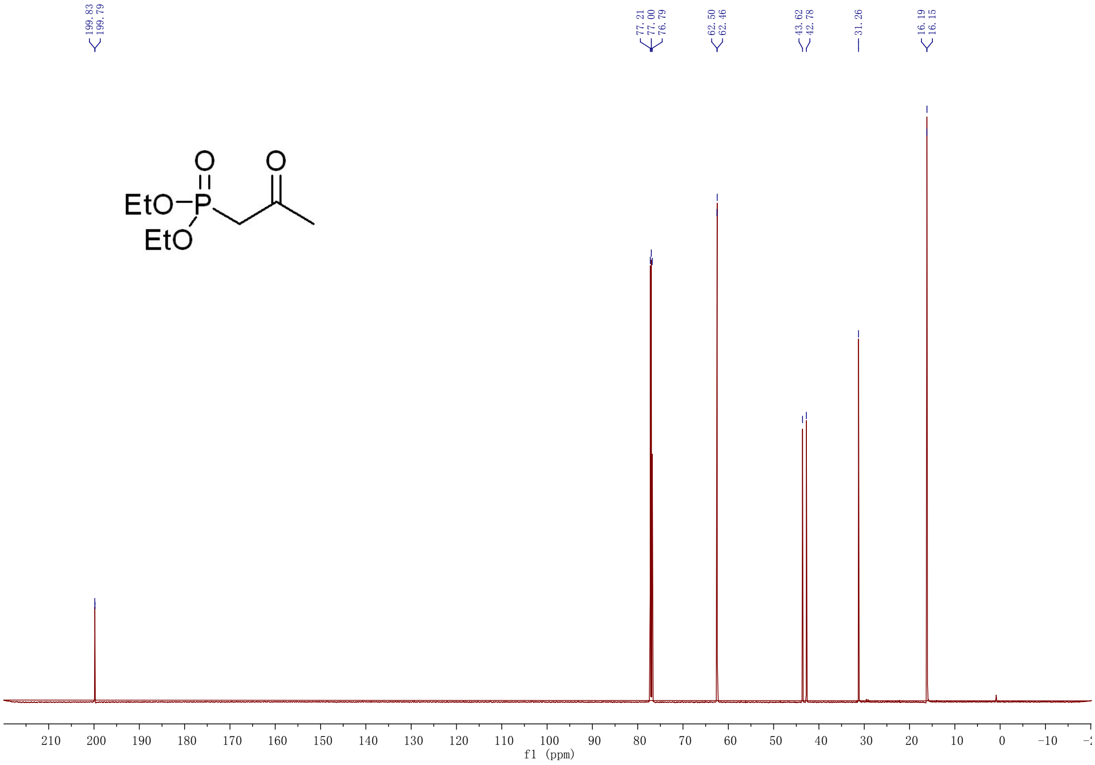

Fig.S 106 ^{13}C NMR of compound **6a**

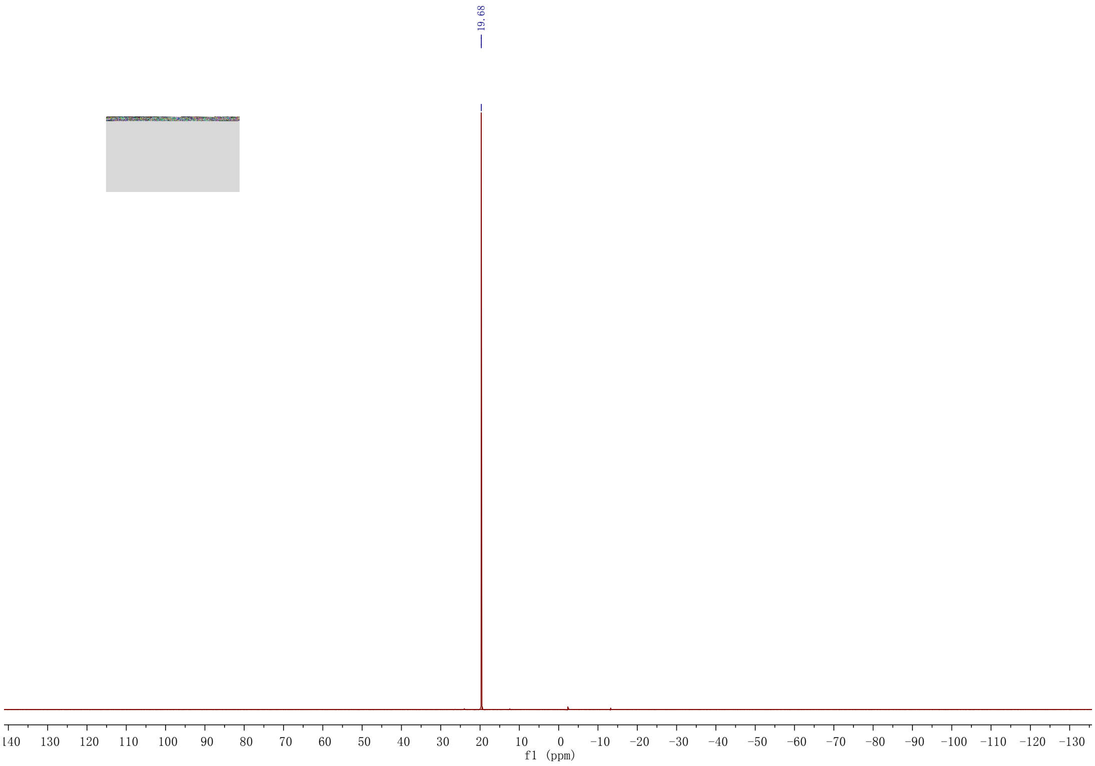

Fig.S 107 ^{31}P NMR of compound **6a**

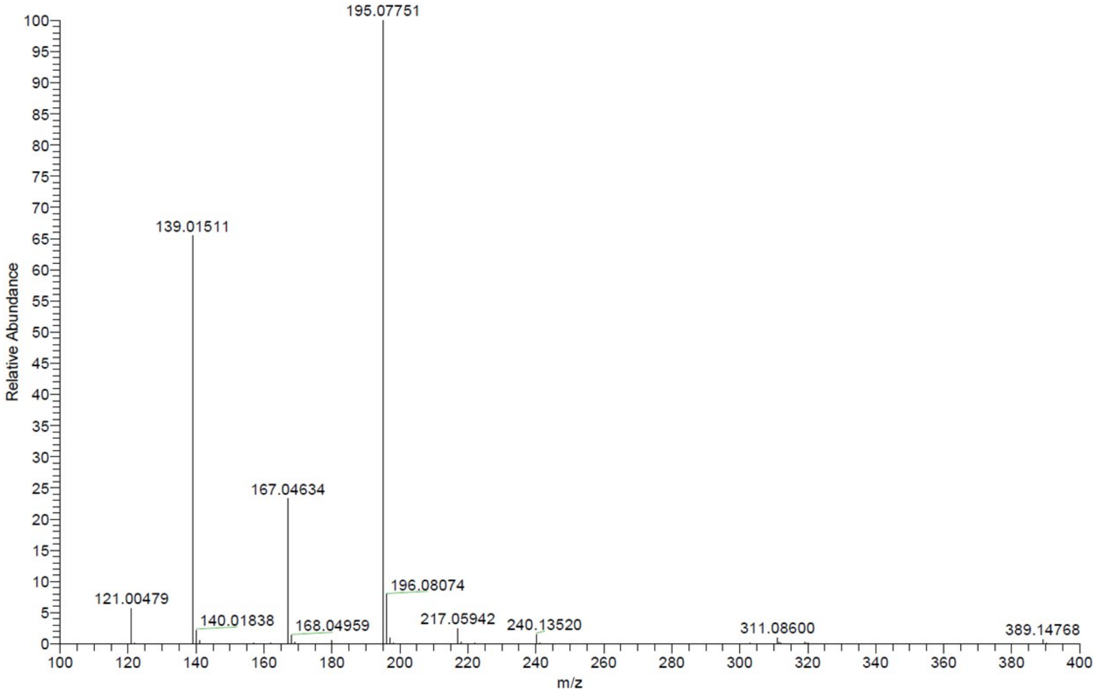

Fig.S 108 HRMS of compound **6a**
